# A web-based tool for the prediction of rice transcription factor function

**DOI:** 10.1093/database/baz061

**Published:** 2019-06-06

**Authors:** Anil Kumar Nalini Chandran, Sunok Moon, Yo-Han Yoo, Yoon-Shil Gho, Peijian Cao, Rita Sharma, Manoj K Sharma, Pamela C Ronald, Ki-Hong Jung

**Affiliations:** 1 Graduate School of Biotechnology and Crop Biotech Institute, Kyung Hee University, Yongin, Republic of Korea; 2 China Tobacco Gene Research Center, Zhengzhou Tobacco Research Institute, Zhengzhou, China; 3 School of Computational and Integrative Sciences, Jawaharlal Nehru University, New Delhi, India; 4 School of Biotechnology, Jawaharlal Nehru University, New Delhi, India; 5 Department of Plant Pathology and the Genome Center, University of California, Davis, CA, USA; 6 Feedstocks Division, The Joint Bioenergy Institute, Emeryville, CA, USA

## Abstract

Transcription factors (TFs) are an important class of regulatory molecules. Despite their importance, only a small number of genes encoding TFs have been characterized in *Oryza sativa* (rice), often because gene duplication and functional redundancy complicate their analysis. To address this challenge, we developed a web-based tool called the Rice Transcription Factor Phylogenomics Database (RTFDB) and demonstrate its application for predicting TF function. The RTFDB hosts transcriptome and co-expression analyses. Sources include high-throughput data from oligonucleotide microarray (Affymetrix and Agilent) as well as RNA-Seq-based expression profiles. We used the RTFDB to identify tissue-specific and stress-related gene expression. Subsequently, 273 genes preferentially expressed in specific tissues or organs, 455 genes showing a differential expression pattern in response to 4 abiotic stresses, 179 genes responsive to infection of various pathogens and 512 genes showing differential accumulation in response to various hormone treatments were identified through the meta-expression analysis. Pairwise Pearson correlation coefficient analysis between paralogous genes in a phylogenetic tree was used to assess their expression collinearity and thereby provides a hint on their genetic redundancy. Integrating transcriptome with the gene evolutionary information reveals the possible functional redundancy or dominance played by paralog genes in a highly duplicated genome such as rice. With this method, we estimated a predominant role for 83.3% (65/78) of the TF or transcriptional regulator genes that had been characterized via loss-of-function studies. In this regard, the proposed method is applicable for functional studies of other plant species with annotated genome.

## Introduction

The Poaceae family contains agronomically important species, including three cereals, rice (*Oryza sativa*), wheat (*Triticum aestivum*) and maize (corn; *Zea mays*), that provide more than half of the total calories consumed by humans. Rice has emerged as an excellent genetic model system for studies of other crops in the family. The genome sequence of many rice species, subspecies and varieties has been completed (1–4). These advances have catalyzed the development of new strategies for characterizing the functions of agronomically important genes. For example, the availability of genome sequence and gene-indexed mutant collections have facilitated both reverse and forward genetics strategies to validate gene functions (e.g. T-DNA insertions, Ds/dSpm tagging, Tos17 tagging and chemical/irradiation mutagenesis) (5–8). Together, these populations carry mutations in ~80% of the predicted rice loci (9–11). The establishment of these mutant resources has paved the way for the application of high-throughput techniques. Genome-wide expression profiles have become an integral part of genome annotation programs. However, despite these advancements, to date, <8% of rice genes have been characterized in detail (12).

Ancient genome duplications indicate that ~50% of all genes related to non-transposable elements in rice are functionally redundant (13). Due to this frequency of redundancy, a mutation in a single gene often results in no or only a subtle change in phenotype. The absence of an altered phenotype in either of the single-gene mutants for a paralog pair suggests that they function in a redundant manner. In this case, generation of a mutant with both paralogs (or more, if multiple paralogs exist) knocked out may reveal phenotypes (14). For example, individual knockouts of rice MADS-box (an acronym of the mini-chromosome maintenance 1 of yeast, agamous of *Arabidopsis*, deficiens of snapdragon and serum response factor of humans) genes, *OsMADS62* and *OsMADS63*, do not reveal altered phenotypes. However, rice plants with both genes knocked out display defects in pollen maturation and germination, revealing their redundant roles in regulating pollen development (15). Although the creation of such multiple mutations can reveal gene function, the labor and expense associated with creating such multiple mutations in rice have hindered rice genetic analysis. The development of CRISPR-Cas9 approaches to mutate several predicted paralogs in a single construct has helped address this problem (16). Here, we present a complementary approach to prioritize candidate genes for functional analysis.

**Figure 1:**
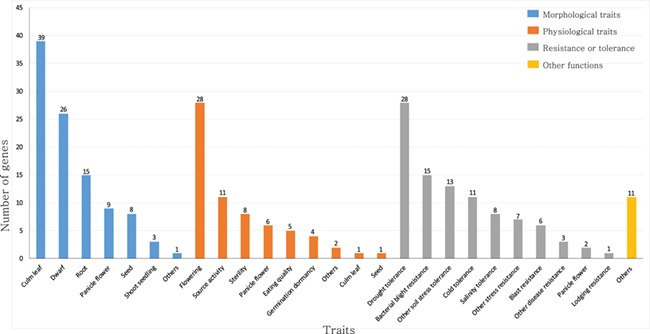
Summary of rice TFs and transcription regulators that have been previously characterized in genetic studies. The genes are classified as those that function in morphological traits, physiological traits, resistance or tolerance and other functions. This list is based on the information available from the OGRO database.

For this study, we focused on rice transcription factors (TFs) and transcriptional regulators (TRs). TFs regulate target gene expression by binding to *cis*-elements in promoter regions, whereas TRs play a regulatory function indirectly through interaction with a basal transcription apparatus or by modulating the accessibility of DNA to TFs via chromatin remodeling (17). TFs and TRs serve key roles in plant development and responses to diverse environmental challenges (18, 19). For example, MADS-box family proteins regulate floral organ formation, identity and flowering time (20–23). The APETALA2/ethylene-responsive element binding protein and NAC (an acronym of no apical meristem, *Arabidopsis* transcription activation factors and cup-shaped cotyledon 2) genes modulate responses to abiotic and biotic stresses (24–30). Members of the homeobox family function in developmental processes such as the organization and maintenance of the shoot apical meristem (SAM) and leaf initiation (31–33) (Figure 1; Supplementary Table S1).

Approximately 2048 of rice genes are predicted to serve as TFs and 328 as TRs (34). Among the predicted TFs, the functions of only 233 TF genes from different families have been elucidated. This characterization relied on diverse genetic approaches, including gene knockdowns, overexpression, mutation and natural variation (35). However, the roles of the remaining TFs are still to be elucidated.

Researchers have constructed several databases to decipher the functions of TFs. For example, the Plant Transcription Factor Database (PlnTFDB; http://plntfdb.bio.uni-potsdam.de/v3.0/) is an integrated catalog that summarizes putatively complete sets of TFs and TRs in plant species (34). The classification of TFs and TRs is based on rules that consider the presence of protein domains and their combinations. These domains are identified by the Pfam protein family database or by hidden Markov model profiles. The Database of Rice Transcription Factors includes TFs and TRs of *O. sativa* L. ssp. *indica* and *O. sativa* L. ssp. *japonica* (36). The Rice Stress-Responsive Transcription Factor Database (http://www.nipgr.res.in/RiceSRTFDB.html) provides expression profiles of TFs and TRs in response to abiotic stress at various developmental stages (37). Despite the usefulness of these databases for the characterization of single genes, the capability of predicting evolutionary relatedness of individual members is lacking. In addition, the information in these databases is limited to sequence and expression data in limited tissues or treatment experiments.

To address these shortcomings and to facilitate functional genomics studies of large gene families, a phylogenomics approach has been proposed (38). In a similar concept, GreenPhylDB provides plant genome-scale phylogenomics analysis to assist ortholog detection (39). Phylogenomics is used to predict functional redundancy or dominance among family members and infer unique functions (13). In this report, we describe generation of a rice TF phylogenomics database, called the Rice Transcription Factor Phylogenomics Database (RTFDB), to analyze rice TF families. RTFDB systematically organizes the functional information of 2048 putative rice TFs and 328 TRs.

Omics data from multiple platforms are integrated into RTFDB. We have also included a co-expression module to assess the co-regulation of TF and TR genes under normal or stress conditions. Meta-expression analysis of anatomical tissues, abiotic/biotic stresses and hormone treatments are also incorporated into the database to help predict the functions of individual family members. For 83.3% (65/78) of the TF or TR genes that had been characterized via loss-of-function studies, we estimated the predominant roles in each family, and our database will facilitate functional genomic studies of TF or TR genes showing featured expression patterns through meta-expression analysis. More detailed data analysis and discussion is presented.

## Results

### Overview of the RTFDB

**Figure 2:**
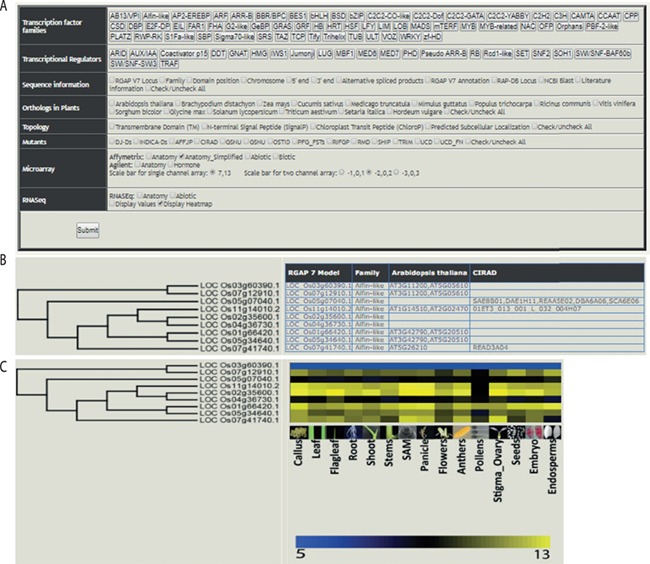
Features of the rice TF phylogenomics database are shown. (**A**) Treeview option enables a phylogenomic analysis of 58 TFs and 22 transcription regulators. (**B**) Selected family can be analyzed by their sequence features, orthologs, topology and available mutants. (**C**) Transcriptomic data, mined and processed from Affymetrix, Agilent or RNA-seq platforms, are integrated into the phylogenetic tree.

The web-based tool, RTFDB, is publicly available at http://ricephylogenomics-khu.org/tf/home.php. RFTDB provides a Treeview option where all TF and TR families are listed (Figure 2A). The selection of a family displays the phylogenetic tree if it comprises three or more members.

Gene annotations, information about orthologs, gene-indexed mutants from 14 repositories, topology and interactome data can be overlaid on the selected family (Figure 2B). Transcriptomic data mined and processed from various platforms in the NCBI gene expression omnibus (GEO; https://www.ncbi.nlm.nih.gov/geo/) (40) can be integrated into the gene family (Figure 2C). These selected data sets can be downloaded under the transcriptome data section for the selected TF family.

The transcriptome analysis in RTFDB has been divided according to platforms used for generating the data. These include microarray data generated using Affymetrix and Agilent platforms. In addition, RNA-Seq data sets for anatomical stages of development samples and abiotic stress treatments are included. To facilitate more robust and comprehensive data analysis, the expression data generated in several different experiments using the same platform were combined and unified under anatomic, abiotic, biotic and hormone categories.

The duplication map displays the genome-wide distribution of all TFs and TRs on rice chromosomes. The duplicated genes lying in segmentally duplicated regions of rice chromosomes are connected through straight lines. The database search option enables users to input gene locus IDs or sequence to retrieve relevant information. Each locus ID is linked to the Rice Genome Annotation Project database (RGAP MSU) (41). The download option can be used to extract gene, protein and cDNA sequences of TFs and TRs.

### Integration of co-expression analysis with functional classification and tissue specificity provides useful information for the further molecular understanding of a TF of interest

**Figure 3:**
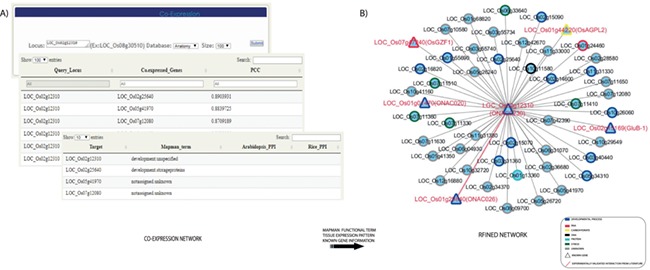
Co-expression analysis of the TFs and transcription regulators revealed candidates that function in seed development. (**A**) Co-expression network for a seed-preferred gene, *LOC_Os02g12310,* is constructed. (**B**) Integration of MapMan terms, tissue-preferred expression pattern and previous literature information on to the network revealed several candidates with proven roles in seed development and other potential candidates for further functional studies related to seed development.

The co-expression analysis tool provides the co-expression module for a TF or TR gene of interest (Figure 3). RTFDB enables an integrated co-expression analysis to predict biologically relevant interactions for a given rice gene under a developmental process or stress treatment by Pearson correlation coefficient (PCC) estimation. Upon querying a TF or TR, the most co-expressed genes are identified, and functional classification using MapMan terms is assigned to the genes in the network (42). Functional assignment to all co-expressed genes reveals the genes that share similar metabolic pathways or biological processes. Co-expressed genes that are assigned unique functional terms with identical tissue expression would be primary targets for further study.

To illustrate an example of candidate screening, we constructed a co-regulated network of a rice seed-specific NAC TF gene, *LOC_Os02g12310*, which was identified via meta-expression analysis of anatomical tissues (Figure 3A). To construct the initial network, we identified 50 co-expressed genes with more than 0.75 PCC value. MapMan terms were then mapped to the interactors and the queried element resulting in seven MapMan terms. Among the results, the term development was over-represented. In addition, it was revealed that co-expressed genes of *LOC_Os02g12310* are related to DNA synthesis, RNA transcriptional regulation, carbohydrate metabolism, protein degradation and stress responses. Consistent with our analysis, it was previously reported that three NAC TF genes in the network physically interact and determine seed size. Specifically, *ONAC02*6 (*LOC_Os01g29840*), *ONAC023* (*LOC_Os02g12310*) and *ONAC020* (*LOC_Os01g01470*) play independent as well as overlapping roles in seed size determination (Figure 3B) (43).

### PCC analysis predicts functional redundancy among paralogous TFs

The PCC between paralogous TF genes indicate levels of similarity in their expression patterns in the analyzed stages of development. This knowledge can be used to predict the redundancy among paralogous genes. To assess the ability of PCC scores to estimate functional redundancy, we performed a PCC analysis with paralogous TF or TR gene pairs that have been previously characterized by loss-of-function studies using knockdown, antisense or RNAi approaches and have expression data available for both the paralogous genes (Supplementary Table S2).

Paralogous genes originate from single gene duplication events and have very high sequence similarity. The functional dominance of a paralogous gene is attributed to the predominance in its expression compared to its duplicated counterpart. We hypothesized that if a gene leads to a defective phenotype on loss of function, it might be a functionally dominant member between the paralogs. Here, we defined a predominant expression pattern as having an estimated PCC of less than 0.5 with the other gene of the paralogous pair.

Out of 233 characterized rice TFs summarized in the Overview of functionally characterized Genes in Rice Online (OGRO) database (35), we selected 92 gene pairs for pairwise correlation analysis whose loss of function resulted in a morphological or physiological change, thereby indicating their dominant contribution in the function. Among these, 39 genes had no close paralogs and, therefore, were not expected to exhibit functional redundancy. For another set of 14 genes, unique probes were not available on the chip, and, therefore, their expression patterns could not be analyzed.

**Figure 4:**
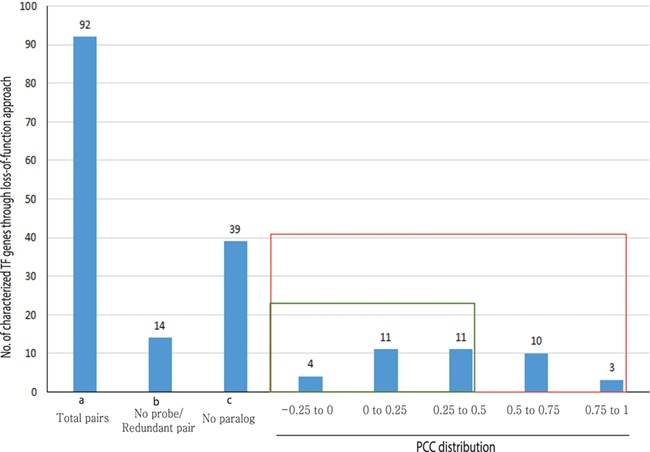
PCC distribution of rice genes that are previously characterized for their role in morphological or physiological traits using loss-of-function studies and their closest paralog genes in the family. PCC score distribution is shown on the *X*-axis; *Y*-axis indicates the number of pairs with given PCC value (red box). The green box indicates pairs that correspond to a smaller PCC range (>0.5). `a’ indicates the total number of characterized TF genes that are related to morphological or physiological traits via loss-of-function studies; `b’, the total number of characterized TFs and their paralog pairs lacking an Affymetrix probe for at least one paralog or without unique probes; `c’, the total number of TF genes with no closest paralog in a subclade of the phylogenetic tree.

**Figure 5:**
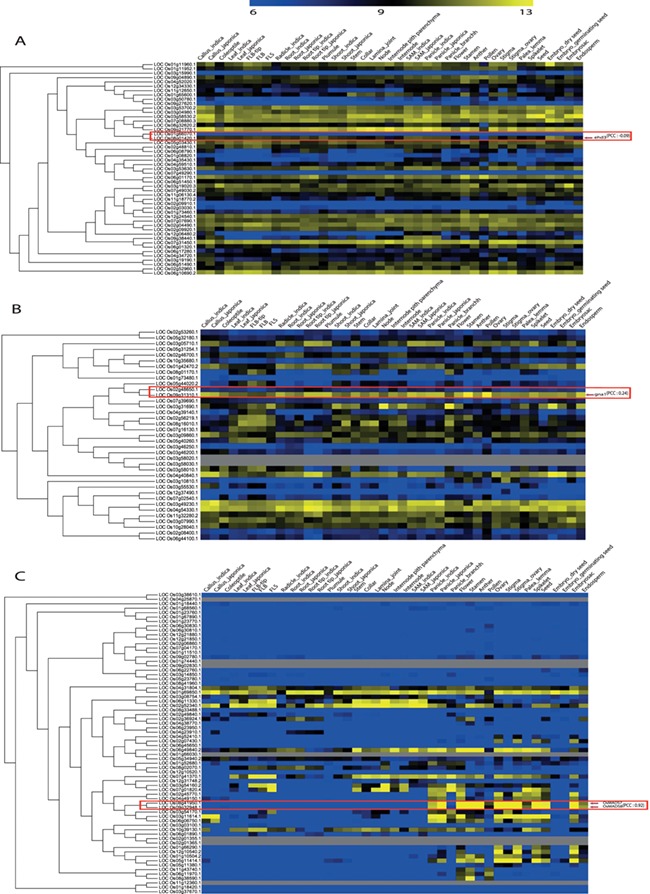
Illustration of phylogenomics analysis utility using characterized genes and their closest paralog for (**A**) Plant homeodomain family, (**B**) GCN5-related N-acetyltransferases family and (**C**) the MADS-box family. Red box indicates paralog pairs and their expression pattern. Red arrow indicates characterized gene(s) of that pair.

For the remaining 39 characterized TF or TR gene pairs, we performed PCC analysis using anatomical meta-expression profiles generated from Affymetrix-based microarray data (Supplementary Table S3). In the case where multiple probes were available for a gene, a probe with the highest expression value was selected for analysis. The distribution of PCC values revealed 4 gene pairs with a correlation between −0.25 and 0.00, 11 pairs with a PCC value of 0.00–0.25, 11 pairs with a PCC value of 0.26–0.50 and 10 pairs with a PCC value of 0.51–0.75. Whereas, only 3 pairs exhibited correlation in the range of 0.75–1.00 (Figure 4). This analysis demonstrated the predominant expression of 26 genes that share little correlation with their paralogs (PCC < 0.5; green box in Figure 4). Therefore, in line with our hypothesis, including 39 genes belonging to a clade consisting of a single member, a predominant role was suggested for 83.3% (65/78) of the TF or TR genes that had been characterized via loss-of-function studies through integrated phylogenomics and pairwise PCC analysis. To illustrate the utilization of phylogenomics and pairwise PCC analysis to predict redundancy or predominance in gene functions, we have chosen 3 previously characterized TFs and their paralogs from each of the 3 PCC ranges, i.e. −0.25–0.00, 0.00–0.25 and > 0.75. These include *early heading date 3* (*ehd3*), which shares no correlation (PCC value, −0.09) with its closest paralog *LOC_Os01g66070*; *acetyltransferase 1* (*gna1*) (*LOC_Os09g31310*) with a distinct expression pattern compared to its paralog *LOC_Os02g48650* (PCC value, 0.24); and paralogous genes *OsMADS8* and *OsMADS7*, which shows very similar expression patterns (PCC value, 0.92) (Figure 5). As expected, the functional dominance of *Ehd3* and *Gna1* was well supported by the phylogenomic analysis.

### Meta-expression analysis of TFs to identify candidates for diverse applications

We retrieved sequence information for putative rice TF and TR genes that are summarized in PlnTFDB and conducted a meta-expression analysis. Our in-house meta-expression databases included Affy_anatomy, Affy_abiotic and Affy_biotic data sets, which were generated from Affymetrix array-based data to analyze gene expression under anatomy, abiotic and biotic stress treatments, respectively. The response of TF- or TR-encoding genes to hormones was analyzed using Agilent array-based expression data sets (44). Affymetrix-based data comprises expression profiles for 35 421 genes while 25 044 genes are represented in the Agilent-based hormone data. We were able to analyze expression profiles of 2139 and 1822 TF and TR genes using the Affymetrix and Agilent data sets, respectively.

**Figure 6:**
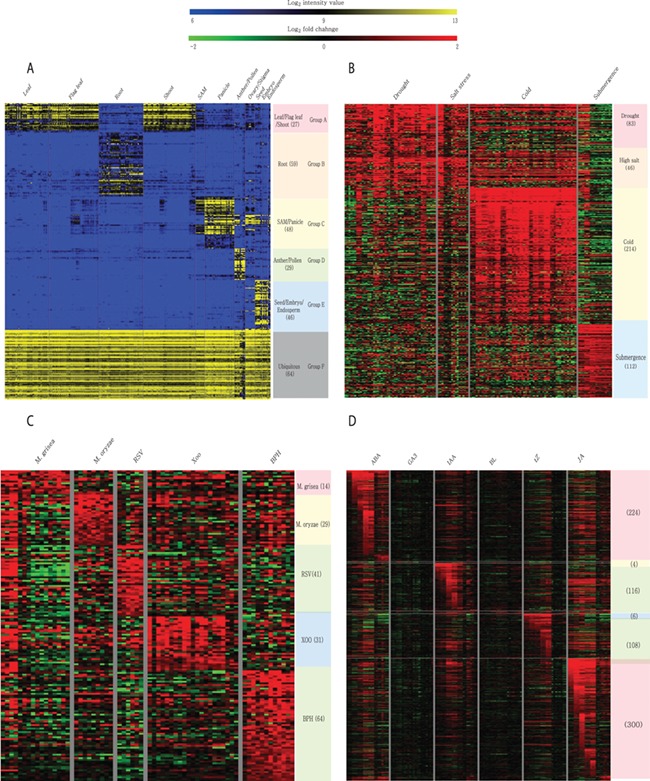
Heatmaps of featured gene expression groups that are derived from meta-expression analysis of (**A**) anatomical samples, (**B**) abiotic stress samples, (**C**) biotic stress samples and (**D**) hormone-treated samples. The number of genes per group is shown in parentheses after the group name. For stress and hormone-treated featured groups, a statistical cut-off of >2 log_2_ fold-change at *P* < 0.05 was used.

Meta-expression analysis revealed that 273 genes are preferentially expressed in specific tissues or organs. Among them, 27 genes are preferentially expressed in above-ground vegetative parts, 59 in the root, 48 in SAMs and panicles, 29 in anthers and pollen, 46 in seeds and 64 genes are ubiquitously expressed in all analyzed tissues (Figure 6A; Supplementary Table S4). To suggest the potential candidates for genetic studies that solely function in monocots or rice, we identified monocot and rice-divergent genes (Supplementary Table S5). Similar to groups having different anatomical expression patterns, 455 genes showed a differential expression pattern in response to 4 abiotic stresses. These include 83 genes induced by drought stress, 46 by salinity stress, 214 by cold stress and 112 by submergence stress (Figure 6B; Supplementary Table S6). In addition, 179 genes were responsive to infection of various pathogens. Among them, 14 genes were responsive to *Magnaporthe grisea*, 29 to *Magnaporthe oryzae*, 41 to rice stripe virus, 31 to *Xanthomonas oryzae* pv. *oryzae* and 64 genes to brown planthopper (Figure 6C; Supplementary Table S7). Finally, 512 genes showed differential accumulation in response to at least one of the six hormone treatments (i.e. abscisic acid, jasmonic acid, indole acetic acid, trans-Zeatin, gibberellin and brassinolide) (Figure 6D; Supplementary Table S8). A detailed description of genes in each meta-group is summarized in the notes of Supplementary Tables S4–S8.

## Materials and methods

### Identification of rice TFs and TRs

Information regarding genome-wide TFs and TRs in rice was retrieved from PlnTFDB (34). Sequences of these putative TFs and TRs were downloaded from RGAP MSU v7. The alternative splicing products of a locus were addressed with representative loci with transcript evidence (41). Obsolete RGAP MSU loci were eliminated from the list.

### Classification of tissue-preferential or stress-responsive featured groups

Microarray data sets for meta-expression analysis were downloaded from NCBI GEO Affymetrix collections. Expression profiles in anatomical samples were retrieved from the Rice Oligonucleotide Array Database (45). In addition, 145 abiotic stress transcriptomes and 103 biotic stress transcriptomes were downloaded and integrated into the resource. For the hormone transcriptomes, Agilent microarray samples under accession GSE39429 described in RicExpro (44) were used. Affymetrix data sets were normalized with the R package affy using the MAS5 method. For anatomical samples, normalized intensity values were log_2_-transformed, and log_2_ fold-changes were estimated for stress transcriptomes. All probe IDs were mapped to the RGAP MSU loci based on the sequence similarity. For genes with two or more probes in the array, the probe with the highest mean expression across all samples was selected.

### Raw data preprocessing and expression quantification for RNA-Seq anatomy data sets

We downloaded 25 raw single-end data sets that consist of 9 tissue types from DNA Data Bank of Japan (DDBJ) (Supplementary Table S9). After read preprocessing with Trimmomatic (46), quality filtered reads were mapped to rice reference genome IRGSP-1.0. Mapped reads to genomic regions were estimated using featureCounts (47). The raw counts from the featureCounts were fed to DESeq2 package for count normalization (48) and integrated into the database.

### Co-expression analysis

Using the microarray data sets, we identified the most co-expressed candidates of an input gene by the PCC method. To integrate probable protein interaction information from previous studies to the co-expressed genes, we obtained rice protein–protein interaction (PPI) information from the IntAct molecular interaction database (49) and also retrieved *Arabidopsis* PPI from The *Arabidopsis* Information Resource (50).

### Statistical analysis

Tissue-specific or tissue-preferential genes were estimated with the Tau method, which was previously shown to be superior to other methods (51). A Tau score closer to zero indicates broad gene expression. Therefore, we used a cut-off of Tau <0.15 to define ubiquitously expressed genes. Because some tissue types are related, e.g. leaf and flag leaf or seed and endosperm, we chose a less stringent cut-off of Tau >0.6 for preliminary screening of tissue-preferential genes. From this initial gene set, we applied *K*-means clustering by applying a Euclidean distance method and further refined the screened group. As an alternative, we also used Sprent’s parametric method (52), which effectively detects specific genes from two or three correlated tissue types. For stress-responsive genes, fold-change values for similar profiles were combined, and a one-sample, one-tailed *t*-test was conducted to determine significant gene expression, i.e. >2-fold at *P* < 0.05 for abiotic, biotic stress and hormone treatments. Gene clustering analysis was performed with MeV software (http://mev.tm4.org/#/welcome), and the R program was used for statistical analysis.

### Data source

Sequence information on TFs and TRs were retrieved from RGAP MSU v7. Orthologs were identified with InParanoid v4.1 (53) and the OMA browser (54). These data sets were used to define monocot- and rice-divergent genes. In addition, TMHMM v2 (55), the plant-specific myristoylation predictor (56), SignalP v3 (57) and ngLOC (58) were used to predict transmembrane domains, N-terminal myristoylation sites, N-terminal signal peptides and protein subcellular localization, respectively. Gene-indexed mutant information was collected from the literature (59) and from the latest publications on the generation of 1504 mutants in `Kitaake’ rice (11). To construct phylogenetic trees, we aligned the protein sequences from representative RGAP MSU models via ClustalW v2 (60). Trees were generated by using the PhyML maximum likelihood method with the JTT model (61). A gene expression heatmap was generated with the JpGraph PHP library (http://jpgraph.net/).

### Database architecture

The database was constructed with PHP server-side programming language (http://php.net/), and the various data sets, including transcriptome data, were stored in a MySQL relational database (http://www.mysql.com/). This resource is hosted on the Apache HTTP Server (https://httpd.apache.org/). Interactive websites were created with HTML5, CSS and JavaScript. All these utilities are based on the Linux operating system.

## Funding

Next-Generation BioGreen 21 Program (PJ01366401 and PJ01369001 to K.H.J.); Rural Development Administration; National Research Foundation of Korea (2018R1A4A1025158 to K.H.J.); The Collaborative Genome Program of the Korea Institute of Marine Science and Technology Promotion (KIMST) funded by the Ministry of Oceans and Fisheries (MOF) (No. 2018043004 to K.H.J.); US Department of Energy, Office of Science, Office of Biological and Environmental Research; Office of Science of the US Department of Energy (DE-AC02-05CH11231); National Institutes of Health (GM59962 and GM122968 to P.C.R.); National Science Foundation (IOS-1237975 to P.C.R.).

*Conflict of interest*. None declared.

## Supplementary Material

Supplementary_note_baz061

Supplementary_Tables_baz061

## Notes

**Database URL**: http://ricephylogenomics-khu.org/tf/home.php
