# Supplementary material for: A web-based tool for the prediction of rice transcription factor function
Source: Database (Oxford). 2019 Jun 6;2019:baz061. doi: 10.1093/database/baz061 (PMC6553503; doi:10.1093/database/baz061)
Supplement: Supplementary_note_baz061 [file supplementary_note_baz061.docx]

***Group A: Genes preferentially expressed in above-ground vegetative organs***

We identified 27 genes that are preferentially expressed in above-ground vegetative parts such as leaves, flag leaves, and shoots (Figure 7A, Table S5). This group (Group A) includes three genes each from the bHLH, myeloblastosis (MYB)-related, and GCN5-related N-acetyltransferase families. Altogether, this group contains candidates from 15 families including *LOC_Os06g24070*, which encodes the G2-like family *OsGLK1* that induces chloroplast development in non-green cells in rice through ectopic expression (Nakamura *et al.*, 2009). Two *OsGLK1* orthologs in Arabidopsis (*ATGLK1* and *ATGLK2*) also regulate chloroplast development (Fitter *et al.*, 2002). A monocot-divergent gene from Group A, *LOC_Os10g41100*, has orthologs in *Sorghum* and *Brachypodium* that encode *CONSTANS*, a photoperiod-regulated activator of flowering (Higgins *et al.*, 2010; Yang *et al.*, 2014). Information about previously characterized genes from Group A suggest that the remaining uncharacterized genes are novel targets for studying photosynthesis or leaf development.

***Group B: Genes preferentially expressed in roots***

We identified 59 genes in Group B that are preferentially expressed in the roots (Figure 7A, Table S5). These include WRKY, MYB, Cysteine-2/Histidine-2 (C2H2), AP2-EREBP, and NAC family members, which constitute 71% of the root-preferred genes. This group also contains nine genes that are monocot-specific while four have no sequence similarity with any other species, implying that they are rice-divergent (Table S6). Because expression is preferential to the underground organ, a subset of genes in this group might be involved in biotic-stress tolerance or nutrient homeostasis. In support of this assumption, two genes (*LOC_Os01g53260* and *LOC_Os08g13840*) are induced under pathogen infection, also suggesting possible roles in biotic-stress responses. *OsbHLH133*, previously characterized Group B gene, is induced by iron deficiency and regulates iron distribution between the root and shoot (Wang *et al.*, 2013). *Fer-like iron deficiency-induced transcription factor*, an Arabidopsis ortholog of *LOC_Os04g31290*, is essential for iron uptake in Arabidopsis roots (Mai *et al.*, 2015). Another potential candidate for functional studies is *LOC_Os01g02110*, which has an Arabidopsis ortholog, *root hair defective 6*, that is involved in root hair formation (Grierson & Schiefelbein, 2002). These genetic studies indicate that Group B genes are potential candidates for research related to root morphology and nutrient balance.

***Group C: Genes preferentially expressed in the shoot apical meristem (SAM) and panicles***

We determined that the expression of 48 genes is high and preferential to the SAM and panicles, including nine previously characterized MADS-box genes (Figure 7A, Table S5). We assigned all of them to Group C. Genes from this group have roles in the axillary meristem, ligule and auricle formation, awn development, panicle branching, and tillering. Teosinte branched 1/cycloidea/proliferating cell factor 1 family candidates are known regulators of cell de-differentiation and meristem formation (Ikeda & Ohme-Takagi, 2014). We identified two Teosinte branched 1/cycloidea/proliferating cell factor 1 genes (*LOC_Os04g44440* and *LOC_Os03g49880*) with meristem-preferred expression. A maize ortholog (*FEA4*) of the Group C basic leucine zipper (bZIP) member *LOC_Os06g15480* also promotes meristem periphery differentiation, and its mutation results in fasciated ears and tassels (Pautler *et al.*, 2015). This pattern of expression is in accord with that of previously characterized genes, thereby indicating that members of Group C might be important in determining shoot meristem formation and panicle architecture.

***Group D: Genes preferentially expressed in anthers/pollen***

We identified 29 genes in Group D, including five monocot-specific and two rice-divergent candidates, that are predominantly expressed in anthers or pollen grains (Figure 7A, Tables S5). Two genes already characterized in this group, *LEC2* and *FUSCA3 Like 1*, regulate flowering time (Peng *et al.*, 2008). Among other group members, six genes are encoded by C2H2 TFs. These genes are associated with floral organ identity, including *stamenless 1*, which is induced during early flower development and is involved in lodicule and stamen identity (Xiao *et al.*, 2009). A rice MADS-box gene, *OsMADS13*, that encodes for the agamous protein determines the reproductive organs such as stamens, carpels, and ovules (Dreni & Kater, 2014). Group D MADS-box genes encode a putative agamous and offer a potential target for floral organ studies. Notably, orthologs of these MADS-box genes (*LOC_Os06g11970* and *LOC_Os11g43740*) in Arabidopsis (*AGL30*, *AGL65*, *AGL66*, and *AGL104*) form heterodimers that have a partially redundant role in late-stage pollen development and pollen tube growth (Adamczyk & Fernandez, 2009). Successful reproduction is dependent on the release of viable pollen grains and investigating Group D genes might reveal new candidates that can directly increase crop yields.

***Group E: Genes preferentially expressed in seeds***

We identified that 46 genes are preferentially expressed in the whole seed or in certain parts, such as the embryo and endosperm (Figure 7A, Tables S5). These genes are designated as Group E. Overrepresented family members with seed-, embryo-, or endosperm-preferred expression include eight CCAAT, seven NAC, and six MYB genes. Among the 46 genes in this group, 18 are monocot-specific and six are specific to rice (Table S6). This highlights the diversification in grain production pathways between monocots and dicots. Especially in the early stage of endosperm development, grasses have considerable uniformity (Sabelli & Larkins, 2009). Previously characterized genes in this group are related to seed development, ovule identity, and determining storage proteins and starch content (Dreni *et al.*, 2007; Kawakatsu *et al.*, 2009; Yin & Xue, 2012). Orthologs in other species also regulate floral organ development, fertility, and other processes of seed development. For example, an ortholog of *LOC_Os01g68370* in maize, *vívíparous-I*, controls the seed maturation phase (McCarty *et al.*, 1989). The higher proportion of monocot- and rice-divergent genes in this group demonstrate the existence of a specialized mechanism for grain formation in cereal crops.

***Group F: Genes exhibiting ubiquitous expression***

We identified 64 housekeeping genes that are expressed in all types of rice tissues. Group F consists of candidates from 33 TF families (Figure 7A, Tables S5). Previous literature analysis indicates that despite the multiple tissue expression patterns, many genes have specific functional roles. For example, genes in the group have reported role in phosphate starvation, leaf and root morphology, dwarfism, flowering, and grain size determination (Mao *et al.*, 2006; Zhang *et al.*, 2011; Hu *et al.*, 2012; Sun *et al.*, 2012; Li *et al.*, 2014). Hence, it is difficult to get functional clues of group F genes merely based on their expression pattern.

***Group G*: Genes induced under abiotic or biotic stresses**

We identified 455 genes that are significantly induced by at least two-folds (*P*<0.05; Figure 7B, Table S7) in response to abiotic stress. Including multiple stress responsive genes, they include 83 genes related to drought, 46 to salt stress, 214 to low-temperature and 112 genes responsive to submergence stress or anoxic stress. Overall, MYB, AP2-EREBP, bZIP, Homeobox, heat shock factors, C2H2, and NAC family genes constitute 67% of the drought-induced TFs. Abscisic acid (ABA) is a key regulator of drought stress, being mediated by abscisic acid-responsive element-dependent gene expression (Nakashima *et al.*, 2014). Scans of the sequence 1 kb upstream of our candidates in the Rice Annotation Project Database (Sakai *et al.*, 2013) revealed cis-elements, (C/T)ACGTGGC, G-box sequence CACGTG, and CGCGTG-containing abscisic acid-responsive elements, which are features of several ABA- and stress-regulated genes (Choi *et al.*, 2000). Among the 17 genes from our drought-induced set that have been previously characterized, 10 are regulated by drought (Table S7). In particular, the C2H2 and MYB family genes (*LOC_Os07g39960* and *LOC_Os04g42950*, respectively) show root-preferred expression, while a drought-responsive rice-divergent C3H family gene (*LOC_Os01g53650*) showed endosperm-preferred expression. We determined that 33 of the 46 salt stress-induced genes are also induced by drought.

The C-repeat binding factors are members of the AP2-EREBP family that show extreme stress adaptations (Ryu *et al.*, 2014). Consistently, meta-expression analysis revealed 36 AP2-EREBP members that are highly up-regulated in response to low temperature. Other dominant TF families that respond to cold stress include 21 WRKY genes, 21 MYB/MYB-related genes, and 15 NAC genes.

Meta-expression analysis indicated that genes encoding ethylene responsive factors constitute nearly 20% (21 of 108 genes) of the submergence-induced candidates. Anatomical expression of submergence-induced genes showed that eight genes have root-preferred expression and another eight are preferentially expressed at the SAM. Longitudinal diffusion of O_2_ to the well-developed aerenchyma system of the roots is one type of stress adaptation (Nishiuchi *et al.*, 2012). Therefore, submergence-induced root- and SAM-preferred genes might have roles in sustaining plant growth and survival under waterlogged conditions.

We also identified 179 genes that are responsive to biotic stress and are induced at least two-folds (*P*<0.05; Figure 7C, Table S8). Biotic stress-responsiveness was determined from expression profiles of pathogen-infected samples and included 14 genes that are significantly induced by *Magnaporthe grisea* infection, 29 by *Magnaporthe oryzae*, 41 by rice stripe virus, 31 by *Xanthomonas oryzae* pv. *Oryzae*, and 64 in response to the brown planthopper. We also identified 56 candidates that are common to both biotic and abiotic stresses and that might be involved in overlapping transcriptomic responses.

***Group H*: Genes induced under hormone treatment**

At least in one specific time-point, 512 TFs were up-regulated by more than two-folds (*P*>0.05) in response to hormone treatment (Figure 7D, Table S9). In the roots, ABA treatment induced 208 genes, jasmonic acid induced 288, indole acetic acid induced 113, and trans-Zeatin induced 106 genes. A few genes responded to treatment with gibberellin (three genes) or brassinolide (four genes) in root tissues. Whereas, in the shoots, only 76 genes were induced by ABA treatment, three by brassinolide, seven by indole acetic acid, 62 by jasmonic acid, and four by trans-Zeatin treatment. This contrast in hormone responsiveness between tissue types indicate that the root transcriptome is affected more severely compared with that of shoots in response to hormone treatments.

**References**

**Adamczyk BJ, Fernandez DE**. **2009**. MIKC* MADS Domain Heterodimers Are Required for Pollen Maturation and Tube Growth in Arabidopsis. *Plant Physiology* **149**: 1713–1723.

**Choi H-I, Hong J-H, Ha J-O, Kang J-Y, Kim SY**. **2000**. ABFs, a family of ABA responsive element binding factors. *Journal of Biological Chemistry* **275**: 1723–1730.

**Dreni L, Jacchia S, Fornara F, Fornari M, Ouwerkerk PBF, An G, Colombo L, Kater MM**. **2007**. The D-lineage MADS-box gene OsMADS13 controls ovule identity in rice. *Plant Journal* **52**: 690–699.

**Dreni L, Kater MM**. **2014**. MADS reloaded: Evolution of the AGAMOUS subfamily genes. *New Phytologist* **201**: 717–732.

**Fitter DW, Martin DJ, Copley MJ, Scotland RW, Langdale JA**. **2002**. GLK gene pairs regulate chloroplast development in diverse plant species. *Plant Journal* **31**: 713–727.

**Grierson C, Schiefelbein J**. **2002**. Root Hairs. *The Arabidopsis Book* **1**: e0060.

**Higgins JA, Bailey PC, Laurie DA**. **2010**. Comparative genomics of flowering time pathways using brachypodium distachyon as a model for the temperate Grasses. *PLoS ONE* **5**.

**Hu Y, Liu D, Zhong X, Zhang C, Zhang Q, Zhou D-X**. **2012**. CHD3 protein recognizes and regulates methylated histone H3 lysines 4 and 27 over a subset of targets in the rice genome. *Proceedings of the National Academy of Sciences* **109**: 5773–5778.

**Ikeda M, Ohme-Takagi M**. **2014**. TCPs, WUSs, and WINDs: families of transcription factors that regulate shoot meristem formation, stem cell maintenance, and somatic cell differentiation. *Frontiers in Plant Science* **5**: 3–6.

**Kawakatsu T, Yamamoto MP, Touno SM, Yasuda H, Takaiwa F**. **2009**. Compensation and interaction between RISBZ1 and RPBF during grain filling in rice. *Plant Journal* **59**: 908–920.

**Li S, Wang C, Zhou L, Shou H**. **2014**. Oxygen deficit alleviates phosphate overaccumulation toxicity in OsPHR2 overexpression plants. *Journal of Plant Research* **127**: 433–440.

**Mai H-J, Lindermayr C, Toerne C von, Fink-Straube C, Durner J, Bauer P**. **2015**. Iron and FER-LIKE IRON DEFICIENCY-INDUCED TRANSCRIPTION FACTOR-dependent regulation of proteins and genes in Arabidopsis thaliana roots. *Proteomics* **15**: 3030–3047.

**Mao C, Wang S, Jia Q, Wu P**. **2006**. OsEIL1, a Rice Homolog of the Arabidopsis EIN3 Regulates the Ethylene Response as a Positive Component. *Plant Molecular Biology* **61**: 141–152.

**McCarty DR, Carson CB, Stinard PS, Robertson DS**. **1989**. Molecular Analysis of viviparous-1: An Abscisic Acid-Insensitive Mutant of Maize. *Plant Cell* **1**: 523–532.

**Nakamura H, Muramatsu M, Hakata M, Ueno O, Nagamura Y, Hirochika H, Takano M, Ichikawa H**. **2009**. Ectopic overexpression of the transcription factor osglk1 induces chloroplast development in non-green rice cells. *Plant and Cell Physiology* **50**: 1933–1949.

**Nakashima K, Yamaguchi-Shinozaki K, Shinozaki K**. **2014**. The transcriptional regulatory network in the drought response and its crosstalk in abiotic stress responses including drought, cold, and heat. *Frontiers in Plant Science* **5**: 1–7.

**Nishiuchi S, Yamauchi T, Takahashi H, Kotula L, Nakazono M**. **2012**. Mechanisms for coping with submergence andwaterlogging in rice. *Rice* **5**: 1–14.

**Pautler M, Eveland AL, LaRue T, Yang F, Weeks R, Lunde C, Je B Il, Meeley R, Komatsu M, Vollbrecht E, *et al.*** **2015**. FASCIATED EAR4 Encodes a bZIP Transcription Factor That Regulates Shoot Meristem Size in Maize. *The Plant Cell Online* **27**: 104–120.

**Peng L-T, Shi Z-Y, Li L, Shen G-Z, Zhang J-L**. **2008**. Overexpression of transcription factor OsLFL1 delays flowering time in Oryza sativa. *Journal of Plant Physiology* **165**: 876–885.

**Ryu JY, Hong SY, Jo SH, Woo JC, Lee S, Park CM**. **2014**. Molecular and functional characterization of cold-responsive C-repeat binding factors from Brachypodium distachyon. *BMC Plant Biology* **14**: 1–15.

**Sabelli PA, Larkins BA**. **2009**. The Development of Endosperm in Grasses. *Plant Physiology* **149**: 14–26.

**Sakai H, Lee SS, Tanaka T, Numa H, Kim J, Kawahara Y, Wakimoto H, Yang CC, Iwamoto M, Abe T, *et al.*** **2013**. Rice annotation project database (RAP-DB): An integrative and interactive database for rice genomics. *Plant and Cell Physiology* **54**.

**Sun C, Fang J, Zhao T, Xu B, Zhang F, Liu L, Tang J, Zhang G, Deng X, Chen F, *et al.*** **2012**. The Histone Methyltransferase SDG724 Mediates H3K36me2/3 Deposition at MADS50 and RFT1 and Promotes Flowering in Rice. *The Plant Cell* **24**: 3235–3247.

**Wang L, Ying Y, Narsai R, Ye L, Zheng L, Tian J, Whelan J, Shou H**. **2013**. Identification of OsbHLH133 as a regulator of iron distribution between roots and shoots in Oryza sativa. *Plant, Cell and Environment* **36**: 224–236.

**Xiao H, Tang J, Li Y, Wang W, Li X, Jin L, Xie R, Luo H, Zhao X, Meng Z, *et al.*** **2009**. STAMENLESS 1, encoding a single C2H2 zinc finger protein, regulates floral organ identity in rice. *Plant Journal* **59**: 789–801.

**Yang S, Weers BD, Morishige DT, Mullet JE**. **2014**. CONSTANS is a photoperiod regulated activator of flowering in sorghum. *BMC Plant Biology* **14**: 148.

**Yin L-L, Xue H-W**. **2012**. The MADS29 Transcription Factor Regulates the Degradation of the Nucellus and the Nucellar Projection during Rice Seed Development. *The Plant Cell* **24**: 1049–1065.

**Zhang C-Q, Xu Y, Lu Y, Yu H-X, Gu M-H, Liu Q-Q**. **2011**. The WRKY transcription factor OsWRKY78 regulates stem elongation and seed development in rice. *Planta* **234**: 541–554.
