# Supplementary material for: A web-based tool for the prediction of rice transcription factor function
Source: Database (Oxford). 2019 Jun 6;2019:baz061. doi: 10.1093/database/baz061 (PMC6553503; doi:10.1093/database/baz061)
Supplement: Supplementary_Tables_baz061 [file supplementary_tables_baz061.docx]

**Table S1.** Summary of unannotated genes which have been functionally characterized from the OGRO database.

| **Locus_ID Characteristics Gene Family Major Minor Isolation method Objective doi**  **Character Character** |
| --- |
| | LOC_Os08g01090 | MF | IDEF1 | ABI3VP1 | MT | Shoot seedling | Others | Germination rate. Seedling growth. | 10.1111/j.1365-313X.2011.04772.x | | --- | --- | --- | --- | --- | --- | --- | --- | --- | | LOC_Os04g52090 | MF | OsAP2-39 | AP2-EREBP | MT | Dwarf | Overexpression | Dwarfism. Fertility. Drought tolerance. | 10.1371/journal.pgen.1001098 | | LOC_Os07g47330 |  | bfl1 | AP2-EREBP | MT | Panicle flower | Mutant | Floral meristem formation. | 10.1186/1471-2229-3-6 | | LOC_Os06g40150 | MF | OsWR2 | AP2-EREBP | MT | Dwarf | Overexpression | dwarfism with enhanced tillers | 10.1007/s11105-013-0687-8 | | LOC_Os10g25170 |  | EBL1 | AP2-EREBP | MT | Dwarf | Knockdown | Internode elongation. | 10.1007/s10529-010-0405-7 | | LOC_Os05g41760 |  | MSF1 | AP2-EREBP | MT | Panicle flower | Mutant | Spikelet determinacy. Floral organ development. | 10.1104/pp.113.216044 | | LOC_Os09g28440 | MF | OsEATB | AP2-EREBP | MT | Culm leaf | Overexpression | Internode elongation. Panicle branching. Tillering. Salinity tolerance. | 10.1104/pp.111.179945 | | LOC_Os04g55560 |  | shat1 | AP2-EREBP | MT | Panicle flower | Mutant | Seed shattering. Floral organ identity. | 10.1105/tpc.111.094383 | | LOC_Os05g03040 | MF | rsr1 | AP2-EREBP | MT | Seed | Mutant | Seed amylose content. Grain size. | 10.1104/pp.110.159517 | | LOC_Os07g13170 |  | snb | AP2-EREBP | MT | Panicle flower | Mutant | Floral meristem formation. | 10.1111/j.1365-313X.2006.02941.x | | LOC_Os03g60430 |  | OsIDS1 | AP2-EREBP | MT | Panicle flower | Mutant | Control of inflorescence architecture and the establishment of floral meristems. | 10.1111/j.1365-313X.2011.04804.x | | LOC_Os05g32270 | MF | smos1 | AP2-EREBP | MT | Others | Mutant | organ size control, small organs and overall plant architecture by increasing cell number and reduced cell size | 10.1093/pcp/pcu023 | | LOC_Os07g03250 |  | crl5 | AP2-EREBP | MT | Root | Mutant | Crown root development. | 10.1111/j.1365-313X.2011.04610.x | | LOC_Os11g32110 |  | osarf1 | ARF | MT | Dwarf | Mutant | Root length. Shoot length. Brassinosteroid sensitivity. | 10.1007/s11103-009-9474-1 | | LOC_Os04g57610 | MF | OsARF12 | ARF | MT | Root | Mutant | Root elongation. Fe uptake. | 10.1111/j.1469-8137.2011.03910.x | | LOC_Os06g09660 | MF | OsARF16 | ARF | MT | Root | Mutant | Auxin response in roots. Phosphate starvation tolerance. | 10.1111/pce.12001 | | LOC_Os03g43400 |  | OsIAA11 | AUX_IAA | MT | Root | Mutant | Lateral root development. | 10.1093/mp/ssr074 | | LOC_Os06g39590 |  | OsIAA23 | AUX_IAA | MT | Root | Mutant | Root development. Quiescent center identity. Auxin sensitivity. | 10.1111/j.1365-313X.2011.04698.x | | LOC_Os01g08320 |  | OsIAA1 | AUX_IAA | MT | Culm leaf | Overexpression | Dwarfism. Leaf angle. Root length. | 10.1007/s11103-009-9474-1 | | LOC_Os12g40900 |  | OsIAA3 | AUX_IAA | MT | Culm leaf | Others | Crown root development. Gravitropism. Leaf blade length. Leaf formation. | 10.1111/j.1365-313X.2006.02693.x | | LOC_Os07g39220 |  | OsBZR1 | BES1 | MT | Culm leaf | Knockdown | Dwarfism. Leaf angle. Brassinosteroid sensitivity. | 10.1073/pnas.0706386104 | | LOC_Os01g18870 |  | bHLH142 | bHLH | MT | Panicle flower | Knockdown | anther size, color (yellowish white), anther development | 10.1105/tpc.114.126292 | | LOC_Os04g28280 |  | An-1 | bHLH | MT | Seed | Natural variation | Awn formation. Grain length. Grain number. | 10.1105/tpc.113.113589 | | LOC_Os01g61480 |  | lax | bHLH | MT | Culm leaf | Mutant | Lateral organ development. Axillary meristem formation. | 10.1073/pnas.1932414100 | | LOC_Os03g56950 |  | OsPIL1 | bHLH | MT | Dwarf | Knockdown Overexpression | Internode elogation. | 10.1073/pnas.1207324109 | | LOC_Os04g23550 |  | RERJ1 | bHLH | MT | Dwarf | Knockdown Overexpression | Dwarfism. JA sensitivity during seedling stage. | 10.1016/j.bbrc.2004.10.126 | | LOC_Os06g12210 |  | bu1 | bHLH | MT | Culm leaf | Overexpression | Leaf angle. Grain size. Brassinosteroid sensitivity. | 10.1104/pp.109.140806 | | LOC_Os06g10880 | MF | Osabf2 | bZIP | MT | Shoot seedling | Mutant | Drought and salinity tolerance. ABA sensitivity during germination and seedling stage | 10.1016/j.jplph.2010.05.008 | | LOC_Os06g50600 |  | OsFD2 | bZIP | MT | Culm leaf | Overexpression | Plastochron. Leaf development. | 10.1093/pcp/pct005 | | LOC_Os07g08420 | MF | OsbZIP58 | bZIP | MT | Seed | Mutant | Seed development. Seed starch content. | 10.1093/jxb/ert187 | | LOC_Os01g15900 | MF | Rdd1 | C2C2-Dof | MT | Seed | Knockdown Overexpression | Grain length and width. 1000-grain weight. Flowering time. | 10.1111/j.1365-3040.2009.01954.x | | LOC_Os05g50270 | MF | nl1 | C2C2-GATA | MT | Panicle flower | Mutant | Floral organ identity. Flowering time. Growth retardation. | 10.1038/cr.2009.36 | | LOC_Os02g12790 | MF | Cga1 | C2C2-GATA | MT | Dwarf | Knockdown Overexpression | Dwarfism. Tillering. Chlorophyll content. Grain filling rate. | 10.1104/pp.113.217265 | | LOC_Os07g06620 |  | YAB1 | C2C2-YABBY | MT | Dwarf | Overexpression | Dwarfism. Regulation of gibberellin level. | 10.1104/pp.107.096586 | | LOC_Os03g44710 |  | OsSh1 | C2C2-YABBY | MT | Panicle flower | Mutant | Seed shattering. | 10.1038/ng.2281 | | LOC_Os04g45330 |  | YAB3 | C2C2-YABBY | MT | Culm leaf | Knockdown | Ligule and auricle formation. Leaf morphology. | 10.1104/pp.107.095737 | | LOC_Os03g11600 | MF | dl | C2C2-YABBY | MT | Culm leaf | Mutant | Drooping leaf.Midrib development. Carpel identity. | 10.1105/tpc.018044 | | LOC_Os03g13400 |  | LPA1 | C2H2 | MT | Culm leaf | Mutant | Tiller angle. Leaf angle. Shoot gravitropism. | 10.1104/pp.112.208496 | | LOC_Os04g47860 | MF | OsIDD10 | C2H2 | MT | Root | Mutant | Ammonium dependent root development. | 10.1111/nph.12075 | | LOC_Os10g28330 | MF | ehd2 | C2H2 | MT | Panicle flower | Mutant | Flowering time independent of day length. Panicle size. | 10.1104/pp.108.125542 | | LOC_Os06g49080 |  | OsLIC | C3H | MT | Culm leaf | Knockdown | Dwarfism. Leaf angle. Tillering. | 10.1371/journal.pone.0003521 | | LOC_Os02g49370 |  | OsHAP3E | CCAAT | MT | Culm leaf | Overexpression | Dwarfism. Leaf angle. Floral organ formation | 10.1016/j.plantsci.2011.04.009 | | LOC_Os03g20790 |  | OsEIL1 | EIL | MT | Dwarf | Overexpression | Dwarfism. Root length. Ethylene sensitivity. | 10.1007/s11103-005-6184-1 | | LOC_Os09g23200 | MF | sll1 | G2-like | MT | Culm leaf | Mutant | Leaf rolling. Chlorophyll content. | 10.1105/tpc.108.061457 | | LOC_Os09g31310 |  | gna1 | GNAT | MT | Root | Mutant | Root length. | 10.1104/pp.104.058248 | | LOC_Os03g15680 | MF | NSP2 | GRAS | MT | Culm leaf | Knockdown | Striga resistance. Regulation of strigolactone biosynthesis. | 10.1105/tpc.111.089771 | | LOC_Os03g51330 |  | OsGRAS19 | GRAS | MT | Dwarf | Knockdown Overexpression | Dwarfism. Leaf angle. Brassinolide sensitivity. | 10.1093/mp/sst027 | | LOC_Os06g40780 |  | moc1 | GRAS | MT | Culm leaf | Mutant | Axillary meristemo formation. Tillering. | 10.1038/nature01518 | | LOC_Os03g29480 | MF | NSP1 | GRAS | MT | Culm leaf | Knockdown | Striga resistance. Regulation of strigolactone biosynthesis. | 10.1105/tpc.111.089771 | | LOC_Os03g49990 |  | slr1 | GRAS | MT | Dwarf | Mutant | Cell division and expansion. Gibberellin sensitivity. | 10.1105/tpc.13.5.999 | | LOC_Os01g45860 |  | SLRL1 | GRAS | MT | Dwarf | Overexpression | Dwarfism. Gibberellin sensitivity. | 10.1111/j.1365-313X.2005.02562.x | | LOC_Os06g03710 |  | d62/dlt | GRAS | MT | Dwarf | Mutant | Dwarfism. Regulation of gibberellin biosynthesis. | 10.1007/s00425-010-1263-1 | | LOC_Os03g51970 |  | OsGRF6 | GRF | MT | Panicle flower | Knockdown | open husk, long sterile lemma | 10.1104/pp.114.235564 | | LOC_Os09g29460 |  | Oshox4 | HB | MT | Dwarf | Overexpression | Dwarfism. Regulation of gibberellin level. | 10.1007/s11103-007-9270-8 | | LOC_Os06g12400 |  | HOX1a | HB | MT | Dwarf | Overexpression | Elongation of uppermost internode. Gibberellin sensitivity. | 10.1111/j.1744-7909.2011.01075.x | | LOC_Os03g56110 |  | OSH43 | HB | MT | Culm leaf | Overexpression | Ligule formation. | 10.1006/dbio.2000.9624 | | LOC_Os05g03884 |  | OSH71 | HB | MT | Culm leaf | Overexpression | Shoot organization. Leaf morphology. | 10.1006/dbio.2000.9624 | | LOC_Os01g19694 |  | OSH6 | HB | MT | Culm leaf | Mutant | Bract differentiation especially at the basal nodes of panicles. | 10.1007/s00425-007-0576-1 | | LOC_Os07g03770 |  | OSH15 | HB | MT | Culm leaf | Overexpression | Shoot organization. Leaf morphology. | 10.1006/dbio.2000.9624 | | LOC_Os03g51690 |  | OSH1 | HB | MT | Culm leaf | Overexpression | Knotted leaf. | 10.1105/tpc.5.9.1039 | | LOC_Os01g63510 |  | QHB | HB | MT | Root | Overexpression | Crown root formation. Shoot formation | 10.1046/j.1365-313X.2003.01816.x | | LOC_Os05g02730 |  | dep | HB | MT | Seed | Natural variation | Trichome formation. | 10.1186/1939-8433-5-28 | | LOC_Os11g01130 |  | WOX3 | HB | MT | Culm leaf | Overexpression | Ligule and auricle formation. | 10.1104/pp.107.095737 | | LOC_Os12g01120 |  | WOX3 | HB | MT | Culm leaf | Overexpression | Ligule and auricle formation. | 10.1104/pp.107.095737 | | LOC_Os07g48560 |  | wox11 | HB | MT | Root | Mutant | Crown root formation and development. Growth retardation | 10.1105/tpc.108.061655 | | LOC_Os01g47710 |  | DWT1 | HB | MT | Culm leaf | Mutant | internode elongation, cell division and elongation, tiller growth | 10.1371/journal.pgen.1004154 | | LOC_Os04g55590 |  | WOX4 | HB | MT | Shoot seedling | Overexpression | Maintainance of shoot apical meristem. | 10.1105/tpc.112.103432 | | LOC_Os02g45250 |  | oul1 | HB | MT | Culm leaf | Mutant | Leaf rolling. Bulliform cell size and number. | 10.1104/pp.111.176016 | | LOC_Os01g62920 |  | qSH1 | HB | MT | Panicle flower | Natural variation | Seed shattering. | 10.1126/science.1126410 | | LOC_Os05g10770 |  | JMJ703 | Jumonji | MT | Dwarf | Mutant | Dwarfism. Panicle size. Grain size. Leaf angle. | 10.1073/pnas.1217020110 | | LOC_Os10g42690 |  | jmj6 | Jumonji | MT | Panicle flower | Mutant | Number and morphology of floral organ. | 10.1073/pnas.0805901105 | | LOC_Os04g51000 | MF | RFL | LFY | MT | Culm leaf | Knockdown Overexpression | Flowering time. Tiller growth. Panicle branching. | 10.1073/pnas.0709059105 | | LOC_Os02g57490 |  | dh1 | LOB | MT | Panicle flower | Mutant | Glume formation. | 10.1007/s11103-007-9283-3 | | LOC_Os03g05510 |  | arl1 | LOB | MT | Root | Mutant | Crown root formation. Auxin sensitivity. | 10.1111/j.1365-313X.2005.02434.x | | LOC_Os01g52680 |  | CFO1 | MADS | MT | Panicle flower | Mutant | Floral organ formation. | 10.1104/pp.112.200980 | | LOC_Os02g07430 | MF | MADS29 | MADS | MT | Seed | Mutant | Seed amylose content. Grain filling. | 10.1105/tpc.111.094854 | | LOC_Os06g49840 |  | spw1 | MADS | MT | Panicle flower | Mutant | Floral organ formation. | 10.1242/dev.00294 | | LOC_Os05g34940 |  | OsMADS4 | MADS | MT | Panicle flower | Knockdown | Floral organ identity. | 10.1023/A:1006051911291 | | LOC_Os07g01820 | MF | MADS15 | MADS | MT | Panicle flower | Knockdown | Floral organ formation. | 10.1105/tpc.112.097105 | | LOC_Os03g54160 |  | MADS14 | MADS | MT | Panicle flower | Knockdown | Floral organ formation. | 10.1105/tpc.112.097105 | | LOC_Os09g32948 |  | OsMADS8 | MADS | MT | Panicle flower | Knockdown | Floral organ formation. | 10.1111/j.1365-313X.2009.04101.x | | LOC_Os08g41950 |  | OsMADS7 | MADS | MT | Panicle flower | Knockdown | Floral organ formation. | 10.1111/j.1365-313X.2009.04101.x | | LOC_Os03g54170 |  | pap2 | MADS | MT | Panicle flower | Mutant | Floral organ formation. Spikelet meristem identity. | 10.1093/pcp/pcp166 | | LOC_Os06g06750 |  | OsMADS5 | MADS | MT | Panicle flower | Knockdown | Floral organ formation. | 10.1111/j.1365-313X.2009.04101.x | | LOC_Os03g11614 | MF | OsMADS1 | MADS | MT | Panicle flower | Knockdown | Floral organ formation. | 10.1111/j.1365-313X.2009.04101.x | | LOC_Os02g45770 | MF | mfo1 | MADS | MT | Panicle flower | Mutant | Floral organ identity. | 10.1105/tpc.109.068742 | | LOC_Os12g10540 |  | OsMADS13 | MADS | MT | Panicle flower | Mutant | Ovule identity. | 10.1111/j.1365-313X.2007.03272.x | | LOC_Os05g11414 |  | mads58 | MADS | MT | Panicle flower | Mutant | Floral organ identity. | 10.1105/tpc.111.087262 | | LOC_Os01g10504 | MF | OsMADS3 | MADS | MT | Panicle flower | Knockdown | Floral organ identity. | 10.1023/A:1006051911291 | | LOC_Os03g08754 |  | OsMDP1 | MADS | MT | Culm leaf | Knockdown | Primary root elongation. Coleoptile elongation. Leaf angle. Brassinosteroid sensitivity. | 10.1111/j.1365-313X.2006.02804.x | | LOC_Os06g11330 |  | OsMADS55 | MADS | MT | Culm leaf | Knockdown Overexpression | Dwarfism. Leaf angle. Brassinosteroid sensitivity. | 10.1111/j.1365-313X.2008.03406.x | | LOC_Os02g52340 |  | OsMADS22 | MADS | MT | Culm leaf | Knockdown Overexpression | Dwarfism. Leaf angle. Brassinosteroid sensitivity. | 10.1111/j.1365-313X.2008.03406.x | | LOC_Os02g49840 |  | OsMADS57 | MADS | MT | Culm leaf | Knockdown Overexpression | Tillering. | 10.1038/ncomms2542 | | LOC_Os02g40530 |  | OsMPS | MYB | MT | Dwarf | Knockdown Overexpression | Grain size. Total biomass. | 10.1111/tpj.12286 | | LOC_Os11g35390 | MF | OsMYB4P | MYB | MT | Root | Overexpression | regulation of Pi-dependent root architecture by facilitating Pi acquisition under Pi deficient conditions | 10.1016/j.plaphy.2014.02.024 | | LOC_Os05g04820 | MF | OsMYB2P-1 1 | MYB | MT | Root | Knockdown Overexpression | Phosphate starvation tolerance. Phosphate responsive root growth. | 10.1104/pp.112.194217 | | LOC_Os08g05520 |  | OsMYB103L | MYB | MT | Culm leaf | Knockdown Overexpression | leaf rolling and mechanical strength through cellulose content (regulation of cellulose synthase (CESA) gene expression) | 10.1186/1471-2229-14-158 | | LOC_Os12g41680 | MF | OMTN3 | NAC | MT | Culm leaf | Knockdown Overexpression | leaf rolling, wilting during drought stress | 10.1093/jxb/eru072 | | LOC_Os08g10080 | MF | OMTN6 | NAC | MT | Culm leaf | Knockdown Overexpression | leaf rolling, wilting during drought stress | 10.1093/jxb/eru072 | | LOC_Os06g46270 | MF | OMTN4 | NAC | MT | Culm leaf | Knockdown Overexpression | leaf rolling, wilting during drought stress | 10.1093/jxb/eru072 | | LOC_Os04g38720 | MF | Ostil1 | NAC | MT | Dwarf | Knockdown | Dwarfism. Tillering. Tiller angle. | 10.1111/j.1469-8137.2007.02177.x | | LOC_Os01g66120 | MF | OsNAC6 | NAC | MT | Root | Knockdown Overexpression | Seedling root growth | 10.1111/j.1365-313X.2009.03908.x | | LOC_Os04g36070 |  | OsRR1 | Orphans | MT | Root | Overexpression | Crown root development. | 10.1111/j.1365-313X.2011.04610.x | | LOC_Os04g57720 |  | OsRR6 | Orphans | MT | Dwarf | Overexpression | Dwarfism. Root development. Cytokinin metabolism. | 10.1093/pcp/pcm022 | | LOC_Os07g31450 | MF | chr729 | PHD | MT | Dwarf | Mutant | Dwarfism. Narrowed leaf. Panicle branching. Chlorophyll content. Chromatin modification. | 10.1073/pnas.1203148109 | | LOC_Os04g56170 |  | OsLG1 | SBP | MT | Panicle flower | Natural variation | Closed panicle. | 10.1038/ng.2567 | | LOC_Os08g41940 |  | qGW8 | SBP | MT | Seed | Natural variation | Grain size. | 10.1038/ng.2327 | | LOC_Os08g39890 | MF | WFP | SBP | MT | Culm leaf | Natural variation | Panicle branching. Tillering. | 10.1038/ng.592 | | LOC_Os01g70220 |  | SDG714 | SET | MT | Culm leaf | Knockdown | Glabrous. DNA methylation. | 10.1105/tpc.106.048124 | | LOC_Os05g41172 | MF | SDG728 | SET | MT | Seed | Knockdown Overexpression | Grain size. Chromatin modification. | 10.1093/mp/ssq030 | | LOC_Os09g27060 | MF | OsDDM1a | SNF2 | MT | Dwarf | Knockdown | Dwarfism, DNA methylation | 10.1007/s00438-012-0717-5 | | LOC_Os03g51230 | MF | OsDDM1b | SNF2 | MT | Dwarf | Knockdown | Dwarfism, DNA methylation | 10.1007/s00438-012-0717-5 | | LOC_Os03g49880 |  | fc1/OsTB1 | TCP | MT | Culm leaf | Mutant | Tiller growth. | 10.1046/j.1365-313X.2003.01648.x | | LOC_Os09g24480 |  | rep1 | TCP | MT | Panicle flower | Mutant | Floral organ formation. | 10.1104/pp.108.128231 | | LOC_Os03g08330 |  | TIFY11b | Tify | MT | Dwarf | Overexpression | Grain size. Plant height | 10.1271/bbb.120545 | | LOC_Os02g26430 | MF | OsWRKY42 | WRKY | MT | Culm leaf | Overexpression | promotion of leaf senescence through ROS accumulation, plant death | 10.14348/molcells.2014.0128 | | LOC_Os07g39480 |  | oswrky78 | WRKY | MT | Dwarf | Mutant | Dwarfism. Grain size. Cell elongation. | 10.1007/s00425-011-1423-y | | LOC_Os01g43650 | MF | DIF1 | WRKY | MT | Dwarf | Knockdown Overexpression | plant height through control of cell size | 10.1371/journal.pone.0102529 | | LOC_Os11g02520 | MF | OsWRKY89 | WRKY | MT | Dwarf | Overexpression | Blast, UV and white-backed planthopper resistance. Internode elongation. Growth retardation at the early stage. | 10.1007/s11103-007-9244-x | | LOC_Os03g20550 | MF | OsWRKY31 | WRKY | MT | Root | Knockdown Overexpression | Resistance to Magnaporthe grisea. Lateral root growth. | 10.1038/cr.2007.104 | | LOC_Os09g29130 |  | OsZHD1 | zf-HD | MT | Culm leaf | Knockdown Overexpression | abaxially curled and drooping leaf-dominant, leaf rolling, formation and distribution of bulliform cells | 10.1007/s00425-013-2009-7 | | LOC_Os04g47080 |  | OSB1 | bHLH | Others | Others | Natural variation | Purple leaf. Anthocyanin biosynthesis. | 10.1093/pcp/pce128 | | LOC_Os04g47059 |  | OSB2 | bHLH | Others | Others | Natural variation | Purple leaf. Anthocyanin biosynthesis. | 10.1093/pcp/pce128 | | LOC_Os06g10880 | MF | abl1 | bZIP | Others | Others | Mutant | Sensitivity to IAA and ABA. | 10.1104/pp.111.173427 | | LOC_Os05g45410 | MF | spl7 | HSF | Others | Others | Mutant | Lesion mimic. | 10.1073/pnas.112209199 | | LOC_Os08g02070 |  | OsMADS26 | MADS | Others | Others | Overexpression | Pleiotropic developmental defect. | 10.1104/pp.107.114256 | | LOC_Os02g57270 |  | rtbp1 | MYB-related | Others | Others | Mutant | Germ cell development. Pleiotropic developmental defect. Regulation of telomere length. | 10.1105/tpc.107.051953 | | LOC_Os04g08740 | MF | etr2 | Orphans | Others | Others | Mutant | Flowering time. Ethylene sensitivity. Stem starch content. | 10.1105/tpc.108.065391 | | LOC_Os02g50480 |  | OsHk6 | Orphans | Others | Others | Overexpression | Cytokinin-dependent green pigmentation in calli. | 10.1093/pcp/pcs079 | | LOC_Os05g41172 | MF | SDG728 | SET | Others | Others | Knockdown Overexpression | Grain size. Chromatin modification. | 10.1093/mp/ssq030 | | LOC_Os09g27060 | MF | OsDDM1a | SNF2 | Others | Others | Knockdown | Dwarfism, DNA methylation | 10.1007/s00438-012-0717-5 | | LOC_Os03g51230 | MF | OsDDM1b | SNF2 | Others | Others | Knockdown | Dwarfism, DNA methylation | 10.1007/s00438-012-0717-5 | | LOC_Os01g51610 |  | OsLFL | ABI3VP1 | PT | Flowering | Mutant | Flowering time. | 10.1016/j.jplph.2007.07.010 | | LOC_Os08g01090 | MF | IDEF1 | ABI3VP1 | PT | Germination dormancy | Others | Germination rate. Seedling growth. | 10.1111/j.1365-313X.2011.04772.x | | LOC_Os02g43970 | MF | ARAG1 | AP2-EREBP | PT | Germination dormancy | Knockdown Overexpression | Drought tolerance during seedling stage. ABA sensitivity during germination. | 10.1093/aob/mcp303 | | LOC_Os04g52090 | MF | OsAP2-39 | AP2-EREBP | PT | Sterility | Overexpression | Dwarfism. Fertility. Drought tolerance. | 10.1371/journal.pgen.1001098 | | LOC_Os06g40150 | MF | OsWR2 | AP2-EREBP | PT | Seed | Overexpression | seed yield reduced | 10.1007/s11105-013-0687-8 | | LOC_Os05g03040 | MF | rsr1 | AP2-EREBP | PT | Eating quality | Mutant | Seed amylose content. Grain size. | 10.1104/pp.110.159517 | | LOC_Os05g32270 | MF | smos1 | AP2-EREBP | PT | Others | Mutant | functions in auxin signaling, involvement in microtubule organization by directly regulating the expression of OsPHI-1 | 10.1093/pcp/pcu023 | | LOC_Os07g36460 |  | OsUDT1 | bHLH | PT | Panicle flower | Knockdown | stamen development, posttranscriptional splicing target of the EJC subunit | 10.1104/pp.114.237958 | | LOC_Os01g64000 | MF | OsABI5 | bZIP | PT | Sterility | Knockdown Overexpression | Salinity tolerance. Fertility. | 10.1007/s11103-008-9298-4 | | LOC_Os06g10880 | MF | Osabf2 | bZIP | PT | Germination dormancy | Mutant | Drought and salinity tolerance. ABA sensitivity during germination and seedling stage | 10.1016/j.jplph.2010.05.008 | | LOC_Os09g36910 |  | OsFD1 | bZIP | PT | Flowering | Knockdown | Flowering time. | 10.1038/nature10272 | | LOC_Os07g08420 | MF | RISBZ1 | bZIP | PT | Eating quality | Knockdown | Seed storage protein and starch content. | 10.1111/j.1365-313X.2009.03925.x | | LOC_Os02g49230 |  | DTH2 | C2C2-CO-like | PT | Flowering | Natural variation | Flowering time under long day condition. Seed maturity. | 10.1073/pnas.1213962110 | | LOC_Os02g39710 |  | OsCOL4 | C2C2-CO-like | PT | Flowering | Mutant | Flowering time independent of day length. | 10.1111/j.1365-313X.2010.04226.x | | LOC_Os06g16370 |  | Hd1 | C2C2-CO-like | PT | Flowering | Natural variation | Flowering time. | 10.1105/tpc.12.12.2473 | | LOC_Os09g06464 |  | OsCO3 | C2C2-CO-like | PT | Flowering | Overexpression | Flowering time under short day condition. | 10.1007/s00425-008-0742-0 | | LOC_Os03g07360 |  | OsDof12 | C2C2-Dof | PT | Flowering | Overexpression | Flowering time under long day condition. | 10.1007/s00425-009-0893-7 | | LOC_Os01g15900 | MF | Rdd1 | C2C2-Dof | PT | Flowering | Knockdown Overexpression | Grain length and width. 1000-grain weight. Flowering time. | 10.1111/j.1365-3040.2009.01954.x | | LOC_Os02g15350 |  | RPBF | C2C2-Dof | PT | Eating quality | Knockdown | Seed storage protein and starch content. | 10.1111/j.1365-313X.2009.03925.x | | LOC_Os05g50270 | MF | nl1 | C2C2-GATA | PT | Flowering | Mutant | Floral organ identity. Flowering time. Growth retardation. | 10.1038/cr.2009.36 | | LOC_Os02g12790 | MF | Cga1 | C2C2-GATA | PT | Source activity | Knockdown Overexpression | Dwarfism. Tillering. Chlorophyll content. Grain filling rate. | 10.1104/pp.113.217265 | | LOC_Os05g51830 | MF | OsHDT1 | C2H2 | PT | Flowering | Overexpression | Flowering time in hybrid. Chromatin modification. | 10.1371/journal.pone.0021789 | | LOC_Os10g28330 | MF | ehd2 | C2H2 | PT | Flowering | Mutant | Flowering time independent of day length. Panicle size. | 10.1104/pp.108.125542 | | LOC_Os01g09620 |  | OsDOS | C3H | PT | Source activity | Knockdown Overexpression | Leaf senescence. JA sensitivity. | 10.1104/pp.106.082941 | | LOC_Os03g02160 |  | Ehd4 | C3H | PT | Flowering | Mutant | Flowering time. Grain number. | 10.1371/journal.pgen.1003281 | | LOC_Os08g07740 |  | DTH8 | CCAAT | PT | Flowering | Natural variation | Flowering time under long day condition. | 10.1104/pp.110.156943 | | LOC_Os05g49780 |  | OsHAP3C | CCAAT | PT | Source activity | Knockdown | Chloroplast development. | 10.1046/j.1365-313X.2003.01897.x | | LOC_Os01g61810 |  | OsHAP3A | CCAAT | PT | Source activity | Knockdown | Chloroplast development. | 10.1046/j.1365-313X.2003.01897.x | | LOC_Os05g38820 |  | OsHAP3B | CCAAT | PT | Source activity | Knockdown | Chloroplast development. | 10.1046/j.1365-313X.2003.01897.x | | LOC_Os10g32600 |  | Ehd1 | G2-like | PT | Flowering | Natural variation | Flowering time independent of day length. | 10.1101/gad.1189604 | | LOC_Os03g45194 |  | nol1 | G2-like | PT | Source activity | Mutant | Leaf senescence. Chlorophyll b and light-harvesting complex II degradation. | 10.1111/j.1365-313X.2008.03670.x | | LOC_Os07g25710 | MF | OsPHR2 | G2-like | PT | Others | Overexpression | phosphate uptake efficiency through oxygen supply | 10.1007/s10265-014-0628-0 | | LOC_Os09g23200 | MF | sll1 | G2-like | PT | Source activity | Mutant | Leaf rolling. Chlorophyll content. | 10.1105/tpc.108.061457 | | LOC_Os06g24070 |  | OsGLK1 | G2-like | PT | Source activity | Overexpression | Chloroplast development. | 10.1093/pcp/pcp138 | | LOC_Os02g43330 | MF | OsSLI1 | HB | PT | Panicle flower | Others | panicle development | 10.1155/2014/809353 | | LOC_Os12g41860 |  | OsHox33 | HB | PT | Source activity | Knockdown | Leaf senescence. Chloroplast degradation. | 10.1007/s11427-013-4565-2 | | LOC_Os03g05680 |  | Se14 | Jumonji | PT | Flowering | Mutant | delay flowering through the repression of RFT1 by red-light signal mediated H3K4me3 demethylation under long day-length conditions | 10.1371/journal.pone.0096064 | | LOC_Os04g51000 | MF | RFL | LFY | PT | Flowering | Knockdown Overexpression | Flowering time. Tiller growth. Panicle branching. | 10.1073/pnas.0709059105 | | LOC_Os02g07430 | MF | MADS29 | MADS | PT | Eating quality | Mutant | Seed amylose content. Grain filling. | 10.1105/tpc.111.094854 | | LOC_Os01g69850 |  | OsMADS51 | MADS | PT | Flowering | Mutant | Flowering time under short day condition. | 10.1104/pp.107.103291 | | LOC_Os07g01820 | MF | OsMADS15 | MADS | PT | Flowering | Overexpression | Floral organ formation. Dwarfism. Tiller angle. Flowering time. | 10.1007/s11105-012-0468-9 | | LOC_Os07g41370 |  | OsMADS18 | MADS | PT | Flowering | Overexpression | Flowering time. | 10.1104/pp.104.045039 | | LOC_Os03g11614 | MF | lhs1 | MADS | PT | Flowering | Mutant | Floral organ identity. Flowering time. | 10.1105/tpc.12.6.871 | | LOC_Os02g45770 | MF | osmads6 | MADS | PT | Eating quality | Mutant | Floral organ identity. Seed starch filling. | 10.1111/j.1365-313X.2010.04354.x | | LOC_Os01g10504 | MF | mads3 | MADS | PT | Sterility | Mutant | Late anther development. | 10.1105/tpc.110.074369 | | LOC_Os10g39130 |  | OsMADS56 | MADS | PT | Flowering | Overexpression | Flowering time under long day condition. | 10.1111/j.1365-3040.2009.02008.x | | LOC_Os01g59660 |  | gamyb | MYB | PT | Sterility | Mutant | Pollen development. | 10.1105/tpc.017327 | | LOC_Os01g16810 |  | CSA | MYB | PT | Sterility | Mutant | Photoperiod-sensitive male sterility | 10.1073/pnas.1213041110 | | LOC_Os06g08290 |  | aid1 | MYB-related | PT | Sterility | Mutant | Pollen development. Anther dehiscence. | 10.1104/pp.104.041459 | | LOC_Os12g41680 | MF | OMTN3 | NAC | PT | Panicle flower | Overexpression | low spikelet fertility | 10.1093/jxb/eru072 | | LOC_Os08g10080 | MF | OMTN6 | NAC | PT | Panicle flower | Overexpression | low spikelet fertility | 10.1093/jxb/eru072 | | LOC_Os06g46270 | MF | OMTN4 | NAC | PT | Panicle flower | Overexpression | low spikelet fertility | 10.1093/jxb/eru072 | | LOC_Os04g38720 | MF | OMTN2 | NAC | PT | Panicle flower | Overexpression | low spikelet fertility | 10.1093/jxb/eru072 | | LOC_Os03g21060 |  | OsNAP | NAC | PT | Source activity | Overexpression | Leaf senescence at the grain-filling stage. | 10.1186/1471-2229-13-132 | | LOC_Os11g08210 | MF | OsNAC5 | NAC | PT | Germination dormancy | Knockdown Overexpression | Cold tolerance. Sensitivity to salt, mannitol and ABA during germination. | 10.1007/s00425-011-1403-2 | | LOC_Os07g15770 |  | Ghd7 | Orphans | PT | Flowering | Natural variation | Flowering time under long day condition. | 10.1038/ng.143 | | LOC_Os04g08740 | MF | etr2 | Orphans | PT | Flowering | Mutant | Flowering time. Ethylene sensitivity. Stem starch content. | 10.1105/tpc.108.065391 | | LOC_Os09g27620 |  | PTC1 | PHD | PT | Sterility | Mutant | Tapetal cell death. | 10.1104/pp.111.175760 | | LOC_Os05g03430 |  | siz1 | PHD | PT | Sterility | Mutant | Anther dehiscence. | 10.1111/j.1469-8137.2010.03538.x | | LOC_Os08g01420 |  | ehd3 | PHD | PT | Flowering | Mutant | Flowering time under long day condition. | 10.1111/j.1365-313X.2011.04517.x | | LOC_Os07g31450 | MF | OsCHR4 | PHD | PT | Source activity | Mutant | Chloroplast development in adaxial mesophyll | 10.1007/s00425-012-1667-1 | | LOC_Os09g04890 |  | OsTrx1 | PHD | PT | Flowering | Knockdown | flowering time | 10.1104/pp.113.228049 | | LOC_Os07g49460 |  | OsPRR37 | Pseudo_ARR-B | PT | Flowering | Natural variation | Flowering time. | 10.1093/mp/sst088 | | LOC_Os09g13740 |  | SDG724 | SET | PT | Flowering | Mutant | Flowering time independent of day length. | 10.1105/tpc.112.101436 | | LOC_Os02g26430 | MF | OsWRKY42 | WRKY | PT | Culm leaf | Overexpression | directly regulate ROS accumulation, chlorophyll contents, repress OsMT1d expression | 10.14348/molcells.2014.0128 | | LOC_Os01g43650 | MF | DIF1 | WRKY | PT | Flowering | Knockdown Overexpression | flowering time | 10.1371/journal.pone.0102529 | | LOC_Os08g01090 | MF | IDEF1 | ABI3VP1 | RT | Other soil stress tolerance | Overexpression | Fe deficiency tolerance. | 10.1073/pnas.0707010104 | | LOC_Os08g35240 |  | OsDERF1 | AP2-EREBP | RT | Drought tolerance | Knockdown Overexpression | Drought tolerance. Regulation of ethylene biosynthesis. | 10.1371/journal.pone.0025216 | | LOC_Os05g34730 |  | SERF1 | AP2-EREBP | RT | Salinity tolerance | Mutant | Salinity tolerance. | 10.1105/tpc.113.113068 | | LOC_Os06g03670 |  | OsDREB1C | AP2-EREBP | RT | Cold tolerance | Overexpression | Cold, drought and salinity tolerance. | 10.1093/pcp/pci230 | | LOC_Os09g35010 |  | OsDREB1B | AP2-EREBP | RT | Cold tolerance | Overexpression | Cold, drought and salinity tolerance. | 10.1093/pcp/pci230 | | LOC_Os09g35030 |  | OsDREB1A | AP2-EREBP | RT | Cold tolerance | Overexpression | Cold, drought and salinity tolerance. | 10.1093/pcp/pci230 | | LOC_Os08g43200 |  | OsDREB1G | AP2-EREBP | RT | Drought tolerance | Overexpression | Drought tolerance. | 10.1007/s10529-008-9811-5 | | LOC_Os04g48350 |  | OsDREB1E | AP2-EREBP | RT | Drought tolerance | Overexpression | Drought tolerance. | 10.1007/s10529-008-9811-5 | | LOC_Os01g73770 |  | OsDREB1F | AP2-EREBP | RT | Cold tolerance | Overexpression | Cold, drought and salinity tolerance. | 10.1007/s11103-008-9340-6 | | LOC_Os02g43970 | MF | ARAG1 | AP2-EREBP | RT | Drought tolerance | Knockdown Overexpression | Drought tolerance during seedling stage. ABA sensitivity during germination. | 10.1093/aob/mcp303 | | LOC_Os05g27930 |  | OsDREB2B | AP2-EREBP | RT | Drought tolerance | Overexpression | Drought tolerance. | 10.1007/s10529-008-9811-5 | | LOC_Os01g07120 |  | OsDREB2A | AP2-EREBP | RT | Drought tolerance | Overexpression | Drought, salinity and osmotic stress tolerance. | 10.1007/s10529-011-0620-x | | LOC_Os09g20350 |  | OsDREB6 | AP2-EREBP | RT | Other stress resistance | Knockdown Overexpression | osmotic tolerance, cold stress tolerance | 10.1007/s12374-013-0480-0 | | LOC_Os04g52090 | MF | OsAP2-39 | AP2-EREBP | RT | Drought tolerance | Overexpression | Drought tolerance. Regulation of ethylene biosynthesis. | 10.1371/journal.pone.0025216 | | LOC_Os01g58420 |  | OsERF3 | AP2-EREBP | RT | Drought tolerance | Overexpression | Drought tolerance through controlling the ethylene biosynthesis. | 10.1371/journal.pone.0025216 | | LOC_Os06g40150 | MF | OsWR2 | AP2-EREBP | RT | Drought tolerance | Overexpression | drought tolerance through cuticular permeability | 10.1007/s11105-013-0687-8 | | LOC_Os09g28440 | MF | OsEATB | AP2-EREBP | RT | Salinity tolerance | Overexpression | Internode elongation. Panicle branching. Tillering. Salinity tolerance. | 10.1104/pp.111.179945 | | LOC_Os04g57610 | MF | OsARF12 | ARF | RT | Other soil stress tolerance | Mutant | Root elongation. Fe uptake. | 10.1111/j.1469-8137.2011.03910.x | | LOC_Os06g09660 | MF | OsARF16 | ARF | RT | Other soil stress tolerance | Mutant | Auxin response in roots. Phosphate starvation tolerance. | 10.1111/pce.12001 | | LOC_Os01g48060 |  | OsETT2 | ARF | RT | Panicle flower | Knockdown | awn development | 10.1111/tpj/12411 | | LOC_Os03g53020 |  | OsbHLH148 | bHLH | RT | Drought tolerance | Overexpression | Drought tolerance. | 10.1111/j.1365-313X.2010.04477.x | | LOC_Os06g09370 |  | OsPTF1 | bHLH | RT | Other soil stress tolerance | Overexpression | Phosphate starvation tolerance. | 10.1104/pp.105.063115 | | LOC_Os12g32400 |  | OsbHLH133 | bHLH | RT | Other soil stress tolerance | Mutant | Fe translocation. Growth retardation. | 10.1111/j.1365-3040.2012.02569.x | | LOC_Os01g72370 |  | OsIRO2 | bHLH | RT | Other soil stress tolerance | Knockdown Overexpression | Fe uptake under Fe-deficient conditions. Secretion of mugineic acid family phytosiderophores. | 10.1111/j.1365-313X.2007.03149.x | | LOC_Os01g70310 |  | OrbHLH001 | bHLH | RT | Salinity tolerance | Overexpression | Salinity tolerance. Regulation of Na and K homeostasis. | 10.1016/j.jplph.2012.08.019 | | LOC_Os03g26210 |  | OsIRO3 | bHLH | RT | Other soil stress tolerance | Overexpression | Fe homeostasis. Response to Fe deficiency. | 10.1186/1471-2229-10-166 | | LOC_Os04g54474 |  | ostgap1 | bZIP | RT | Other stress resistance | Overexpression | Regulation of production of phytoalexins and allelochemicals. | 10.1074/jbc.M109.036871 | | LOC_Os07g48820 |  | rtGA2.1 | bZIP | RT | Bacterial blight resistance | Knockdown | Resistance to Xanthomonas oryzae pv. oryzae. Growth retardation. | 10.1111/j.1365-313X.2005.02457.x | | LOC_Os09g34060 |  | RF2a | bZIP | RT | Other disease resistance | Overexpression | Rice tungro disease resistance. | 10.1073/pnas.0810303105 | | LOC_Os01g64730 |  | Osabf1 | bZIP | RT | Drought tolerance | Mutant | Drought and salinity tolerance. | 10.1007/s11103-009-9592-9 | | LOC_Os01g64000 | MF | OsABI5 | bZIP | RT | Salinity tolerance | Knockdown Overexpression | Salinity tolerance. Fertility. | 10.1007/s11103-008-9298-4 | | LOC_Os09g28310 |  | OsbZIP72 | bZIP | RT | Drought tolerance | Overexpression | Drought tolerance. ABA sensitivity. | 10.1007/s00425-008-0857-3 | | LOC_Os06g10880 | MF | Osabf2 | bZIP | RT | Drought tolerance | Mutant | Drought and salinity tolerance. ABA sensitivity during germination and seedling stage | 10.1016/j.jplph.2010.05.008 | | LOC_Os02g52780 |  | OsbZIP23 | bZIP | RT | Drought tolerance | Mutant | Drought and salinity tolerance. ABA sensitivity. | 10.1104/pp.108.128199 | | LOC_Os09g13570 |  | OsbZIP71 | bZIP | RT | Drought tolerance | Knockdown Overexpression | Drought and salinity tolerance. | 10.1007/s11103-013-0115-3 | | LOC_Os09g29820 |  | OsTFX1 | bZIP | RT | Bacterial blight resistance | Overexpression | Resistance to Xanthomonas oryzae pv. oryzae. | 10.1073/pnas.0701742104 | | LOC_Os06g45140 |  | OsbZIP52/RISBZ5 | bZIP | RT | Cold tolerance | Overexpression | Cold and drought tolerance. | 10.1007/s00425-011-1564-z | | LOC_Os03g11600 | MF | DL | C2C2-YABBY | RT | Panicle flower | Knockdown | promote awn formation in a non-cell-autonomous manner | 10.1111/tpj/12411 | | LOC_Os05g51830 | MF | HDT701 | C2H2 | RT | Bacterial blight resistance | Knockdown Overexpression | Resistance to Magnaporthe oryzae and Xanthomonas oryzae pv oryzae. | 10.1105/tpc.112.101972 | | LOC_Os12g39400 |  | ZFP252 | C2H2 | RT | Drought tolerance | Overexpression | Drought and salinity tolerance. | 10.1016/j.febslet.2008.02.052 | | LOC_Os03g60560 |  | ZFP182 | C2H2 | RT | Salinity tolerance | Overexpression | Salinity tolerance. | 10.1016/j.bbaexp.2007.02.006 | | LOC_Os01g67970 |  | JMJ705 | C2H2 | RT | Bacterial blight resistance | Mutant | Resistance to Xanthomonas oryzae pv oryzae. | 10.1105/tpc.113.118802 | | LOC_Os12g07280 |  | art1 | C2H2 | RT | Other soil stress tolerance | Mutant | Aluminium tolerance. Root elongation in acidic soil. | 10.1105/tpc.109.070771 | | LOC_Os04g47860 | MF | OsIDD10 | C2H2 | RT | Other soil stress tolerance | Mutant | Ammonium dependent root development. | 10.1111/nph.12075 | | LOC_Os05g10670 |  | OsTZF1 | C3H | RT | Drought tolerance | Knockdown Overexpression | Drought and salinity tolerance. | 10.1104/pp.112.205385 | | LOC_Os01g68860 |  | C3H12 | C3H | RT | Bacterial blight resistance | Mutant | Resistance to Xanthomonas oryzae pv. oryzae. JA level. | 10.1104/pp.111.191379 | | LOC_Os07g30774 |  | oscbt | CAMTA | RT | Bacterial blight resistance | Mutant | Resistance to Xanthomonas oryzae and Magnaporthe grisea. | 10.1007/s10059-009-0081-4 | | LOC_Os07g25710 | MF | OsPHR2 | G2-like | RT | Other soil stress tolerance | Overexpression | Response to phosphate starvation. | 10.1104/pp.107.111443 | | LOC_Os03g15680 | MF | NSP2 | GRAS | RT | Other stress resistance | Knockdown | Striga resistance. Regulation of strigolactone biosynthesis. | 10.1105/tpc.111.089771 | | LOC_Os03g29480 | MF | NSP1 | GRAS | RT | Other stress resistance | Knockdown | Striga resistance. Regulation of strigolactone biosynthesis. | 10.1105/tpc.111.089771 | | LOC_Os04g45810 |  | Oshox22 | HB | RT | Drought tolerance | Mutant | Drought and salinity tolerance. | 10.1007/s11103-012-9967-1 | | LOC_Os02g43330 | MF | OsSLI1 | HB | RT | Other stress resistance | Others | abiotic stress response | 10.1155/2014/809353 | | LOC_Os01g39020 |  | OsHsfA7 | HSF | RT | Drought tolerance | Overexpression | Drought and salinity tolerance. | 10.5483/BMBRep.2013.46.1.090 | | LOC_Os08g43334 |  | OsHsfB2b | HSF | RT | Salinity tolerance | Knockdown Overexpression | Drought and salinity tolerance. | 10.1007/s00299-013-1492-4 | | LOC_Os05g45410 | MF | OsHsfA4a | HSF | RT | Other soil stress tolerance | Mutant | Cadmium tolerance. | 10.1105/tpc.109.066902 | | LOC_Os11g45740 |  | OsMPK5 | MYB | RT | Other disease resistance | Knockdown | Resistance to brown spot pathogen Cochliobolus miyabeanus. | 10.1104/pp.109.152702 | | LOC_Os03g20090 |  | OsMYB2 | MYB | RT | Cold tolerance | Overexpression | Cold, drought and salinity tolerance. ABA sensitivity. | 10.1093/jxb/err431 | | LOC_Os05g48010 |  | OsMYB55 | MYB | RT | Other stress resistance | Overexpression | Growth under high temprature. | 10.1371/journal.pone.0052030 | | LOC_Os11g35390 | MF | OsMYB4P | MYB | RT | Other stress resistance | Overexpression | tolerance to low Pi | 10.1016/j.plaphy.2014.02.024 | | LOC_Os05g04820 | MF | OsMYB2P-1 1 | MYB | RT | Other soil stress tolerance | Knockdown Overexpression | Phosphate starvation tolerance. Phosphate responsive root growth. | 10.1104/pp.112.194217 | | LOC_Os01g62410 |  | OsMYB3R-2 | MYB | RT | Cold tolerance | Overexpression | Cold tolerance. | 10.1104/pp.108.133454 | | LOC_Os10g41200 |  | MYBS3 | MYB-related | RT | Cold tolerance | Overexpression | Cold tolerance. | 10.1104/pp.110.153015 | | LOC_Os02g56600 |  | NAC32 | NAC | RT | Salinity tolerance | Natural variation | regulation of NaCl stress responses | 10.1007/s11738-014-1569-x | | LOC_Os12g41680 | MF | OMTN3 | NAC | RT | Drought tolerance | Overexpression | drought sensitivity | 10.1093/jxb/eru072 | | LOC_Os08g10080 | MF | OMTN6 | NAC | RT | Drought tolerance | Overexpression | drought sensitivity | 10.1093/jxb/eru072 | | LOC_Os06g46270 | MF | OMTN4 | NAC | RT | Drought tolerance | Overexpression | drought sensitivity | 10.1093/jxb/eru072 | | LOC_Os04g38720 | MF | OMTN2 | NAC | RT | Drought tolerance | Overexpression | drought sensitivity | 10.1093/jxb/eru072 | | LOC_Os11g03370 |  | ONAC045 | NAC | RT | Drought tolerance | Overexpression | Drought and salinity tolerance. | 10.1016/j.bbrc.2008.12.163 | | LOC_Os03g02800 |  | rim1 | NAC | RT | Other disease resistance | Mutant | Rice dwarf virus resistance. | 10.1111/j.1365-313X.2008.03712.x | | LOC_Os05g35170 |  | IDEF2 | NAC | RT | Other soil stress tolerance | Knockdown | Fe homeostais. | 10.1074/jbc.M708732200 | | LOC_Os12g03040 |  | ONAC131 | NAC | RT | Blast resistance | Knockdown | Resistance to Magnaporthe grisea. | 10.1007/s11033-012-2040-y | | LOC_Os11g03300 |  | OsNAC10 | NAC | RT | Drought tolerance | Overexpression | Grain yield under drought conditions. | 10.1104/pp.110.154773 | | LOC_Os11g08210 | MF | OsNAC5 | NAC | RT | Cold tolerance | Knockdown Overexpression | Cold tolerance. Sensitivity to salt, mannitol and ABA during germination. | 10.1007/s00425-011-1403-2 | | LOC_Os01g66120 | MF | SNAC2 | NAC | RT | Cold tolerance | Overexpression | Cold and salinity tolerance. | 10.1007/s11103-008-9309-5 | | LOC_Os01g60020 |  | OsNAC4 | NAC | RT | Bacterial blight resistance | Knockdown | Bacterial blight resistance. HR cell death. | 10.1038/emboj.2009.39 | | LOC_Os03g60080 |  | SNAC1 | NAC | RT | Drought tolerance | Overexpression | Drought and salinity tolerance. Stomatal control. | 10.1073/pnas.0604882103 | | LOC_Os12g07790 |  | NAC | NAC | RT | Salinity tolerance | Natural variation | regulation of NaCl stress responses | 10.1007/s11738-014-1569-x | | LOC_Os10g09820 |  | OsNAC111 | NAC | RT | Blast resistance | Overexpression | enhanced blast resistance | 10.1094/mpmi-03-14-0065-r | | LOC_Os08g39890 | MF | IPA1 | SBP | RT | Lodging resistance | Natural variation | Panicle branching. Tillering. Culm mechanical strength. | 10.1038/ng.591 | | LOC_Os03g08310 |  | OsTIFY11a | Tify | RT | Drought tolerance | Overexpression | Drought and salinity tolerance. | 10.1007/s11103-009-9524-8 | | LOC_Os09g26780 |  | OsJAZ8 | Tify | RT | Bacterial blight resistance | Overexpression | JA induced resistance to Xanthomonas oryzae pv. oryzae. | 10.1093/pcp/pcs145 | | LOC_Os09g23650 |  | OsJAZ8 | Tify | RT | Blast resistance | Overexpression | regulation of JA-induced accumulation of linalool and role in resistance to Xoo | 10.1111/pce.12169 | | LOC_Os05g43950 |  | OsVOZ2 | VOZ | RT | Bacterial blight resistance | Mutant | Resistance to Xanthomonas oryzae pv oryzae after flowering. | 10.1371/journal.pone.0073346 | | LOC_Os01g54600 |  | OsWRKY13 | WRKY | RT | Bacterial blight resistance | Overexpression | Bacterial blight and blast resistance. | 10.1094/MPMI-20-5-0492 | | LOC_Os05g27730 |  | OsWRKY53 | WRKY | RT | Blast resistance | Overexpression | Resistance to Magnaporthe grisea. | 10.1016/j.bbaexp.2007.04.006 | | LOC_Os08g38990 |  | WRKY30 | WRKY | RT | Bacterial blight resistance | Overexpression | Resistance to Magnaporthe grisea and Rhizoctonia solani. Regulation of JA level. | 10.1007/s00425-012-1668-0 | | LOC_Os01g43650 | MF | OsWRKY11 | WRKY | RT | Drought tolerance | Overexpression | Drought and heat tolerance. | 10.1007/s00299-008-0614-x | | LOC_Os09g25060 |  | OsWRKY76 | WRKY | RT | Bacterial blight resistance | Overexpression | Resistance to Xanthomonas oryzae pv. oryzae. | 10.1007/s12284-010-9039-6 | | LOC_Os09g25070 |  | OsWRKY62 | WRKY | RT | Bacterial blight resistance | Overexpression | Resistance to Xanthomonas oryzae pv. oryzae. | 10.1093/mp/ssn024 | | LOC_Os06g44010 |  | OsWRKY28 | WRKY | RT | Bacterial blight resistance | Overexpression | Resistance to Xanthomonas oryzae pv. oryzae. | 10.1007/s12284-010-9039-6 | | LOC_Os02g08440 |  | OsWRKY71 | WRKY | RT | Bacterial blight resistance | Overexpression | Resistance to Xanthomonas oryzae pv. oryzae. | 10.1016/j.jplph.2006.07.006 | | LOC_Os11g02520 | MF | OsWRKY89 | WRKY | RT | Blast resistance | Knockdown Overexpression | Blast, UV and white-backed planthopper resistance. Internode elongation. Growth retardation at the early stage. | 10.1007/s11103-007-9244-x | | LOC_Os03g20550 | MF | OsWRKY31 | WRKY | RT | Blast resistance | Knockdown Overexpression | Resistance to Magnaporthe grisea. Lateral root growth. | 10.1038/cr.2007.104 | | LOC_Os05g25770 |  | OsWRKY45 | WRKY | RT | Cold tolerance | Knockdown Overexpression | Cold, drought and salinity tolerance. ABA sensitivity. | 10.1093/jxb/err144 | |
|  |
| MF-Multiple Function; MT-Morphological Trait; PT-Physiological Trait; RT-Resistance or Tolerance |
|  |
|  |
|  |
|  |
|  |
|  |
|  |
|  |
|  |

**Table S2.** Summary of PCC analysis of genome-wide transcription factor and transcription regulator genes with their paralogs.

| **Transcription factor / transcription regulator Paralog PCC** |
| --- |
| | LOC_Os01g01430 | LOC_Os01g01470 | 0.133815993 | | --- | --- | --- | | LOC_Os01g01470 | LOC_Os01g01430 | 0.133815993 | | LOC_Os01g01840 | LOC_Os01g01870 | 0.448230878 | | LOC_Os01g01870 | LOC_Os01g01840 | 0.448230878 | | LOC_Os01g01960 | LOC_Os05g01020 | 0.714593787 | | LOC_Os01g04750 | LOC_Os01g04800 | 0.659953017 | | LOC_Os01g04800 | LOC_Os01g04750 | 0.659953017 | | LOC_Os01g06320 | LOC_Os05g07010 | 0.546571276 | | LOC_Os01g06640 | LOC_Os05g07120 | 0.177910951 | | LOC_Os01g08320 | LOC_Os05g08570 | 0.545283617 | | LOC_Os01g08970 | LOC_Os05g08970 | -0.003133809 | | LOC_Os01g09100 | LOC_Os05g09020 | 0.550237813 | | LOC_Os01g09450 | LOC_Os05g09480 | 0.643249087 | | LOC_Os01g09550 | LOC_Os05g10620 | -0.248630794 | | LOC_Os01g09620 | LOC_Os05g10670 | 0.484360015 | | LOC_Os01g09640 | LOC_Os05g10690 | 0.512806695 | | LOC_Os01g09900 | LOC_Os01g09930 | 0.541605768 | | LOC_Os01g10370 | LOC_Os02g13710 | 0.153679946 | | LOC_Os01g10504 | LOC_Os05g11414 | 0.904078937 | | LOC_Os01g10580 | LOC_Os05g11510 | 0.271854666 | | LOC_Os01g10610 | LOC_Os02g13900 | 0.189628549 | | LOC_Os01g11350 | LOC_Os01g55150 | 0.628788266 | | LOC_Os01g11952 | LOC_Os01g11960 | 0.336537099 | | LOC_Os01g11960 | LOC_Os01g11952 | 0.336537099 | | LOC_Os01g13030 | LOC_Os05g14180 | 0.84207369 | | LOC_Os01g15350 | LOC_Os01g15460 | 0.211406943 | | LOC_Os01g15460 | LOC_Os01g15350 | 0.211406943 | | LOC_Os01g18240 | LOC_Os05g04820 | 0.906457752 | | LOC_Os01g19130 | LOC_Os05g04360 | 0.510654947 | | LOC_Os01g19330 | LOC_Os05g04210 | 0.181471164 | | LOC_Os01g19694 | LOC_Os05g03884 | 0.476084788 | | LOC_Os01g21120 | LOC_Os05g29810 | 0.762952535 | | LOC_Os01g27040 | LOC_Os05g05780 | -0.183732359 | | LOC_Os01g33350 | LOC_Os01g33370 | 0.740108286 | | LOC_Os01g33370 | LOC_Os01g33350 | 0.740108286 | | LOC_Os01g39040 | LOC_Os01g39070 | 0.275135381 | | LOC_Os01g39070 | LOC_Os01g39040 | 0.275135381 | | LOC_Os01g39150 | LOC_Os01g39160 | 0.510563777 | | LOC_Os01g39160 | LOC_Os01g39180 | 0.529746183 | | LOC_Os01g40260 | LOC_Os01g51690 | 0.216164796 | | LOC_Os01g41900 | LOC_Os05g51160 | 0.189534061 | | LOC_Os01g43550 | LOC_Os05g50700 | 0.637955859 | | LOC_Os01g45730 | LOC_Os05g50080 | 0.498718267 | | LOC_Os01g45860 | LOC_Os05g49930 | 0.304487258 | | LOC_Os01g46800 | LOC_Os05g49620 | 0.312110031 | | LOC_Os01g46970 | LOC_Os05g49420 | 0.579525379 | | LOC_Os01g48060 | LOC_Os01g54990 | 0.787722576 | | LOC_Os01g48130 | LOC_Os05g48850 | 0.777848917 | | LOC_Os01g48180 | LOC_Os05g48820 | 0.09145835 | | LOC_Os01g48290 | LOC_Os01g55340 | 0.444916382 | | LOC_Os01g48320 | LOC_Os05g48690 | 0.400759638 | | LOC_Os01g48370 | LOC_Os01g55430 | -0.503407918 | | LOC_Os01g48444 | LOC_Os05g48590 | -0.289834298 | | LOC_Os01g49160 | LOC_Os05g48010 | 0.301193 | | LOC_Os01g49830 | LOC_Os05g47650 | 0.531667826 | | LOC_Os01g50720 | LOC_Os05g46610 | 0.752592303 | | LOC_Os01g51140 | LOC_Os05g46370 | 0.453512389 | | LOC_Os01g51690 | LOC_Os05g46020 | 0.327931862 | | LOC_Os01g52514 | LOC_Os01g52540 | -0.030612734 | | LOC_Os01g52540 | LOC_Os01g52514 | -0.030612734 | | LOC_Os01g53040 | LOC_Os01g54600 | 0.361974974 | | LOC_Os01g53260 | LOC_Os05g45230 | 0.100177223 | | LOC_Os01g53650 | LOC_Os05g45020 | 0.579026369 | | LOC_Os01g53880 | LOC_Os05g44810 | 0.074379526 | | LOC_Os01g54210 | LOC_Os05g44400 | 0.653560127 | | LOC_Os01g54600 | LOC_Os01g53040 | 0.361974974 | | LOC_Os01g54890 | LOC_Os05g49010 | 0.059312941 | | LOC_Os01g54930 | LOC_Os05g43950 | -0.086494559 | | LOC_Os01g54990 | LOC_Os05g43920 | 0.448841153 | | LOC_Os01g55150 | LOC_Os01g11350 | 0.628788266 | | LOC_Os01g55340 | LOC_Os01g48290 | 0.444916382 | | LOC_Os01g55430 | LOC_Os05g43850 | 0.460295042 | | LOC_Os01g55750 | LOC_Os05g43760 | 0.635943046 | | LOC_Os01g56100 | LOC_Os05g43520 | 0.308292886 | | LOC_Os01g57240 | LOC_Os05g42290 | 0.135581649 | | LOC_Os01g58420 | LOC_Os05g41780 | 0.853192952 | | LOC_Os01g58760 | LOC_Os05g41540 | 0.48152274 | | LOC_Os01g59660 | LOC_Os05g41166 | 0.474531171 | | LOC_Os01g59780 | LOC_Os11g03540 | 0.579029699 | | LOC_Os01g60020 | LOC_Os03g21060 | 0.346769421 | | LOC_Os01g60520 | LOC_Os01g60600 | -0.010109907 | | LOC_Os01g60540 | LOC_Os05g40070 | 0.114939023 | | LOC_Os01g60600 | LOC_Os01g60520 | -0.010109907 | | LOC_Os01g60640 | LOC_Os05g40060 | -0.135560124 | | LOC_Os01g60810 | LOC_Os03g21870 | 0.175299183 | | LOC_Os01g61080 | LOC_Os05g39720 | 0.894380529 | | LOC_Os01g61810 | LOC_Os05g38820 | -0.097155849 | | LOC_Os01g61900 | LOC_Os05g38990 | 0.283640793 | | LOC_Os01g62410 | LOC_Os05g38460 | 0.308120716 | | LOC_Os01g62460 | LOC_Os11g47870 | 0.680773896 | | LOC_Os01g62920 | LOC_Os05g38120 | 0.757207306 | | LOC_Os01g63460 | LOC_Os05g37730 | 0.585963429 | | LOC_Os01g63980 | LOC_Os05g37190 | 0.257745147 | | LOC_Os01g64020 | LOC_Os05g37170 | 0.528630517 | | LOC_Os01g64310 | LOC_Os05g37080 | 0.526336422 | | LOC_Os01g64360 | LOC_Os05g37060 | 0.722041344 | | LOC_Os01g64410 | LOC_Os04g33870 | 0.428274403 | | LOC_Os01g64430 | LOC_Os05g36970 | -0.065169813 | | LOC_Os01g64590 | LOC_Os05g36900 | 0.249101791 | | LOC_Os01g64700 | LOC_Os04g59130 | 0.492658062 | | LOC_Os01g64730 | LOC_Os05g36160 | 0.090860196 | | LOC_Os01g65370 | LOC_Os05g35500 | 0.481515624 | | LOC_Os01g66120 | LOC_Os05g34830 | 0.824641709 | | LOC_Os01g66420 | LOC_Os05g34640 | 0.524537342 | | LOC_Os01g66490 | LOC_Os05g34600 | 0.827947601 | | LOC_Os01g66590 | LOC_Os05g34450 | 0.797084618 | | LOC_Os01g68700 | LOC_Os08g41320 | 0.364960836 | | LOC_Os01g69830 | LOC_Os08g41940 | 0.501188105 | | LOC_Os01g69850 | LOC_Os08g41960 | -0.012245977 | | LOC_Os01g69910 | LOC_Os04g31900 | 0.689629344 | | LOC_Os01g70110 | LOC_Os08g42400 | 0.280831258 | | LOC_Os01g70880 | LOC_Os01g70890 | -0.01262144 | | LOC_Os01g70890 | LOC_Os01g70880 | -0.01262144 | | LOC_Os01g71970 | LOC_Os05g31420 | 0.03083948 | | LOC_Os02g04490 | LOC_Os06g49130 | 0.01183488 | | LOC_Os02g04680 | LOC_Os06g49010 | 0.854469375 | | LOC_Os02g04810 | LOC_Os06g48950 | 0.47727921 | | LOC_Os02g05470 | LOC_Os06g48610 | 0.183614136 | | LOC_Os02g05510 | LOC_Os06g48534 | 0.409149246 | | LOC_Os02g05640 | LOC_Os06g48290 | 0.60209635 | | LOC_Os02g06330 | LOC_Os06g47590 | 0.637640867 | | LOC_Os02g06584 | LOC_Os06g46890 | 0.502525704 | | LOC_Os02g06910 | LOC_Os06g46410 | 0.749634899 | | LOC_Os02g06950 | LOC_Os06g46270 | 0.454014706 | | LOC_Os02g07170 | LOC_Os06g45890 | 0.540649059 | | LOC_Os02g07430 | LOC_Os06g45650 | 0.480066063 | | LOC_Os02g07450 | LOC_Os06g45640 | -0.128467804 | | LOC_Os02g07770 | LOC_Os06g45410 | 0.538647139 | | LOC_Os02g07780 | LOC_Os06g45310 | 0.694154906 | | LOC_Os02g07840 | LOC_Os06g45140 | 0.117583797 | | LOC_Os02g07930 | LOC_Os06g45040 | 0.334016928 | | LOC_Os02g08070 | LOC_Os06g44860 | 0.228130828 | | LOC_Os02g08150 | LOC_Os06g44450 | 0.692670731 | | LOC_Os02g08400 | LOC_Os06g44100 | 0.320434682 | | LOC_Os02g08440 | LOC_Os06g44010 | 0.783273293 | | LOC_Os02g08500 | LOC_Os06g43910 | -0.338888427 | | LOC_Os02g08544 | LOC_Os06g43860 | 0.873487912 | | LOC_Os02g09480 | LOC_Os06g43090 | 0.680360847 | | LOC_Os02g09910 | LOC_Os02g09920 | -0.117021769 | | LOC_Os02g09920 | LOC_Os02g09910 | -0.117021769 | | LOC_Os02g10000 | LOC_Os06g41930 | 0.283664168 | | LOC_Os02g10080 | LOC_Os06g41384 | 0.547797731 | | LOC_Os02g10360 | LOC_Os06g40780 | 0.002740419 | | LOC_Os02g10860 | LOC_Os06g39960 | 0.320348798 | | LOC_Os02g12790 | LOC_Os06g37450 | 0.702038104 | | LOC_Os02g12820 | LOC_Os06g37410 | 0.090922754 | | LOC_Os02g13710 | LOC_Os06g36000 | 0.444060655 | | LOC_Os02g13800 | LOC_Os06g35960 | 0.633547605 | | LOC_Os02g13900 | LOC_Os06g35900 | -0.226757716 | | LOC_Os02g14490 | LOC_Os06g35140 | 0.216836492 | | LOC_Os02g14910 | LOC_Os01g11350 | 0.594009492 | | LOC_Os02g15340 | LOC_Os06g33940 | 0.834507572 | | LOC_Os02g15760 | LOC_Os06g33450 | 0.379464705 | | LOC_Os02g28580 | LOC_Os02g28660 | 0.805217835 | | LOC_Os02g28660 | LOC_Os02g28580 | 0.805217835 | | LOC_Os02g32140 | LOC_Os04g32620 | 0.667197886 | | LOC_Os02g34260 | LOC_Os04g34970 | 0.533833781 | | LOC_Os02g34320 | LOC_Os04g35010 | -0.220493529 | | LOC_Os02g34970 | LOC_Os04g35660 | 0.533870865 | | LOC_Os02g35140 | LOC_Os04g36054 | 0.638034768 | | LOC_Os02g35460 | LOC_Os04g36650 | -0.057496479 | | LOC_Os02g35600 | LOC_Os04g36730 | 0.580733867 | | LOC_Os02g35690 | LOC_Os04g36790 | 0.416507239 | | LOC_Os02g36510 | LOC_Os04g38400 | -0.110194341 | | LOC_Os02g36880 | LOC_Os04g38720 | 0.446861749 | | LOC_Os02g36890 | LOC_Os04g38740 | 0.788790785 | | LOC_Os02g39140 | LOC_Os04g41229 | 0.811189294 | | LOC_Os02g39710 | LOC_Os04g42020 | 0.598992258 | | LOC_Os02g40070 | LOC_Os04g42570 | 0.388834175 | | LOC_Os02g40530 | LOC_Os04g42950 | 0.04829584 | | LOC_Os02g41450 | LOC_Os04g43560 | 0.667632606 | | LOC_Os02g41510 | LOC_Os04g43680 | 0.567581869 | | LOC_Os02g42380 | LOC_Os04g44440 | 0.536912793 | | LOC_Os02g42820 | LOC_Os04g45010 | 0.851609195 | | LOC_Os02g42850 | LOC_Os04g45020 | 0.648523964 | | LOC_Os02g42870 | LOC_Os04g45060 | 0.499067151 | | LOC_Os02g42950 | LOC_Os04g45330 | 0.874476389 | | LOC_Os02g43170 | LOC_Os04g45690 | 0.43624257 | | LOC_Os02g43300 | LOC_Os04g45750 | 0.615849402 | | LOC_Os02g43330 | LOC_Os04g45810 | 0.481341665 | | LOC_Os02g43560 | LOC_Os04g46060 | 0.884060721 | | LOC_Os02g43790 | LOC_Os04g46220 | 0.752219258 | | LOC_Os02g43820 | LOC_Os04g46250 | 0.273140364 | | LOC_Os02g43940 | LOC_Os02g43970 | 0.527649642 | | LOC_Os02g43970 | LOC_Os02g43940 | 0.527649642 | | LOC_Os02g44120 | LOC_Os04g46670 | 0.835693301 | | LOC_Os02g44130 | LOC_Os04g46680 | 0.495213825 | | LOC_Os02g44360 | LOC_Os02g44370 | 0.912512498 | | LOC_Os02g44370 | LOC_Os02g44360 | 0.912512498 | | LOC_Os02g44930 | LOC_Os04g47690 | 0.381805447 | | LOC_Os02g45054 | LOC_Os04g47860 | 0.602734023 | | LOC_Os02g45080 | LOC_Os04g47890 | 0.353753148 | | LOC_Os02g45200 | LOC_Os04g47990 | 0.816422824 | | LOC_Os02g45250 | LOC_Os04g48070 | 0.250178581 | | LOC_Os02g45450 | LOC_Os04g48350 | 0.731899868 | | LOC_Os02g45570 | LOC_Os04g48510 | 0.824042942 | | LOC_Os02g45620 | LOC_Os04g48830 | 0.205381041 | | LOC_Os02g45760 | LOC_Os04g49110 | 0.633790201 | | LOC_Os02g45770 | LOC_Os04g49150 | 0.894678717 | | LOC_Os02g45850 | LOC_Os04g49230 | 0.642102196 | | LOC_Os02g46030 | LOC_Os04g49450 | 0.23166109 | | LOC_Os02g46560 | LOC_Os04g50090 | 0.667573795 | | LOC_Os02g46610 | LOC_Os04g50120 | 0.573809336 | | LOC_Os02g46780 | LOC_Os04g50770 | 0.718450064 | | LOC_Os02g46930 | LOC_Os10g42690 | 0.787911579 | | LOC_Os02g47060 | LOC_Os04g50920 | 0.57667315 | | LOC_Os02g47280 | LOC_Os04g51190 | 0.859714712 | | LOC_Os02g48650 | LOC_Os09g31310 | 0.355047198 | | LOC_Os02g49230 | LOC_Os06g19444 | 0.806239964 | | LOC_Os02g49440 | LOC_Os08g38220 | 0.615626779 | | LOC_Os02g49480 | LOC_Os08g38210 | 0.327495961 | | LOC_Os02g49560 | LOC_Os08g38020 | -0.165300403 | | LOC_Os02g49986 | LOC_Os06g14670 | 0.346391623 | | LOC_Os02g50630 | LOC_Os06g13670 | 0.930378027 | | LOC_Os02g51090 | LOC_Os06g12360 | 0.357081497 | | LOC_Os02g51320 | LOC_Os06g12210 | 0.705949739 | | LOC_Os02g51450 | LOC_Os06g12100 | 0.64211024 | | LOC_Os02g51670 | LOC_Os06g11860 | 0.652317199 | | LOC_Os02g51799 | LOC_Os06g11780 | 0.421791417 | | LOC_Os02g52190 | LOC_Os09g29360 | 0.513011338 | | LOC_Os02g52340 | LOC_Os06g11330 | 0.74757808 | | LOC_Os02g52670 | LOC_Os08g36920 | 0.805265432 | | LOC_Os02g52780 | LOC_Os06g10880 | -0.235456224 | | LOC_Os02g52960 | LOC_Os06g10690 | 0.593178569 | | LOC_Os02g53360 | LOC_Os06g10570 | 0.725328909 | | LOC_Os02g53690 | LOC_Os06g10310 | 0.320318025 | | LOC_Os02g54050 | LOC_Os06g09810 | 0.524521407 | | LOC_Os02g54160 | LOC_Os06g09390 | 0.59917525 | | LOC_Os02g54520 | LOC_Os09g26170 | 0.624547288 | | LOC_Os02g55250 | LOC_Os06g08500 | 0.19851468 | | LOC_Os02g55320 | LOC_Os06g08440 | 0.641173068 | | LOC_Os02g55560 | LOC_Os06g08140 | 0.165037023 | | LOC_Os02g56120 | LOC_Os06g07040 | 0.662767483 | | LOC_Os02g56140 | LOC_Os06g06900 | 0.251009186 | | LOC_Os02g57490 | LOC_Os03g05510 | 0.718931108 | | LOC_Os03g02160 | LOC_Os10g36810 | -0.036534791 | | LOC_Os03g02240 | LOC_Os10g37240 | 0.809047247 | | LOC_Os03g02900 | LOC_Os04g49230 | 0.567373669 | | LOC_Os03g03100 | LOC_Os04g49150 | -0.324423747 | | LOC_Os03g03260 | LOC_Os10g39030 | 0.85328421 | | LOC_Os03g03480 | LOC_Os04g48830 | 0.036706544 | | LOC_Os03g03540 | LOC_Os10g38834 | 0.316226507 | | LOC_Os03g03550 | LOC_Os10g38820 | 0.493372607 | | LOC_Os03g03760 | LOC_Os10g39550 | 0.259387321 | | LOC_Os03g04310 | LOC_Os10g39750 | 0.574352712 | | LOC_Os03g04620 | LOC_Os10g32900 | 0.745587495 | | LOC_Os03g05160 | LOC_Os10g32070 | -0.03437087 | | LOC_Os03g05500 | LOC_Os03g05510 | 0.586924535 | | LOC_Os03g05510 | LOC_Os03g05500 | 0.586924535 | | LOC_Os03g05760 | LOC_Os10g30420 | 0.214012305 | | LOC_Os03g06350 | LOC_Os10g29610 | -0.106772998 | | LOC_Os03g06630 | LOC_Os03g58160 | 0.478305181 | | LOC_Os03g07360 | LOC_Os10g26620 | 0.795569651 | | LOC_Os03g07450 | LOC_Os10g26500 | 0.858512675 | | LOC_Os03g07880 | LOC_Os10g25850 | 0.224173872 | | LOC_Os03g08310 | LOC_Os03g08320 | 0.59854703 | | LOC_Os03g08320 | LOC_Os03g08330 | 0.810466132 | | LOC_Os03g08330 | LOC_Os03g08320 | 0.810466132 | | LOC_Os03g08460 | LOC_Os03g08470 | 0.252901372 | | LOC_Os03g08470 | LOC_Os03g08490 | 0.224652152 | | LOC_Os03g08490 | LOC_Os03g08500 | 0.618862174 | | LOC_Os03g08500 | LOC_Os03g08490 | 0.618862174 | | LOC_Os03g08930 | LOC_Os09g29360 | 0.656878594 | | LOC_Os03g08960 | LOC_Os09g29460 | 0.433524417 | | LOC_Os03g09100 | LOC_Os07g43030 | 0.340027102 | | LOC_Os03g09170 | LOC_Os10g22600 | 0.279072827 | | LOC_Os03g09280 | LOC_Os10g22430 | 0.489385012 | | LOC_Os03g16850 | LOC_Os05g02150 | 0.165784555 | | LOC_Os03g17570 | LOC_Os07g49460 | 0.707772471 | | LOC_Os03g17810 | LOC_Os05g03160 | -0.072182253 | | LOC_Os03g19020 | LOC_Os07g49030 | 0.387921457 | | LOC_Os03g20090 | LOC_Os07g48870 | 0.34733212 | | LOC_Os03g20780 | LOC_Os03g20790 | 0.969940803 | | LOC_Os03g20790 | LOC_Os03g20780 | 0.969940803 | | LOC_Os03g20900 | LOC_Os05g40960 | 0.766030734 | | LOC_Os03g20910 | LOC_Os07g48560 | 0.764643256 | | LOC_Os03g21030 | LOC_Os03g21060 | 0.652770691 | | LOC_Os03g21060 | LOC_Os03g21030 | 0.652770691 | | LOC_Os03g21140 | LOC_Os03g21160 | 0.578531115 | | LOC_Os03g21160 | LOC_Os03g21140 | 0.578531115 | | LOC_Os03g21710 | LOC_Os05g40060 | -0.013209356 | | LOC_Os03g21800 | LOC_Os07g48180 | 0.681694011 | | LOC_Os03g22170 | LOC_Os07g47790 | 0.411220585 | | LOC_Os03g22770 | LOC_Os07g47140 | 0.275070181 | | LOC_Os03g22800 | LOC_Os07g47110 | 0.203247097 | | LOC_Os03g25120 | LOC_Os07g44690 | 0.247088717 | | LOC_Os03g25550 | LOC_Os07g44090 | 0.34088415 | | LOC_Os03g26130 | LOC_Os07g43580 | 0.088173124 | | LOC_Os03g26210 | LOC_Os07g43530 | 0.11339705 | | LOC_Os03g28940 | LOC_Os07g42370 | 0.420780267 | | LOC_Os03g29760 | LOC_Os07g41720 | 0.70508518 | | LOC_Os03g29970 | LOC_Os07g41580 | 0.672627343 | | LOC_Os03g31240 | LOC_Os07g40780 | 0.65082444 | | LOC_Os03g39050 | LOC_Os03g39100 | 0.198336108 | | LOC_Os03g39100 | LOC_Os03g39050 | 0.198336108 | | LOC_Os03g42100 | LOC_Os12g39850 | 0.356790469 | | LOC_Os03g42230 | LOC_Os03g42240 | 0.349308361 | | LOC_Os03g42240 | LOC_Os03g42230 | 0.349308361 | | LOC_Os03g42280 | LOC_Os03g42290 | 0.305460721 | | LOC_Os03g42290 | LOC_Os03g42280 | 0.305460721 | | LOC_Os03g42420 | LOC_Os03g42430 | -0.044387313 | | LOC_Os03g42430 | LOC_Os03g42420 | -0.044387313 | | LOC_Os03g42820 | LOC_Os12g40490 | 0.806891082 | | LOC_Os03g43400 | LOC_Os12g40890 | 0.665846834 | | LOC_Os03g43410 | LOC_Os12g40900 | 0.870969454 | | LOC_Os03g43730 | LOC_Os12g41230 | 0.464723604 | | LOC_Os03g43810 | LOC_Os12g41650 | 0.783866829 | | LOC_Os03g43930 | LOC_Os12g41860 | 0.839258454 | | LOC_Os03g44540 | LOC_Os07g06470 | 0.356954996 | | LOC_Os03g44710 | LOC_Os07g06620 | 0.856790356 | | LOC_Os03g46200 | LOC_Os03g46250 | 0.243277988 | | LOC_Os03g46250 | LOC_Os03g46200 | 0.243277988 | | LOC_Os03g46860 | LOC_Os12g43620 | 0.318507629 | | LOC_Os03g47740 | LOC_Os12g43950 | 0.734235225 | | LOC_Os03g53050 | LOC_Os03g58420 | 0.149274232 | | LOC_Os03g53150 | LOC_Os03g58350 | 0.183445543 | | LOC_Os03g53340 | LOC_Os03g58160 | -0.003776084 | | LOC_Os03g54160 | LOC_Os03g54170 | 0.833241706 | | LOC_Os03g54170 | LOC_Os03g54160 | 0.833241706 | | LOC_Os03g55080 | LOC_Os07g02060 | 0.461597868 | | LOC_Os03g55530 | LOC_Os07g02540 | 0.201685658 | | LOC_Os03g55590 | LOC_Os07g02800 | 0.562285113 | | LOC_Os03g56010 | LOC_Os07g03220 | 0.413712816 | | LOC_Os03g56050 | LOC_Os07g03250 | 0.682203206 | | LOC_Os03g56580 | LOC_Os07g04560 | 0.439484735 | | LOC_Os03g56950 | LOC_Os07g05010 | 0.726281702 | | LOC_Os03g57190 | LOC_Os07g05720 | 0.697692628 | | LOC_Os03g58160 | LOC_Os07g08140 | -0.04634273 | | LOC_Os03g58250 | LOC_Os07g08420 | 0.122847792 | | LOC_Os03g58350 | LOC_Os07g08460 | 0.282182141 | | LOC_Os03g58420 | LOC_Os03g53050 | 0.149274232 | | LOC_Os03g58530 | LOC_Os07g08880 | -0.11909254 | | LOC_Os03g58640 | LOC_Os07g09320 | 0.187889858 | | LOC_Os03g59460 | LOC_Os07g10890 | -0.001542639 | | LOC_Os03g59530 | LOC_Os07g11010 | 0.641705128 | | LOC_Os03g60080 | LOC_Os07g12340 | 0.35841446 | | LOC_Os03g60120 | LOC_Os07g12510 | 0.570094054 | | LOC_Os03g60430 | LOC_Os07g13170 | -0.090560321 | | LOC_Os03g60630 | LOC_Os07g13260 | 0.126661813 | | LOC_Os04g27960 | LOC_Os04g27990 | 0.643487062 | | LOC_Os04g27990 | LOC_Os04g27960 | 0.643487062 | | LOC_Os04g28120 | LOC_Os04g28130 | 0.724224001 | | LOC_Os04g31900 | LOC_Os01g69910 | 0.689629344 | | LOC_Os04g32620 | LOC_Os02g32140 | 0.667197886 | | LOC_Os04g33420 | LOC_Os04g33440 | 0.162192069 | | LOC_Os04g33440 | LOC_Os04g33420 | 0.162192069 | | LOC_Os04g33870 | LOC_Os05g36990 | 0.451043677 | | LOC_Os04g34970 | LOC_Os02g34260 | 0.533833781 | | LOC_Os04g35010 | LOC_Os02g34320 | -0.220493529 | | LOC_Os04g35660 | LOC_Os02g34970 | 0.533870865 | | LOC_Os04g36054 | LOC_Os02g35140 | 0.638034768 | | LOC_Os04g36650 | LOC_Os02g35460 | -0.057496479 | | LOC_Os04g36730 | LOC_Os02g35600 | 0.580733867 | | LOC_Os04g36790 | LOC_Os02g35690 | 0.416507239 | | LOC_Os04g38400 | LOC_Os02g36510 | -0.110194341 | | LOC_Os04g38720 | LOC_Os08g33670 | 0.258670969 | | LOC_Os04g38740 | LOC_Os08g33660 | 0.704198859 | | LOC_Os04g41229 | LOC_Os02g39140 | 0.811189294 | | LOC_Os04g42020 | LOC_Os02g39710 | 0.598992258 | | LOC_Os04g42570 | LOC_Os02g40070 | 0.388834175 | | LOC_Os04g42950 | LOC_Os02g40530 | 0.04829584 | | LOC_Os04g43560 | LOC_Os02g41450 | 0.667632606 | | LOC_Os04g43680 | LOC_Os02g41510 | 0.567581869 | | LOC_Os04g44440 | LOC_Os02g42380 | 0.536912793 | | LOC_Os04g45010 | LOC_Os02g42820 | 0.851609195 | | LOC_Os04g45020 | LOC_Os04g45060 | 0.125379342 | | LOC_Os04g45060 | LOC_Os04g45020 | 0.125379342 | | LOC_Os04g45330 | LOC_Os02g42950 | 0.874476389 | | LOC_Os04g45690 | LOC_Os02g43170 | 0.43624257 | | LOC_Os04g45750 | LOC_Os02g43300 | 0.615849402 | | LOC_Os04g45810 | LOC_Os02g43330 | 0.481341665 | | LOC_Os04g46060 | LOC_Os02g43560 | 0.884060721 | | LOC_Os04g46220 | LOC_Os04g46250 | 0.383249149 | | LOC_Os04g46250 | LOC_Os04g46220 | 0.383249149 | | LOC_Os04g46350 | LOC_Os10g41230 | 0.305697303 | | LOC_Os04g46400 | LOC_Os04g46410 | -0.19516187 | | LOC_Os04g46410 | LOC_Os04g46440 | -0.194413004 | | LOC_Os04g46440 | LOC_Os04g46410 | -0.194413004 | | LOC_Os04g46670 | LOC_Os02g44120 | 0.835693301 | | LOC_Os04g46680 | LOC_Os02g44130 | 0.495213825 | | LOC_Os04g46860 | LOC_Os02g44370 | 0.78794205 | | LOC_Os04g47040 | LOC_Os04g47059 | 0.154658469 | | LOC_Os04g47059 | LOC_Os04g47040 | 0.154658469 | | LOC_Os04g47690 | LOC_Os06g51220 | 0.157696735 | | LOC_Os04g47860 | LOC_Os02g45054 | 0.602734023 | | LOC_Os04g47890 | LOC_Os02g45080 | 0.353753148 | | LOC_Os04g47990 | LOC_Os02g45200 | 0.816422824 | | LOC_Os04g48030 | LOC_Os09g35790 | 0.645357912 | | LOC_Os04g48070 | LOC_Os09g35760 | 0.043159684 | | LOC_Os04g48350 | LOC_Os09g35010 | 0.804096918 | | LOC_Os04g48510 | LOC_Os02g45570 | 0.824042942 | | LOC_Os04g48830 | LOC_Os02g45620 | 0.205381041 | | LOC_Os04g49110 | LOC_Os02g45760 | 0.633790201 | | LOC_Os04g49150 | LOC_Os02g45770 | 0.894678717 | | LOC_Os04g49230 | LOC_Os02g45850 | 0.642102196 | | LOC_Os04g49450 | LOC_Os02g46030 | 0.23166109 | | LOC_Os04g50090 | LOC_Os02g46560 | 0.667573795 | | LOC_Os04g50120 | LOC_Os02g46610 | 0.573809336 | | LOC_Os04g50770 | LOC_Os02g46780 | 0.718450064 | | LOC_Os04g50920 | LOC_Os02g47060 | 0.57667315 | | LOC_Os04g51190 | LOC_Os02g47280 | 0.859714712 | | LOC_Os04g51560 | LOC_Os08g13840 | 0.59468987 | | LOC_Os04g52810 | LOC_Os08g10080 | -0.153969646 | | LOC_Os04g53720 | LOC_Os08g08220 | 0.313777376 | | LOC_Os04g54474 | LOC_Os08g07970 | 0.730028562 | | LOC_Os04g55520 | LOC_Os04g55560 | 0.640888892 | | LOC_Os04g55560 | LOC_Os04g55520 | 0.640888892 | | LOC_Os04g58000 | LOC_Os04g58010 | 0.69893355 | | LOC_Os04g58010 | LOC_Os04g58000 | 0.69893355 | | LOC_Os04g58820 | LOC_Os08g01190 | 0.371690265 | | LOC_Os04g59130 | LOC_Os08g01290 | 0.607405831 | | LOC_Os04g59470 | LOC_Os08g01330 | -0.044436909 | | LOC_Os05g01020 | LOC_Os01g01960 | 0.714593787 | | LOC_Os05g02150 | LOC_Os03g16850 | 0.165784555 | | LOC_Os05g03160 | LOC_Os03g17810 | -0.072182253 | | LOC_Os05g03884 | LOC_Os01g19694 | 0.476084788 | | LOC_Os05g04210 | LOC_Os01g19330 | 0.181471164 | | LOC_Os05g04360 | LOC_Os01g19130 | 0.510654947 | | LOC_Os05g04820 | LOC_Os07g44090 | 0.771285562 | | LOC_Os05g05780 | LOC_Os01g27040 | -0.183732359 | | LOC_Os05g07010 | LOC_Os01g06320 | 0.546571276 | | LOC_Os05g07120 | LOC_Os01g06640 | 0.177910951 | | LOC_Os05g08570 | LOC_Os01g08320 | 0.545283617 | | LOC_Os05g08970 | LOC_Os01g08970 | -0.003133809 | | LOC_Os05g09020 | LOC_Os01g09100 | 0.550237813 | | LOC_Os05g09480 | LOC_Os01g09450 | 0.643249087 | | LOC_Os05g10620 | LOC_Os01g09550 | -0.248630794 | | LOC_Os05g10670 | LOC_Os01g09620 | 0.484360015 | | LOC_Os05g10690 | LOC_Os01g09640 | 0.512806695 | | LOC_Os05g11070 | LOC_Os01g09990 | 0.232728767 | | LOC_Os05g11380 | LOC_Os05g11414 | 0.914592663 | | LOC_Os05g11414 | LOC_Os05g11380 | 0.914592663 | | LOC_Os05g11510 | LOC_Os01g10580 | 0.271854666 | | LOC_Os05g14180 | LOC_Os01g13030 | 0.84207369 | | LOC_Os05g29810 | LOC_Os07g47790 | 0.749520819 | | LOC_Os05g33440 | LOC_Os05g33460 | 0.629632065 | | LOC_Os05g33460 | LOC_Os05g33500 | -0.283521749 | | LOC_Os05g33500 | LOC_Os05g33460 | -0.283521749 | | LOC_Os05g34450 | LOC_Os01g66590 | 0.797084618 | | LOC_Os05g34600 | LOC_Os01g66490 | 0.827947601 | | LOC_Os05g34640 | LOC_Os01g66420 | 0.524537342 | | LOC_Os05g34830 | LOC_Os01g66120 | 0.824641709 | | LOC_Os05g35500 | LOC_Os01g65370 | 0.481515624 | | LOC_Os05g36160 | LOC_Os01g64730 | 0.090860196 | | LOC_Os05g36190 | LOC_Os01g64700 | 0.785771752 | | LOC_Os05g36900 | LOC_Os01g64590 | 0.249101791 | | LOC_Os05g36970 | LOC_Os01g64430 | -0.065169813 | | LOC_Os05g36990 | LOC_Os01g64410 | 0.718924731 | | LOC_Os05g37060 | LOC_Os01g64360 | 0.722041344 | | LOC_Os05g37080 | LOC_Os01g64310 | 0.526336422 | | LOC_Os05g37170 | LOC_Os01g64020 | 0.528630517 | | LOC_Os05g37190 | LOC_Os01g63980 | 0.257745147 | | LOC_Os05g37730 | LOC_Os01g63460 | 0.585963429 | | LOC_Os05g38120 | LOC_Os01g62920 | 0.757207306 | | LOC_Os05g38460 | LOC_Os01g62410 | 0.308120716 | | LOC_Os05g38820 | LOC_Os01g61810 | -0.097155849 | | LOC_Os05g38990 | LOC_Os01g61900 | 0.283640793 | | LOC_Os05g39720 | LOC_Os01g61080 | 0.894380529 | | LOC_Os05g40060 | LOC_Os07g48260 | -0.024877235 | | LOC_Os05g40070 | LOC_Os01g60540 | 0.114939023 | | LOC_Os05g40080 | LOC_Os11g02480 | 0.236281488 | | LOC_Os05g40960 | LOC_Os07g48596 | 0.810717253 | | LOC_Os05g41070 | LOC_Os07g48660 | 0.054894462 | | LOC_Os05g41166 | LOC_Os01g59660 | 0.474531171 | | LOC_Os05g41172 | LOC_Os01g59620 | 0.221598298 | | LOC_Os05g41540 | LOC_Os01g58760 | 0.48152274 | | LOC_Os05g41760 | LOC_Os05g41780 | 0.811734882 | | LOC_Os05g41780 | LOC_Os05g41760 | 0.811734882 | | LOC_Os05g42290 | LOC_Os01g57240 | 0.135581649 | | LOC_Os05g43520 | LOC_Os01g56100 | 0.308292886 | | LOC_Os05g43760 | LOC_Os01g55750 | 0.635943046 | | LOC_Os05g43850 | LOC_Os01g55430 | 0.460295042 | | LOC_Os05g43920 | LOC_Os01g54990 | 0.448841153 | | LOC_Os05g43950 | LOC_Os01g54930 | -0.086494559 | | LOC_Os05g44400 | LOC_Os01g54210 | 0.653560127 | | LOC_Os05g44810 | LOC_Os01g53880 | 0.074379526 | | LOC_Os05g45020 | LOC_Os01g53650 | 0.579026369 | | LOC_Os05g45230 | LOC_Os01g53260 | 0.100177223 | | LOC_Os05g46020 | LOC_Os01g51690 | 0.327931862 | | LOC_Os05g46370 | LOC_Os01g51140 | 0.453512389 | | LOC_Os05g46610 | LOC_Os01g50720 | 0.752592303 | | LOC_Os05g47650 | LOC_Os01g49830 | 0.531667826 | | LOC_Os05g48010 | LOC_Os01g49160 | 0.301193 | | LOC_Os05g48590 | LOC_Os01g48444 | -0.289834298 | | LOC_Os05g48670 | LOC_Os01g48370 | 0.206865037 | | LOC_Os05g48690 | LOC_Os01g48320 | 0.400759638 | | LOC_Os05g48820 | LOC_Os01g48180 | 0.09145835 | | LOC_Os05g48850 | LOC_Os01g48130 | 0.777848917 | | LOC_Os05g48870 | LOC_Os01g48060 | 0.822688265 | | LOC_Os05g49010 | LOC_Os01g54890 | 0.059312941 | | LOC_Os05g49420 | LOC_Os01g46970 | 0.579525379 | | LOC_Os05g49620 | LOC_Os01g46800 | 0.312110031 | | LOC_Os05g49930 | LOC_Os01g45860 | 0.304487258 | | LOC_Os05g50080 | LOC_Os01g45730 | 0.498718267 | | LOC_Os05g50700 | LOC_Os01g43550 | 0.637955859 | | LOC_Os05g51160 | LOC_Os01g41900 | 0.189534061 | | LOC_Os06g04850 | LOC_Os06g04870 | 0.742505794 | | LOC_Os06g04870 | LOC_Os06g04850 | 0.742505794 | | LOC_Os06g06900 | LOC_Os02g56140 | 0.251009186 | | LOC_Os06g06970 | LOC_Os06g07030 | 0.157372547 | | LOC_Os06g07030 | LOC_Os06g06970 | 0.157372547 | | LOC_Os06g07040 | LOC_Os02g56120 | 0.662767483 | | LOC_Os06g07640 | LOC_Os06g07650 | 0.316684089 | | LOC_Os06g08140 | LOC_Os02g55560 | 0.165037023 | | LOC_Os06g08440 | LOC_Os02g55320 | 0.641173068 | | LOC_Os06g08500 | LOC_Os02g55250 | 0.19851468 | | LOC_Os06g09390 | LOC_Os02g54160 | 0.59917525 | | LOC_Os06g09810 | LOC_Os02g54050 | 0.524521407 | | LOC_Os06g10310 | LOC_Os02g53690 | 0.320318025 | | LOC_Os06g10570 | LOC_Os02g53360 | 0.725328909 | | LOC_Os06g10690 | LOC_Os02g52960 | 0.593178569 | | LOC_Os06g10820 | LOC_Os09g28210 | 0.165516187 | | LOC_Os06g10880 | LOC_Os09g28310 | -0.193739865 | | LOC_Os06g11330 | LOC_Os02g52340 | 0.74757808 | | LOC_Os06g11780 | LOC_Os02g51799 | 0.421791417 | | LOC_Os06g11860 | LOC_Os02g51670 | 0.652317199 | | LOC_Os06g12040 | LOC_Os06g12050 | 0.201741757 | | LOC_Os06g12050 | LOC_Os06g12060 | 0.098809681 | | LOC_Os06g12060 | LOC_Os06g12070 | 0.709472356 | | LOC_Os06g12070 | LOC_Os06g12060 | 0.709472356 | | LOC_Os06g12080 | LOC_Os06g12100 | 0.221670447 | | LOC_Os06g12100 | LOC_Os06g12080 | 0.221670447 | | LOC_Os06g12210 | LOC_Os02g51320 | 0.705949739 | | LOC_Os06g12230 | LOC_Os02g51280 | 0.453018046 | | LOC_Os06g12360 | LOC_Os02g51090 | 0.357081497 | | LOC_Os06g13670 | LOC_Os02g50630 | 0.930378027 | | LOC_Os06g14670 | LOC_Os02g49986 | 0.346391623 | | LOC_Os06g19444 | LOC_Os02g49230 | 0.806239964 | | LOC_Os06g33450 | LOC_Os02g15760 | 0.379464705 | | LOC_Os06g33940 | LOC_Os02g15340 | 0.834507572 | | LOC_Os06g35140 | LOC_Os02g14490 | 0.216836492 | | LOC_Os06g35900 | LOC_Os01g10610 | 0.624226767 | | LOC_Os06g35960 | LOC_Os02g13800 | 0.633547605 | | LOC_Os06g36000 | LOC_Os01g10370 | 0.327146782 | | LOC_Os06g37410 | LOC_Os02g12820 | 0.090922754 | | LOC_Os06g37450 | LOC_Os02g12790 | 0.702038104 | | LOC_Os06g39960 | LOC_Os02g10860 | 0.320348798 | | LOC_Os06g40780 | LOC_Os02g10360 | 0.002740419 | | LOC_Os06g41384 | LOC_Os02g10080 | 0.547797731 | | LOC_Os06g41930 | LOC_Os02g10000 | 0.283664168 | | LOC_Os06g43090 | LOC_Os02g09480 | 0.680360847 | | LOC_Os06g43860 | LOC_Os02g08544 | 0.873487912 | | LOC_Os06g43910 | LOC_Os02g08500 | -0.338888427 | | LOC_Os06g44010 | LOC_Os02g08440 | 0.783273293 | | LOC_Os06g44100 | LOC_Os02g08400 | 0.320434682 | | LOC_Os06g44450 | LOC_Os02g08150 | 0.692670731 | | LOC_Os06g44860 | LOC_Os02g08070 | 0.228130828 | | LOC_Os06g45040 | LOC_Os02g07930 | 0.334016928 | | LOC_Os06g45140 | LOC_Os02g07840 | 0.117583797 | | LOC_Os06g45310 | LOC_Os02g07780 | 0.694154906 | | LOC_Os06g45640 | LOC_Os02g07450 | -0.128467804 | | LOC_Os06g45650 | LOC_Os02g07430 | 0.480066063 | | LOC_Os06g45890 | LOC_Os02g07170 | 0.540649059 | | LOC_Os06g46270 | LOC_Os02g06950 | 0.454014706 | | LOC_Os06g46410 | LOC_Os02g06910 | 0.749634899 | | LOC_Os06g46890 | LOC_Os02g06584 | 0.502525704 | | LOC_Os06g47590 | LOC_Os02g06330 | 0.637640867 | | LOC_Os06g48290 | LOC_Os02g05640 | 0.60209635 | | LOC_Os06g48534 | LOC_Os02g05510 | 0.409149246 | | LOC_Os06g48610 | LOC_Os02g05470 | 0.183614136 | | LOC_Os06g48950 | LOC_Os02g04810 | 0.47727921 | | LOC_Os06g49010 | LOC_Os02g04680 | 0.854469375 | | LOC_Os06g49130 | LOC_Os02g04490 | 0.01183488 | | LOC_Os06g51220 | LOC_Os02g44930 | -0.252833384 | | LOC_Os07g01820 | LOC_Os03g54160 | 0.805167283 | | LOC_Os07g02060 | LOC_Os03g55080 | 0.461597868 | | LOC_Os07g02540 | LOC_Os03g55530 | 0.201685658 | | LOC_Os07g02800 | LOC_Os03g55590 | 0.562285113 | | LOC_Os07g03220 | LOC_Os03g56010 | 0.413712816 | | LOC_Os07g03250 | LOC_Os03g56050 | 0.682203206 | | LOC_Os07g04560 | LOC_Os03g56580 | 0.439484735 | | LOC_Os07g05010 | LOC_Os03g56950 | 0.726281702 | | LOC_Os07g05720 | LOC_Os03g57190 | 0.697692628 | | LOC_Os07g06470 | LOC_Os03g44540 | 0.356954996 | | LOC_Os07g06620 | LOC_Os03g44710 | 0.856790356 | | LOC_Os07g08140 | LOC_Os03g58160 | -0.04634273 | | LOC_Os07g08420 | LOC_Os03g58250 | 0.122847792 | | LOC_Os07g08460 | LOC_Os03g58350 | 0.282182141 | | LOC_Os07g08530 | LOC_Os07g08540 | 0.070712977 | | LOC_Os07g08540 | LOC_Os07g08600 | 0.107479715 | | LOC_Os07g08600 | LOC_Os07g08540 | 0.107479715 | | LOC_Os07g08880 | LOC_Os03g58530 | -0.11909254 | | LOC_Os07g09320 | LOC_Os03g58640 | 0.187889858 | | LOC_Os07g09740 | LOC_Os07g09830 | 0.228157877 | | LOC_Os07g09830 | LOC_Os07g09860 | 0.188710007 | | LOC_Os07g09860 | LOC_Os07g09830 | 0.188710007 | | LOC_Os07g10890 | LOC_Os03g59460 | -0.001542639 | | LOC_Os07g11010 | LOC_Os03g59530 | 0.641705128 | | LOC_Os07g12340 | LOC_Os03g60080 | 0.35841446 | | LOC_Os07g12510 | LOC_Os03g60120 | 0.570094054 | | LOC_Os07g13170 | LOC_Os03g60430 | -0.090560321 | | LOC_Os07g13260 | LOC_Os03g60630 | 0.126661813 | | LOC_Os07g22730 | LOC_Os07g22770 | 0.573901628 | | LOC_Os07g22770 | LOC_Os07g22730 | 0.573901628 | | LOC_Os07g36130 | LOC_Os07g36140 | -0.08864977 | | LOC_Os07g36140 | LOC_Os07g36130 | -0.08864977 | | LOC_Os07g40780 | LOC_Os03g31240 | 0.65082444 | | LOC_Os07g41580 | LOC_Os03g29970 | 0.672627343 | | LOC_Os07g41720 | LOC_Os03g29760 | 0.70508518 | | LOC_Os07g42370 | LOC_Os03g08320 | 0.403151516 | | LOC_Os07g42510 | LOC_Os03g08470 | 0.496245601 | | LOC_Os07g43030 | LOC_Os03g09100 | 0.340027102 | | LOC_Os07g43530 | LOC_Os03g26210 | 0.11339705 | | LOC_Os07g43580 | LOC_Os03g26130 | 0.088173124 | | LOC_Os07g44090 | LOC_Os03g25550 | 0.34088415 | | LOC_Os07g44690 | LOC_Os03g25120 | 0.247088717 | | LOC_Os07g47110 | LOC_Os03g22800 | 0.203247097 | | LOC_Os07g47140 | LOC_Os03g22770 | 0.275070181 | | LOC_Os07g47790 | LOC_Os03g22170 | 0.411220585 | | LOC_Os07g48150 | LOC_Os03g21870 | 0.452122479 | | LOC_Os07g48180 | LOC_Os03g21800 | 0.681694011 | | LOC_Os07g48260 | LOC_Os03g21710 | 0.422975786 | | LOC_Os07g48410 | LOC_Os03g21140 | 0.707152442 | | LOC_Os07g48450 | LOC_Os07g48550 | 0.608547258 | | LOC_Os07g48550 | LOC_Os07g48450 | 0.608547258 | | LOC_Os07g48560 | LOC_Os03g20910 | 0.764643256 | | LOC_Os07g48596 | LOC_Os03g20900 | 0.76459791 | | LOC_Os07g48630 | LOC_Os03g20790 | 0.695448639 | | LOC_Os07g48870 | LOC_Os03g20090 | 0.34733212 | | LOC_Os07g49030 | LOC_Os03g19020 | 0.387921457 | | LOC_Os07g49460 | LOC_Os03g17570 | 0.707772471 | | LOC_Os08g01190 | LOC_Os04g58820 | 0.371690265 | | LOC_Os08g01290 | LOC_Os04g59130 | 0.607405831 | | LOC_Os08g01330 | LOC_Os04g59470 | -0.044436909 | | LOC_Os08g03500 | LOC_Os08g03510 | 0.430698354 | | LOC_Os08g03510 | LOC_Os08g03500 | 0.430698354 | | LOC_Os08g07970 | LOC_Os04g54474 | 0.730028562 | | LOC_Os08g08220 | LOC_Os04g53720 | 0.313777376 | | LOC_Os08g10080 | LOC_Os04g52810 | -0.153969646 | | LOC_Os08g13060 | LOC_Os08g13070 | 0.847623108 | | LOC_Os08g13070 | LOC_Os08g13060 | 0.847623108 | | LOC_Os08g13840 | LOC_Os04g51560 | 0.59468987 | | LOC_Os08g23470 | LOC_Os08g23570 | 0.419308446 | | LOC_Os08g23570 | LOC_Os08g23470 | 0.419308446 | | LOC_Os08g25799 | LOC_Os09g12750 | 0.643681117 | | LOC_Os08g25820 | LOC_Os09g12770 | -0.288808588 | | LOC_Os08g29660 | LOC_Os09g16510 | -0.029025199 | | LOC_Os08g31080 | LOC_Os09g19950 | 0.922477747 | | LOC_Os08g31580 | LOC_Os09g20350 | 0.850199893 | | LOC_Os08g32080 | LOC_Os09g21180 | 0.364594861 | | LOC_Os08g32620 | LOC_Os09g21770 | 0.684044033 | | LOC_Os08g33050 | LOC_Os09g23200 | 0.476193676 | | LOC_Os08g33150 | LOC_Os09g23620 | 0.096364119 | | LOC_Os08g33160 | LOC_Os09g23660 | 0.612917967 | | LOC_Os08g33488 | LOC_Os02g36924 | 0.380461663 | | LOC_Os08g33530 | LOC_Os09g24480 | 0.524726227 | | LOC_Os08g33660 | LOC_Os02g36890 | 0.816151296 | | LOC_Os08g33670 | LOC_Os09g24560 | -0.201792561 | | LOC_Os08g34010 | LOC_Os09g24820 | 0.754950766 | | LOC_Os08g34360 | LOC_Os09g25600 | 0.187982584 | | LOC_Os08g36390 | LOC_Os09g27650 | 0.635470346 | | LOC_Os08g36450 | LOC_Os09g27850 | 0.522249738 | | LOC_Os08g36700 | LOC_Os09g28200 | 0.284029131 | | LOC_Os08g36740 | LOC_Os09g28210 | 0.395554271 | | LOC_Os08g36790 | LOC_Os09g28310 | 0.281639203 | | LOC_Os08g36920 | LOC_Os09g28440 | 0.725602653 | | LOC_Os08g37400 | LOC_Os09g29130 | 0.476850663 | | LOC_Os08g37580 | LOC_Os09g29460 | 0.705130569 | | LOC_Os08g37730 | LOC_Os09g29360 | 0.462127671 | | LOC_Os08g37904 | LOC_Os08g37920 | 0.635978372 | | LOC_Os08g37920 | LOC_Os08g37904 | 0.635978372 | | LOC_Os08g38020 | LOC_Os09g29820 | 0.478916758 | | LOC_Os08g38080 | LOC_Os09g29830 | 0.36350845 | | LOC_Os08g38210 | LOC_Os09g29930 | 0.755798323 | | LOC_Os08g38220 | LOC_Os09g29960 | 0.748691378 | | LOC_Os08g38780 | LOC_Os09g30310 | -0.074446287 | | LOC_Os08g38990 | LOC_Os09g30400 | 0.189003599 | | LOC_Os08g39390 | LOC_Os09g31140 | 0.37391525 | | LOC_Os08g39630 | LOC_Os09g31300 | 0.530684211 | | LOC_Os08g39830 | LOC_Os09g31400 | 0.210369535 | | LOC_Os08g39890 | LOC_Os09g31438 | 0.801762879 | | LOC_Os08g39980 | LOC_Os09g31454 | -0.19623157 | | LOC_Os08g40460 | LOC_Os08g40490 | 0.060573819 | | LOC_Os08g40490 | LOC_Os08g40460 | 0.060573819 | | LOC_Os08g41150 | LOC_Os08g41170 | 0.123260775 | | LOC_Os08g41230 | LOC_Os08g41240 | 0.337689426 | | LOC_Os08g41240 | LOC_Os08g41230 | 0.337689426 | | LOC_Os08g41320 | LOC_Os09g32510 | 0.468733503 | | LOC_Os08g41950 | LOC_Os08g41960 | 0.494656431 | | LOC_Os08g41960 | LOC_Os08g41950 | 0.494656431 | | LOC_Os08g42400 | LOC_Os09g33490 | 0.677402615 | | LOC_Os08g42440 | LOC_Os09g33550 | 0.690890543 | | LOC_Os08g42470 | LOC_Os09g33580 | 0.533919053 | | LOC_Os08g43090 | LOC_Os09g34880 | 0.202647485 | | LOC_Os08g43160 | LOC_Os09g34950 | -0.220022705 | | LOC_Os08g43210 | LOC_Os09g35030 | 0.381506544 | | LOC_Os08g43334 | LOC_Os09g35790 | 0.716851759 | | LOC_Os08g43550 | LOC_Os09g36730 | 0.17548207 | | LOC_Os08g44050 | LOC_Os09g38340 | 0.589994182 | | LOC_Os08g44940 | LOC_Os02g57490 | 0.809868597 | | LOC_Os08g44960 | LOC_Os09g39810 | 0.563880517 | | LOC_Os09g11460 | LOC_Os09g11480 | 0.618553603 | | LOC_Os09g11480 | LOC_Os09g11460 | 0.618553603 | | LOC_Os09g12750 | LOC_Os08g25799 | 0.643681117 | | LOC_Os09g12770 | LOC_Os08g25820 | -0.288808588 | | LOC_Os09g16510 | LOC_Os08g29660 | -0.029025199 | | LOC_Os09g19830 | LOC_Os08g30910 | -0.062765868 | | LOC_Os09g19950 | LOC_Os08g31080 | 0.922477747 | | LOC_Os09g20350 | LOC_Os08g31580 | 0.850199893 | | LOC_Os09g21180 | LOC_Os08g32080 | 0.364594861 | | LOC_Os09g21770 | LOC_Os08g32620 | 0.684044033 | | LOC_Os09g23200 | LOC_Os08g33050 | 0.476193676 | | LOC_Os09g23620 | LOC_Os08g33150 | 0.096364119 | | LOC_Os09g23660 | LOC_Os08g33160 | 0.612917967 | | LOC_Os09g24480 | LOC_Os08g33530 | 0.524726227 | | LOC_Os09g24560 | LOC_Os08g33670 | -0.201792561 | | LOC_Os09g24810 | LOC_Os09g24820 | 0.443198317 | | LOC_Os09g24820 | LOC_Os09g24810 | 0.443198317 | | LOC_Os09g25600 | LOC_Os08g34360 | 0.187982584 | | LOC_Os09g26170 | LOC_Os02g54520 | 0.624547288 | | LOC_Os09g26420 | LOC_Os02g54160 | 0.266069698 | | LOC_Os09g27650 | LOC_Os08g36390 | 0.635470346 | | LOC_Os09g27850 | LOC_Os08g36450 | 0.522249738 | | LOC_Os09g28200 | LOC_Os08g36700 | 0.284029131 | | LOC_Os09g28210 | LOC_Os06g10820 | 0.165516187 | | LOC_Os09g28310 | LOC_Os02g52780 | 0.534621351 | | LOC_Os09g28440 | LOC_Os02g52670 | 0.568146455 | | LOC_Os09g29130 | LOC_Os08g37400 | 0.476850663 | | LOC_Os09g29360 | LOC_Os02g52190 | 0.513011338 | | LOC_Os09g29460 | LOC_Os03g08960 | 0.433524417 | | LOC_Os09g29820 | LOC_Os02g49560 | 0.271013958 | | LOC_Os09g29830 | LOC_Os08g38080 | 0.36350845 | | LOC_Os09g29930 | LOC_Os02g49480 | 0.246772208 | | LOC_Os09g29960 | LOC_Os02g49440 | 0.586013276 | | LOC_Os09g30310 | LOC_Os08g38780 | -0.074446287 | | LOC_Os09g30400 | LOC_Os08g38990 | 0.189003599 | | LOC_Os09g31140 | LOC_Os08g39390 | 0.37391525 | | LOC_Os09g31300 | LOC_Os08g39630 | 0.530684211 | | LOC_Os09g31310 | LOC_Os02g48650 | 0.355047198 | | LOC_Os09g31400 | LOC_Os08g39830 | 0.210369535 | | LOC_Os09g31438 | LOC_Os08g39890 | 0.801762879 | | LOC_Os09g31454 | LOC_Os08g39980 | -0.19623157 | | LOC_Os09g32510 | LOC_Os01g68700 | 0.609186513 | | LOC_Os09g32948 | LOC_Os01g69850 | 0.079203687 | | LOC_Os09g33490 | LOC_Os01g70110 | 0.078781697 | | LOC_Os09g33550 | LOC_Os08g42440 | 0.690890543 | | LOC_Os09g33580 | LOC_Os08g42470 | 0.533919053 | | LOC_Os09g34880 | LOC_Os08g43090 | 0.202647485 | | LOC_Os09g34950 | LOC_Os08g43160 | -0.220022705 | | LOC_Os09g35010 | LOC_Os04g48350 | 0.804096918 | | LOC_Os09g35030 | LOC_Os08g43210 | 0.381506544 | | LOC_Os09g35760 | LOC_Os04g48070 | 0.043159684 | | LOC_Os09g35790 | LOC_Os04g48030 | 0.645357912 | | LOC_Os09g36730 | LOC_Os08g43550 | 0.17548207 | | LOC_Os09g38000 | LOC_Os09g38010 | 0.599552105 | | LOC_Os09g38010 | LOC_Os09g38000 | 0.599552105 | | LOC_Os09g38340 | LOC_Os08g44050 | 0.589994182 | | LOC_Os09g39810 | LOC_Os08g44960 | 0.563880517 | | LOC_Os10g22430 | LOC_Os03g09280 | 0.489385012 | | LOC_Os10g22600 | LOC_Os03g09170 | 0.279072827 | | LOC_Os10g22950 | LOC_Os03g09100 | 0.445339489 | | LOC_Os10g23090 | LOC_Os03g08960 | 0.268176725 | | LOC_Os10g25170 | LOC_Os03g08470 | 0.471151168 | | LOC_Os10g25230 | LOC_Os10g25290 | 0.730071946 | | LOC_Os10g25290 | LOC_Os10g25230 | 0.730071946 | | LOC_Os10g25850 | LOC_Os03g07880 | 0.224173872 | | LOC_Os10g26500 | LOC_Os03g07450 | 0.858512675 | | LOC_Os10g26620 | LOC_Os03g07360 | 0.795569651 | | LOC_Os10g27360 | LOC_Os10g27390 | -0.021577303 | | LOC_Os10g27390 | LOC_Os10g27360 | -0.021577303 | | LOC_Os10g28340 | LOC_Os03g06630 | 0.550880613 | | LOC_Os10g28870 | LOC_Os10g28970 | 0.620908287 | | LOC_Os10g28970 | LOC_Os10g28870 | 0.620908287 | | LOC_Os10g29610 | LOC_Os03g06350 | -0.106772998 | | LOC_Os10g30420 | LOC_Os03g05760 | 0.214012305 | | LOC_Os10g32070 | LOC_Os03g05160 | -0.03437087 | | LOC_Os10g32900 | LOC_Os03g04620 | 0.745587495 | | LOC_Os10g33760 | LOC_Os02g41450 | 0.567882425 | | LOC_Os10g33810 | LOC_Os02g41510 | 0.386694567 | | LOC_Os10g35930 | LOC_Os02g42820 | 0.750235088 | | LOC_Os10g36420 | LOC_Os02g42950 | 0.712893346 | | LOC_Os10g36810 | LOC_Os03g02160 | -0.036534791 | | LOC_Os10g37240 | LOC_Os02g43300 | 0.282138611 | | LOC_Os10g38000 | LOC_Os02g45420 | 0.520302113 | | LOC_Os10g38820 | LOC_Os03g03550 | 0.493372607 | | LOC_Os10g38834 | LOC_Os03g03540 | 0.316226507 | | LOC_Os10g38880 | LOC_Os02g45620 | 0.635697081 | | LOC_Os10g39030 | LOC_Os03g03260 | 0.85328421 | | LOC_Os10g39130 | LOC_Os02g45770 | 0.191151649 | | LOC_Os10g39190 | LOC_Os02g45850 | 0.398790393 | | LOC_Os10g39550 | LOC_Os03g03760 | 0.259387321 | | LOC_Os10g39750 | LOC_Os03g04310 | 0.574352712 | | LOC_Os10g40390 | LOC_Os02g44360 | 0.684942787 | | LOC_Os10g41130 | LOC_Os02g43940 | 0.341174482 | | LOC_Os10g41230 | LOC_Os04g46350 | 0.305697303 | | LOC_Os10g42690 | LOC_Os02g46930 | 0.787911579 | | LOC_Os10g42850 | LOC_Os02g47060 | 0.185461982 | | LOC_Os11g01480 | LOC_Os12g01490 | 0.937437413 | | LOC_Os11g02470 | LOC_Os05g40070 | 0.326536189 | | LOC_Os11g02480 | LOC_Os05g40080 | 0.236281488 | | LOC_Os11g03300 | LOC_Os11g03370 | 0.477741803 | | LOC_Os11g03370 | LOC_Os11g03300 | 0.477741803 | | LOC_Os11g03390 | LOC_Os12g03070 | 0.964143087 | | LOC_Os11g03440 | LOC_Os12g03150 | 0.403954569 | | LOC_Os11g04600 | LOC_Os12g04410 | 0.300232037 | | LOC_Os11g05614 | LOC_Os12g05990 | 0.258353347 | | LOC_Os11g05740 | LOC_Os12g06080 | 0.612304419 | | LOC_Os11g05770 | LOC_Os04g33870 | 0.503217382 | | LOC_Os11g06130 | LOC_Os12g06480 | 0.275132522 | | LOC_Os11g06170 | LOC_Os12g06520 | 0.722332236 | | LOC_Os11g06180 | LOC_Os12g06540 | 0.06234734 | | LOC_Os11g08080 | LOC_Os12g07730 | 0.367855088 | | LOC_Os11g09990 | LOC_Os11g10000 | 0.729777868 | | LOC_Os11g10000 | LOC_Os11g10040 | 0.756403372 | | LOC_Os11g10040 | LOC_Os11g10000 | 0.756403372 | | LOC_Os11g31330 | LOC_Os11g31380 | 0.877400823 | | LOC_Os11g31340 | LOC_Os11g31360 | 0.686365065 | | LOC_Os11g31380 | LOC_Os11g31330 | 0.877400823 | | LOC_Os11g45740 | LOC_Os12g37690 | 0.701488383 | | LOC_Os11g47460 | LOC_Os12g37970 | 0.710967661 | | LOC_Os12g01490 | LOC_Os11g01480 | 0.937437413 | | LOC_Os12g03070 | LOC_Os11g03390 | 0.964143087 | | LOC_Os12g03150 | LOC_Os11g03440 | 0.403954569 | | LOC_Os12g04410 | LOC_Os11g04600 | 0.300232037 | | LOC_Os12g05990 | LOC_Os01g64310 | 0.289870728 | | LOC_Os12g06080 | LOC_Os11g05740 | 0.612304419 | | LOC_Os12g06150 | LOC_Os04g33870 | 0.745583446 | | LOC_Os12g06200 | LOC_Os02g33430 | 0.477798066 | | LOC_Os12g06480 | LOC_Os11g06130 | 0.275132522 | | LOC_Os12g06520 | LOC_Os11g06170 | 0.722332236 | | LOC_Os12g06540 | LOC_Os11g06180 | 0.06234734 | | LOC_Os12g06630 | LOC_Os01g64700 | 0.388435524 | | LOC_Os12g07730 | LOC_Os11g08080 | 0.367855088 | | LOC_Os12g37690 | LOC_Os11g45740 | 0.701488383 | | LOC_Os12g37970 | LOC_Os11g47460 | 0.710967661 | | LOC_Os12g39850 | LOC_Os03g42100 | 0.356790469 | | LOC_Os12g40070 | LOC_Os12g40080 | 0.575982097 | | LOC_Os12g40080 | LOC_Os12g40070 | 0.575982097 | | LOC_Os12g40490 | LOC_Os03g42820 | 0.806891082 | | LOC_Os12g40710 | LOC_Os12g40730 | 0.383246874 | | LOC_Os12g40730 | LOC_Os12g40710 | 0.383246874 | | LOC_Os12g40890 | LOC_Os03g43400 | 0.665846834 | | LOC_Os12g40900 | LOC_Os03g43410 | 0.870969454 | | LOC_Os12g41230 | LOC_Os03g43730 | 0.464723604 | | LOC_Os12g41650 | LOC_Os03g43810 | 0.783866829 | | LOC_Os12g41860 | LOC_Os03g43930 | 0.839258454 | | LOC_Os12g42400 | LOC_Os03g44540 | 0.474487603 | | LOC_Os12g42610 | LOC_Os03g44710 | 0.881563797 | | LOC_Os12g43620 | LOC_Os03g46860 | 0.318507629 | | LOC_Os12g43950 | LOC_Os03g47740 | 0.734235225 | | LOC_Os03g42410 |  | singletons | | LOC_Os04g28000 |  | singletons | | LOC_Os06g42630 |  | singletons | | LOC_Os02g38470 |  | singletons | | LOC_Os01g67830 |  | singletons | | LOC_Os03g11370 |  | singletons | | LOC_Os06g02230 |  | singletons | | LOC_Os10g17630 |  | singletons | | LOC_Os07g12820 |  | singletons | | LOC_Os01g51610 |  | singletons | | LOC_Os01g13300 |  | singletons | | LOC_Os07g17230 |  | singletons | | LOC_Os08g06120 |  | singletons | | LOC_Os06g01860 |  | singletons | | LOC_Os07g37610 |  | singletons | | LOC_Os07g48200 |  | singletons | | LOC_Os01g68370 |  | singletons | | LOC_Os08g01090 |  | singletons | | LOC_Os03g06850 |  | singletons | | LOC_Os06g09420 |  | singletons | | LOC_Os11g09160 |  | singletons | | LOC_Os11g14010 |  | singletons | | LOC_Os07g41740 |  | singletons | | LOC_Os08g35240 |  | singletons | | LOC_Os06g09717 |  | singletons | | LOC_Os06g11940 |  | singletons | | LOC_Os06g09790 |  | singletons | | LOC_Os06g10780 |  | singletons | | LOC_Os03g15660 |  | singletons | | LOC_Os06g03670 |  | singletons | | LOC_Os01g73770 |  | singletons | | LOC_Os05g39590 |  | singletons | | LOC_Os03g07830 |  | singletons | | LOC_Os05g27930 |  | singletons | | LOC_Os08g45110 |  | singletons | | LOC_Os01g07120 |  | singletons | | LOC_Os05g28350 |  | singletons | | LOC_Os04g52090 |  | singletons | | LOC_Os06g42990 |  | singletons | | LOC_Os07g47330 |  | singletons | | LOC_Os08g07700 |  | singletons | | LOC_Os05g25260 |  | singletons | | LOC_Os06g06540 |  | singletons | | LOC_Os01g12440 |  | singletons | | LOC_Os01g46870 |  | singletons | | LOC_Os12g39330 |  | singletons | | LOC_Os07g38750 |  | singletons | | LOC_Os07g10410 |  | singletons | | LOC_Os12g41060 |  | singletons | | LOC_Os08g27220 |  | singletons | | LOC_Os04g18650 |  | singletons | | LOC_Os02g38090 |  | singletons | | LOC_Os03g64260 |  | singletons | | LOC_Os09g39850 |  | singletons | | LOC_Os05g37640 |  | singletons | | LOC_Os10g41330 |  | singletons | | LOC_Os04g46240 |  | singletons | | LOC_Os08g42550 |  | singletons | | LOC_Os11g06770 |  | singletons | | LOC_Os06g43220 |  | singletons | | LOC_Os05g03040 |  | singletons | | LOC_Os06g05340 |  | singletons | | LOC_Os05g45954 |  | singletons | | LOC_Os05g32270 |  | singletons | | LOC_Os11g19060 |  | singletons | | LOC_Os01g67410 |  | singletons | | LOC_Os04g55970 |  | singletons | | LOC_Os03g07940 |  | singletons | | LOC_Os03g12950 |  | singletons | | LOC_Os03g19900 |  | singletons | | LOC_Os08g07440 |  | singletons | | LOC_Os02g51300 |  | singletons | | LOC_Os10g26590 |  | singletons | | LOC_Os03g06920 |  | singletons | | LOC_Os02g29550 |  | singletons | | LOC_Os08g41030 |  | singletons | | LOC_Os09g13940 |  | singletons | | LOC_Os01g04020 |  | singletons | | LOC_Os12g29520 |  | singletons | | LOC_Os11g32110 |  | singletons | | LOC_Os01g70270 |  | singletons | | LOC_Os01g13520 |  | singletons | | LOC_Os06g47150 |  | singletons | | LOC_Os04g59430 |  | singletons | | LOC_Os07g08520 |  | singletons | | LOC_Os04g56850 |  | singletons | | LOC_Os12g41950 |  | singletons | | LOC_Os06g09660 |  | singletons | | LOC_Os08g40900 |  | singletons | | LOC_Os05g32890 |  | singletons | | LOC_Os05g32880 |  | singletons | | LOC_Os01g67770 |  | singletons | | LOC_Os03g12350 |  | singletons | | LOC_Os06g04010 |  | singletons | | LOC_Os07g39220 |  | singletons | | LOC_Os02g03690 |  | singletons | | LOC_Os01g08180 |  | singletons | | LOC_Os01g18870 |  | singletons | | LOC_Os04g51070 |  | singletons | | LOC_Os03g53020 |  | singletons | | LOC_Os04g31290 |  | singletons | | LOC_Os12g40630 |  | singletons | | LOC_Os09g25040 |  | singletons | | LOC_Os02g35660 |  | singletons | | LOC_Os06g09370 |  | singletons | | LOC_Os05g01256 |  | singletons | | LOC_Os03g12940 |  | singletons | | LOC_Os03g51910 |  | singletons | | LOC_Os06g16400 |  | singletons | | LOC_Os04g28280 |  | singletons | | LOC_Os02g47660 |  | singletons | | LOC_Os12g32400 |  | singletons | | LOC_Os11g41640 |  | singletons | | LOC_Os03g55550 |  | singletons | | LOC_Os07g39940 |  | singletons | | LOC_Os03g10770 |  | singletons | | LOC_Os06g30090 |  | singletons | | LOC_Os02g48060 |  | singletons | | LOC_Os01g02110 |  | singletons | | LOC_Os03g39432 |  | singletons | | LOC_Os01g61480 |  | singletons | | LOC_Os08g01700 |  | singletons | | LOC_Os01g38610 |  | singletons | | LOC_Os01g67480 |  | singletons | | LOC_Os04g52770 |  | singletons | | LOC_Os10g40740 |  | singletons | | LOC_Os03g17130 |  | singletons | | LOC_Os01g11910 |  | singletons | | LOC_Os03g59670 |  | singletons | | LOC_Os03g15440 |  | singletons | | LOC_Os03g51580 |  | singletons | | LOC_Os03g46790 |  | singletons | | LOC_Os03g03000 |  | singletons | | LOC_Os05g50900 |  | singletons | | LOC_Os10g23050 |  | singletons | | LOC_Os05g51820 |  | singletons | | LOC_Os04g47080 |  | singletons | | LOC_Os10g42430 |  | singletons | | LOC_Os01g50940 |  | singletons | | LOC_Os01g13460 |  | singletons | | LOC_Os02g23823 |  | singletons | | LOC_Os01g39330 |  | singletons | | LOC_Os02g13670 |  | singletons | | LOC_Os10g01530 |  | singletons | | LOC_Os03g12760 |  | singletons | | LOC_Os12g31430 |  | singletons | | LOC_Os12g40590 |  | singletons | | LOC_Os02g02820 |  | singletons | | LOC_Os07g36460 |  | singletons | | LOC_Os01g72370 |  | singletons | | LOC_Os04g23550 |  | singletons | | LOC_Os04g23440 |  | singletons | | LOC_Os11g32100 |  | singletons | | LOC_Os01g70310 |  | singletons | | LOC_Os05g38140 |  | singletons | | LOC_Os08g04390 |  | singletons | | LOC_Os07g35870 |  | singletons | | LOC_Os11g39000 |  | singletons | | LOC_Os03g55220 |  | singletons | | LOC_Os04g56654 |  | singletons | | LOC_Os02g49450 |  | singletons | | LOC_Os07g45310 |  | singletons | | LOC_Os01g16670 |  | singletons | | LOC_Os11g35320 |  | singletons | | LOC_Os08g25060 |  | singletons | | LOC_Os06g07820 |  | singletons | | LOC_Os03g52000 |  | singletons | | LOC_Os06g41770 |  | singletons | | LOC_Os11g05640 |  | singletons | | LOC_Os09g31390 |  | singletons | | LOC_Os09g10840 |  | singletons | | LOC_Os06g41100 |  | singletons | | LOC_Os02g10140 |  | singletons | | LOC_Os01g17260 |  | singletons | | LOC_Os06g15480 |  | singletons | | LOC_Os06g50310 |  | singletons | | LOC_Os07g44950 |  | singletons | | LOC_Os05g34050 |  | singletons | | LOC_Os01g07880 |  | singletons | | LOC_Os09g34060 |  | singletons | | LOC_Os12g09250 |  | singletons | | LOC_Os11g11100 |  | singletons | | LOC_Os01g64000 |  | singletons | | LOC_Os02g58670 |  | singletons | | LOC_Os06g50830 |  | singletons | | LOC_Os03g19370 |  | singletons | | LOC_Os12g37410 |  | singletons | | LOC_Os05g03860 |  | singletons | | LOC_Os02g03960 |  | singletons | | LOC_Os08g26880 |  | singletons | | LOC_Os12g40920 |  | singletons | | LOC_Os02g16680 |  | singletons | | LOC_Os03g13614 |  | singletons | | LOC_Os12g13170 |  | singletons | | LOC_Os02g03580 |  | singletons | | LOC_Os03g50310 |  | singletons | | LOC_Os08g15050 |  | singletons | | LOC_Os06g16370 |  | singletons | | LOC_Os09g06464 |  | singletons | | LOC_Os06g01340 |  | singletons | | LOC_Os02g47810 |  | singletons | | LOC_Os01g17000 |  | singletons | | LOC_Os07g48570 |  | singletons | | LOC_Os01g15900 |  | singletons | | LOC_Os02g15350 |  | singletons | | LOC_Os04g58190 |  | singletons | | LOC_Os12g38200 |  | singletons | | LOC_Os03g55610 |  | singletons | | LOC_Os07g32510 |  | singletons | | LOC_Os03g38870 |  | singletons | | LOC_Os10g35300 |  | singletons | | LOC_Os03g47970 |  | singletons | | LOC_Os03g52450 |  | singletons | | LOC_Os12g42970 |  | singletons | | LOC_Os02g56250 |  | singletons | | LOC_Os11g08410 |  | singletons | | LOC_Os03g03850 |  | singletons | | LOC_Os10g40810 |  | singletons | | LOC_Os04g46020 |  | singletons | | LOC_Os03g61570 |  | singletons | | LOC_Os01g74540 |  | singletons | | LOC_Os12g07120 |  | singletons | | LOC_Os03g11600 |  | singletons | | LOC_Os12g38940 |  | singletons | | LOC_Os05g51830 |  | singletons | | LOC_Os01g68160 |  | singletons | | LOC_Os03g05480 |  | singletons | | LOC_Os03g05690 |  | singletons | | LOC_Os07g38240 |  | singletons | | LOC_Os03g40710 |  | singletons | | LOC_Os04g39520 |  | singletons | | LOC_Os07g39970 |  | singletons | | LOC_Os07g39960 |  | singletons | | LOC_Os03g55540 |  | singletons | | LOC_Os07g40080 |  | singletons | | LOC_Os03g32230 |  | singletons | | LOC_Os03g32220 |  | singletons | | LOC_Os06g07020 |  | singletons | | LOC_Os04g08060 |  | singletons | | LOC_Os02g57790 |  | singletons | | LOC_Os11g47630 |  | singletons | | LOC_Os03g60570 |  | singletons | | LOC_Os03g60560 |  | singletons | | LOC_Os04g08600 |  | singletons | | LOC_Os02g01090 |  | singletons | | LOC_Os03g13600 |  | singletons | | LOC_Os09g26210 |  | singletons | | LOC_Os04g02510 |  | singletons | | LOC_Os11g06840 |  | singletons | | LOC_Os04g59380 |  | singletons | | LOC_Os01g57650 |  | singletons | | LOC_Os03g15790 |  | singletons | | LOC_Os06g20020 |  | singletons | | LOC_Os12g38960 |  | singletons | | LOC_Os06g51140 |  | singletons | | LOC_Os03g49132 |  | singletons | | LOC_Os11g25610 |  | singletons | | LOC_Os05g01550 |  | singletons | | LOC_Os02g34680 |  | singletons | | LOC_Os07g40950 |  | singletons | | LOC_Os01g66570 |  | singletons | | LOC_Os05g03020 |  | singletons | | LOC_Os02g02424 |  | singletons | | LOC_Os12g18150 |  | singletons | | LOC_Os01g67970 |  | singletons | | LOC_Os06g40960 |  | singletons | | LOC_Os09g13680 |  | singletons | | LOC_Os08g44830 |  | singletons | | LOC_Os04g08290 |  | singletons | | LOC_Os02g36360 |  | singletons | | LOC_Os12g07280 |  | singletons | | LOC_Os03g62230 |  | singletons | | LOC_Os01g65080 |  | singletons | | LOC_Os03g13400 |  | singletons | | LOC_Os02g31890 |  | singletons | | LOC_Os07g39310 |  | singletons | | LOC_Os03g10140 |  | singletons | | LOC_Os01g14010 |  | singletons | | LOC_Os10g28330 |  | singletons | | LOC_Os01g70870 |  | singletons | | LOC_Os01g09850 |  | singletons | | LOC_Os09g38790 |  | singletons | | LOC_Os07g23450 |  | singletons | | LOC_Os05g14130 |  | singletons | | LOC_Os11g48000 |  | singletons | | LOC_Os07g01180 |  | singletons | | LOC_Os05g20930 |  | singletons | | LOC_Os04g50070 |  | singletons | | LOC_Os09g39660 |  | singletons | | LOC_Os04g41060 |  | singletons | | LOC_Os05g03760 |  | singletons | | LOC_Os07g38090 |  | singletons | | LOC_Os12g33090 |  | singletons | | LOC_Os03g49170 |  | singletons | | LOC_Os01g14870 |  | singletons | | LOC_Os09g13530 |  | singletons | | LOC_Os06g43120 |  | singletons | | LOC_Os06g46400 |  | singletons | | LOC_Os08g04170 |  | singletons | | LOC_Os06g49080 |  | singletons | | LOC_Os07g04580 |  | singletons | | LOC_Os02g19804 |  | singletons | | LOC_Os04g35800 |  | singletons | | LOC_Os09g36090 |  | singletons | | LOC_Os04g32340 |  | singletons | | LOC_Os08g06330 |  | singletons | | LOC_Os05g08400 |  | singletons | | LOC_Os04g02730 |  | singletons | | LOC_Os10g25220 |  | singletons | | LOC_Os12g03554 |  | singletons | | LOC_Os01g39100 |  | singletons | | LOC_Os01g61830 |  | singletons | | LOC_Os02g55000 |  | singletons | | LOC_Os04g56750 |  | singletons | | LOC_Os08g38370 |  | singletons | | LOC_Os03g61110 |  | singletons | | LOC_Os05g41790 |  | singletons | | LOC_Os07g04650 |  | singletons | | LOC_Os01g07930 |  | singletons | | LOC_Os08g03310 |  | singletons | | LOC_Os07g39440 |  | singletons | | LOC_Os07g18050 |  | singletons | | LOC_Os06g07350 |  | singletons | | LOC_Os04g57600 |  | singletons | | LOC_Os02g58440 |  | singletons | | LOC_Os09g31482 |  | singletons | | LOC_Os02g35150 |  | singletons | | LOC_Os12g18120 |  | singletons | | LOC_Os06g32860 |  | singletons | | LOC_Os01g42970 |  | singletons | | LOC_Os12g21700 |  | singletons | | LOC_Os11g28270 |  | singletons | | LOC_Os01g68860 |  | singletons | | LOC_Os04g57010 |  | singletons | | LOC_Os03g18950 |  | singletons | | LOC_Os06g21390 |  | singletons | | LOC_Os09g19940 |  | singletons | | LOC_Os01g15300 |  | singletons | | LOC_Os07g30774 |  | singletons | | LOC_Os09g39490 |  | singletons | | LOC_Os01g08790 |  | singletons | | LOC_Os03g48970 |  | singletons | | LOC_Os12g41880 |  | singletons | | LOC_Os08g09690 |  | singletons | | LOC_Os02g53620 |  | singletons | | LOC_Os02g49410 |  | singletons | | LOC_Os08g07740 |  | singletons | | LOC_Os05g49780 |  | singletons | | LOC_Os06g17480 |  | singletons | | LOC_Os02g49370 |  | singletons | | LOC_Os08g29500 |  | singletons | | LOC_Os10g11580 |  | singletons | | LOC_Os05g23910 |  | singletons | | LOC_Os01g24460 |  | singletons | | LOC_Os01g39850 |  | singletons | | LOC_Os01g01290 |  | singletons | | LOC_Os03g14669 |  | singletons | | LOC_Os08g10560 |  | singletons | | LOC_Os11g34200 |  | singletons | | LOC_Os05g41450 |  | singletons | | LOC_Os12g34510 |  | singletons | | LOC_Os03g51200 |  | singletons | | LOC_Os12g25120 |  | singletons | | LOC_Os03g63530 |  | singletons | | LOC_Os08g28214 |  | singletons | | LOC_Os01g55580 |  | singletons | | LOC_Os06g22670 |  | singletons | | LOC_Os02g17460 |  | singletons | | LOC_Os04g09560 |  | singletons | | LOC_Os05g51040 |  | singletons | | LOC_Os07g07974 |  | singletons | | LOC_Os12g41210 |  | singletons | | LOC_Os05g43380 |  | singletons | | LOC_Os01g36570 |  | singletons | | LOC_Os02g02870 |  | singletons | | LOC_Os08g03520 |  | singletons | | LOC_Os06g39600 |  | singletons | | LOC_Os10g39540 |  | singletons | | LOC_Os01g48700 |  | singletons | | LOC_Os07g12210 |  | singletons | | LOC_Os08g27740 |  | singletons | | LOC_Os06g36970 |  | singletons | | LOC_Os01g71850 |  | singletons | | LOC_Os10g34884 |  | singletons | | LOC_Os08g20486 |  | singletons | | LOC_Os09g11390 |  | singletons | | LOC_Os05g40680 |  | singletons | | LOC_Os03g40160 |  | singletons | | LOC_Os02g01950 |  | singletons | | LOC_Os01g16740 |  | singletons | | LOC_Os06g13740 |  | singletons | | LOC_Os02g16140 |  | singletons | | LOC_Os05g25320 |  | singletons | | LOC_Os07g27800 |  | singletons | | LOC_Os07g27770 |  | singletons | | LOC_Os02g18370 |  | singletons | | LOC_Os08g32440 |  | singletons | | LOC_Os10g42160 |  | singletons | | LOC_Os02g25470 |  | singletons | | LOC_Os12g05940 |  | singletons | | LOC_Os12g03660 |  | singletons | | LOC_Os02g01860 |  | singletons | | LOC_Os01g23840 |  | singletons | | LOC_Os02g33750 |  | singletons | | LOC_Os01g63380 |  | singletons | | LOC_Os04g52560 |  | singletons | | LOC_Os03g56630 |  | singletons | | LOC_Os07g31400 |  | singletons | | LOC_Os07g37630 |  | singletons | | LOC_Os03g08370 |  | singletons | | LOC_Os07g42400 |  | singletons | | LOC_Os01g06852 |  | singletons | | LOC_Os09g14040 |  | singletons | | LOC_Os01g16660 |  | singletons | | LOC_Os05g39790 |  | singletons | | LOC_Os08g15510 |  | singletons | | LOC_Os08g14880 |  | singletons | | LOC_Os03g37920 |  | singletons | | LOC_Os03g21660 |  | singletons | | LOC_Os02g28180 |  | singletons | | LOC_Os02g10840 |  | singletons | | LOC_Os12g06380 |  | singletons | | LOC_Os06g07010 |  | singletons | | LOC_Os02g34590 |  | singletons | | LOC_Os11g02964 |  | singletons | | LOC_Os05g03800 |  | singletons | | LOC_Os05g06170 |  | singletons | | LOC_Os06g39090 |  | singletons | | LOC_Os10g14040 |  | singletons | | LOC_Os11g05340 |  | singletons | | LOC_Os03g06860 |  | singletons | | LOC_Os02g26780 |  | singletons | | LOC_Os02g39220 |  | singletons | | LOC_Os02g16240 |  | singletons | | LOC_Os01g32700 |  | singletons | | LOC_Os03g15040 |  | singletons | | LOC_Os06g25610 |  | singletons | | LOC_Os03g41800 |  | singletons | | LOC_Os11g19030 |  | singletons | | LOC_Os09g27390 |  | singletons | | LOC_Os05g30310 |  | singletons | | LOC_Os04g36590 |  | singletons | | LOC_Os02g31850 |  | singletons | | LOC_Os01g19050 |  | singletons | | LOC_Os03g45300 |  | singletons | | LOC_Os11g14020 |  | singletons | | LOC_Os06g49550 |  | singletons | | LOC_Os03g50900 |  | singletons | | LOC_Os06g39680 |  | singletons | | LOC_Os12g12380 |  | singletons | | LOC_Os11g45530 |  | singletons | | LOC_Os02g39540 |  | singletons | | LOC_Os03g62660 |  | singletons | | LOC_Os02g39520 |  | singletons | | LOC_Os04g40060 |  | singletons | | LOC_Os03g15010 |  | singletons | | LOC_Os07g31420 |  | singletons | | LOC_Os03g03560 |  | singletons | | LOC_Os11g07050 |  | singletons | | LOC_Os04g37619 |  | singletons | | LOC_Os03g51870 |  | singletons | | LOC_Os06g16430 |  | singletons | | LOC_Os08g23680 |  | singletons | | LOC_Os05g46780 |  | singletons | | LOC_Os11g27530 |  | singletons | | LOC_Os10g34580 |  | singletons | | LOC_Os02g22020 |  | singletons | | LOC_Os12g39640 |  | singletons | | LOC_Os01g08160 |  | singletons | | LOC_Os10g32600 |  | singletons | | LOC_Os03g45194 |  | singletons | | LOC_Os03g55760 |  | singletons | | LOC_Os04g56990 |  | singletons | | LOC_Os03g21240 |  | singletons | | LOC_Os07g25710 |  | singletons | | LOC_Os06g40710 |  | singletons | | LOC_Os05g41240 |  | singletons | | LOC_Os08g33750 |  | singletons | | LOC_Os08g06370 |  | singletons | | LOC_Os02g46940 |  | singletons | | LOC_Os01g62660 |  | singletons | | LOC_Os05g34110 |  | singletons | | LOC_Os01g74020 |  | singletons | | LOC_Os06g24070 |  | singletons | | LOC_Os01g13740 |  | singletons | | LOC_Os01g14720 |  | singletons | | LOC_Os03g50110 |  | singletons | | LOC_Os11g11600 |  | singletons | | LOC_Os03g15680 |  | singletons | | LOC_Os04g35250 |  | singletons | | LOC_Os03g51330 |  | singletons | | LOC_Os06g01620 |  | singletons | | LOC_Os04g50060 |  | singletons | | LOC_Os03g48450 |  | singletons | | LOC_Os01g65900 |  | singletons | | LOC_Os07g36170 |  | singletons | | LOC_Os07g39470 |  | singletons | | LOC_Os07g39820 |  | singletons | | LOC_Os03g31880 |  | singletons | | LOC_Os07g40020 |  | singletons | | LOC_Os05g42130 |  | singletons | | LOC_Os03g29480 |  | singletons | | LOC_Os11g31100 |  | singletons | | LOC_Os03g49990 |  | singletons | | LOC_Os06g10900 |  | singletons | | LOC_Os06g03710 |  | singletons | | LOC_Os07g38030 |  | singletons | | LOC_Os06g02560 |  | singletons | | LOC_Os07g28430 |  | singletons | | LOC_Os03g47140 |  | singletons | | LOC_Os03g51970 |  | singletons | | LOC_Os11g35030 |  | singletons | | LOC_Os12g29980 |  | singletons | | LOC_Os01g70810 |  | singletons | | LOC_Os06g39906 |  | singletons | | LOC_Os07g39320 |  | singletons | | LOC_Os03g10210 |  | singletons | | LOC_Os09g35910 |  | singletons | | LOC_Os02g49700 |  | singletons | | LOC_Os07g24350 |  | singletons | | LOC_Os10g01470 |  | singletons | | LOC_Os03g12860 |  | singletons | | LOC_Os02g35770 |  | singletons | | LOC_Os06g12400 |  | singletons | | LOC_Os02g05450 |  | singletons | | LOC_Os03g03164 |  | singletons | | LOC_Os08g19650 |  | singletons | | LOC_Os03g51710 |  | singletons | | LOC_Os03g51690 |  | singletons | | LOC_Os01g63510 |  | singletons | | LOC_Os04g56780 |  | singletons | | LOC_Os01g62310 |  | singletons | | LOC_Os08g14400 |  | singletons | | LOC_Os03g55990 |  | singletons | | LOC_Os10g33960 |  | singletons | | LOC_Os03g01890 |  | singletons | | LOC_Os04g55590 |  | singletons | | LOC_Os08g19590 |  | singletons | | LOC_Os01g55549 |  | singletons | | LOC_Os06g10600 |  | singletons | | LOC_Os10g42490 |  | singletons | | LOC_Os08g04190 |  | singletons | | LOC_Os01g60270 |  | singletons | | LOC_Os03g47730 |  | singletons | | LOC_Os06g01934 |  | singletons | | LOC_Os03g52239 |  | singletons | | LOC_Os03g06930 |  | singletons | | LOC_Os05g09630 |  | singletons | | LOC_Os06g29020 |  | singletons | | LOC_Os07g39800 |  | singletons | | LOC_Os03g63750 |  | singletons | | LOC_Os06g36930 |  | singletons | | LOC_Os01g39020 |  | singletons | | LOC_Os03g12370 |  | singletons | | LOC_Os09g28354 |  | singletons | | LOC_Os02g29340 |  | singletons | | LOC_Os05g45410 |  | singletons | | LOC_Os01g54550 |  | singletons | | LOC_Os01g53220 |  | singletons | | LOC_Os01g43590 |  | singletons | | LOC_Os02g32590 |  | singletons | | LOC_Os04g51000 |  | singletons | | LOC_Os03g15940 |  | singletons | | LOC_Os12g32620 |  | singletons | | LOC_Os06g13030 |  | singletons | | LOC_Os08g06659 |  | singletons | | LOC_Os01g14030 |  | singletons | | LOC_Os10g07510 |  | singletons | | LOC_Os03g14270 |  | singletons | | LOC_Os01g56530 |  | singletons | | LOC_Os05g27980 |  | singletons | | LOC_Os02g48270 |  | singletons | | LOC_Os03g45750 |  | singletons | | LOC_Os05g07270 |  | singletons | | LOC_Os01g60960 |  | singletons | | LOC_Os03g41600 |  | singletons | | LOC_Os01g07480 |  | singletons | | LOC_Os07g40000 |  | singletons | | LOC_Os03g41330 |  | singletons | | LOC_Os01g32770 |  | singletons | | LOC_Os01g03890 |  | singletons | | LOC_Os03g33090 |  | singletons | | LOC_Os07g04170 |  | singletons | | LOC_Os01g11510 |  | singletons | | LOC_Os08g38590 |  | singletons | | LOC_Os06g11970 |  | singletons | | LOC_Os11g43740 |  | singletons | | LOC_Os01g52680 |  | singletons | | LOC_Os08g02070 |  | singletons | | LOC_Os12g10520 |  | singletons | | LOC_Os04g52410 |  | singletons | | LOC_Os06g23950 |  | singletons | | LOC_Os06g49840 |  | singletons | | LOC_Os07g41370 |  | singletons | | LOC_Os12g31748 |  | singletons | | LOC_Os06g06750 |  | singletons | | LOC_Os03g11614 |  | singletons | | LOC_Os12g10540 |  | singletons | | LOC_Os01g66290 |  | singletons | | LOC_Os06g01890 |  | singletons | | LOC_Os03g08754 |  | singletons | | LOC_Os02g49840 |  | singletons | | LOC_Os04g23910 |  | singletons | | LOC_Os01g68560 |  | singletons | | LOC_Os01g23770 |  | singletons | | LOC_Os01g23760 |  | singletons | | LOC_Os01g67890 |  | singletons | | LOC_Os03g37670 |  | singletons | | LOC_Os01g18440 |  | singletons | | LOC_Os03g38610 |  | singletons | | LOC_Os01g18420 |  | singletons | | LOC_Os06g22760 |  | singletons | | LOC_Os05g23780 |  | singletons | | LOC_Os03g14850 |  | singletons | | LOC_Os02g06860 |  | singletons | | LOC_Os12g21880 |  | singletons | | LOC_Os12g21850 |  | singletons | | LOC_Os06g30830 |  | singletons | | LOC_Os06g30810 |  | singletons | | LOC_Os09g02780 |  | singletons | | LOC_Os07g39430 |  | singletons | | LOC_Os07g22670 |  | singletons | | LOC_Os02g39040 |  | singletons | | LOC_Os09g38720 |  | singletons | | LOC_Os11g14130 |  | singletons | | LOC_Os02g51460 |  | singletons | | LOC_Os06g12110 |  | singletons | | LOC_Os07g24090 |  | singletons | | LOC_Os05g34160 |  | singletons | | LOC_Os04g54510 |  | singletons | | LOC_Os07g04230 |  | singletons | | LOC_Os02g54200 |  | singletons | | LOC_Os03g24590 |  | singletons | | LOC_Os03g57149 |  | singletons | | LOC_Os08g40630 |  | singletons | | LOC_Os07g43420 |  | singletons | | LOC_Os05g03550 |  | singletons | | LOC_Os01g74410 |  | singletons | | LOC_Os03g04900 |  | singletons | | LOC_Os05g49310 |  | singletons | | LOC_Os01g45090 |  | singletons | | LOC_Os01g03720 |  | singletons | | LOC_Os03g19120 |  | singletons | | LOC_Os01g19970 |  | singletons | | LOC_Os10g35660 |  | singletons | | LOC_Os12g33070 |  | singletons | | LOC_Os03g56090 |  | singletons | | LOC_Os08g15020 |  | singletons | | LOC_Os01g52410 |  | singletons | | LOC_Os01g09590 |  | singletons | | LOC_Os04g39470 |  | singletons | | LOC_Os03g18480 |  | singletons | | LOC_Os11g35390 |  | singletons | | LOC_Os01g36460 |  | singletons | | LOC_Os08g05520 |  | singletons | | LOC_Os07g31470 |  | singletons | | LOC_Os01g51260 |  | singletons | | LOC_Os03g38210 |  | singletons | | LOC_Os01g07450 |  | singletons | | LOC_Os02g02370 |  | singletons | | LOC_Os08g37970 |  | singletons | | LOC_Os03g27090 |  | singletons | | LOC_Os06g02250 |  | singletons | | LOC_Os04g50680 |  | singletons | | LOC_Os07g37210 |  | singletons | | LOC_Os12g07640 |  | singletons | | LOC_Os01g50110 |  | singletons | | LOC_Os06g10350 |  | singletons | | LOC_Os03g29614 |  | singletons | | LOC_Os06g40330 |  | singletons | | LOC_Os06g46560 |  | singletons | | LOC_Os02g49250 |  | singletons | | LOC_Os12g38400 |  | singletons | | LOC_Os07g04700 |  | singletons | | LOC_Os01g04930 |  | singletons | | LOC_Os08g34960 |  | singletons | | LOC_Os03g13310 |  | singletons | | LOC_Os01g63160 |  | singletons | | LOC_Os09g01960 |  | singletons | | LOC_Os05g28320 |  | singletons | | LOC_Os01g74590 |  | singletons | | LOC_Os03g51110 |  | singletons | | LOC_Os11g10130 |  | singletons | | LOC_Os01g16810 |  | singletons | | LOC_Os01g63680 |  | singletons | | LOC_Os07g14110 |  | singletons | | LOC_Os07g12130 |  | singletons | | LOC_Os12g07610 |  | singletons | | LOC_Os06g06740 |  | singletons | | LOC_Os04g28090 |  | singletons | | LOC_Os12g13570 |  | singletons | | LOC_Os01g12860 |  | singletons | | LOC_Os06g08290 |  | singletons | | LOC_Os03g62379 |  | singletons | | LOC_Os04g30890 |  | singletons | | LOC_Os02g17190 |  | singletons | | LOC_Os06g19980 |  | singletons | | LOC_Os07g49530 |  | singletons | | LOC_Os05g46330 |  | singletons | | LOC_Os08g06240 |  | singletons | | LOC_Os05g02420 |  | singletons | | LOC_Os01g07430 |  | singletons | | LOC_Os06g14700 |  | singletons | | LOC_Os03g19630 |  | singletons | | LOC_Os01g11200 |  | singletons | | LOC_Os07g25370 |  | singletons | | LOC_Os10g20990 |  | singletons | | LOC_Os07g30130 |  | singletons | | LOC_Os03g31230 |  | singletons | | LOC_Os01g43230 |  | singletons | | LOC_Os01g43180 |  | singletons | | LOC_Os01g09760 |  | singletons | | LOC_Os02g10060 |  | singletons | | LOC_Os02g34630 |  | singletons | | LOC_Os04g40420 |  | singletons | | LOC_Os01g51154 |  | singletons | | LOC_Os12g41920 |  | singletons | | LOC_Os01g40670 |  | singletons | | LOC_Os03g56234 |  | singletons | | LOC_Os03g13790 |  | singletons | | LOC_Os03g53960 |  | singletons | | LOC_Os12g33950 |  | singletons | | LOC_Os02g47744 |  | singletons | | LOC_Os06g51260 |  | singletons | | LOC_Os08g06110 |  | singletons | | LOC_Os06g01670 |  | singletons | | LOC_Os02g45670 |  | singletons | | LOC_Os06g45840 |  | singletons | | LOC_Os03g62100 |  | singletons | | LOC_Os02g30700 |  | singletons | | LOC_Os04g27410 |  | singletons | | LOC_Os01g34060 |  | singletons | | LOC_Os04g58020 |  | singletons | | LOC_Os08g05510 |  | singletons | | LOC_Os10g41260 |  | singletons | | LOC_Os01g09280 |  | singletons | | LOC_Os08g04840 |  | singletons | | LOC_Os10g41200 |  | singletons | | LOC_Os03g43800 |  | singletons | | LOC_Os07g26150 |  | singletons | | LOC_Os04g01970 |  | singletons | | LOC_Os03g51220 |  | singletons | | LOC_Os02g57270 |  | singletons | | LOC_Os10g21560 |  | singletons | | LOC_Os03g12120 |  | singletons | | LOC_Os03g59730 |  | singletons | | LOC_Os06g36480 |  | singletons | | LOC_Os08g33910 |  | singletons | | LOC_Os12g43530 |  | singletons | | LOC_Os06g51070 |  | singletons | | LOC_Os03g01870 |  | singletons | | LOC_Os02g56600 |  | singletons | | LOC_Os12g41680 |  | singletons | | LOC_Os06g23650 |  | singletons | | LOC_Os09g32260 |  | singletons | | LOC_Os03g42630 |  | singletons | | LOC_Os11g03310 |  | singletons | | LOC_Os01g29840 |  | singletons | | LOC_Os08g40030 |  | singletons | | LOC_Os08g02300 |  | singletons | | LOC_Os06g04090 |  | singletons | | LOC_Os06g01480 |  | singletons | | LOC_Os01g15640 |  | singletons | | LOC_Os10g42130 |  | singletons | | LOC_Os03g02800 |  | singletons | | LOC_Os08g06140 |  | singletons | | LOC_Os06g01230 |  | singletons | | LOC_Os05g35170 |  | singletons | | LOC_Os07g37920 |  | singletons | | LOC_Os05g34310 |  | singletons | | LOC_Os02g12310 |  | singletons | | LOC_Os11g08210 |  | singletons | | LOC_Os12g29330 |  | singletons | | LOC_Os09g32040 |  | singletons | | LOC_Os08g23880 |  | singletons | | LOC_Os05g43960 |  | singletons | | LOC_Os01g59640 |  | singletons | | LOC_Os10g26270 |  | singletons | | LOC_Os12g07790 |  | singletons | | LOC_Os12g22940 |  | singletons | | LOC_Os12g23090 |  | singletons | | LOC_Os12g22630 |  | singletons | | LOC_Os07g17180 |  | singletons | | LOC_Os07g13920 |  | singletons | | LOC_Os11g04960 |  | singletons | | LOC_Os10g09820 |  | singletons | | LOC_Os09g12380 |  | singletons | | LOC_Os03g10150 |  | singletons | | LOC_Os01g40970 |  | singletons | | LOC_Os01g53160 |  | singletons | | LOC_Os01g43610 |  | singletons | | LOC_Os05g25910 |  | singletons | | LOC_Os01g12690 |  | singletons | | LOC_Os04g37510 |  | singletons | | LOC_Os10g41100 |  | singletons | | LOC_Os07g15770 |  | singletons | | LOC_Os05g50930 |  | singletons | | LOC_Os01g34610 |  | singletons | | LOC_Os12g16160 |  | singletons | | LOC_Os05g51690 |  | singletons | | LOC_Os09g35880 |  | singletons | | LOC_Os06g49880 |  | singletons | | LOC_Os12g10660 |  | singletons | | LOC_Os06g05890 |  | singletons | | LOC_Os02g39360 |  | singletons | | LOC_Os04g41560 |  | singletons | | LOC_Os10g30890 |  | singletons | | LOC_Os11g05930 |  | singletons | | LOC_Os03g16090 |  | singletons | | LOC_Os06g08400 |  | singletons | | LOC_Os07g15540 |  | singletons | | LOC_Os04g08740 |  | singletons | | LOC_Os02g57530 |  | singletons | | LOC_Os10g21810 |  | singletons | | LOC_Os01g69920 |  | singletons | | LOC_Os03g50860 |  | singletons | | LOC_Os02g50480 |  | singletons | | LOC_Os08g25200 |  | singletons | | LOC_Os06g08450 |  | singletons | | LOC_Os03g53100 |  | singletons | | LOC_Os06g44410 |  | singletons | | LOC_Os04g28040 |  | singletons | | LOC_Os04g13480 |  | singletons | | LOC_Os01g72330 |  | singletons | | LOC_Os02g58350 |  | singletons | | LOC_Os07g26720 |  | singletons | | LOC_Os07g37140 |  | singletons | | LOC_Os08g08120 |  | singletons | | LOC_Os02g01990 |  | singletons | | LOC_Os08g06630 |  | singletons | | LOC_Os02g28680 |  | singletons | | LOC_Os10g30880 |  | singletons | | LOC_Os02g06370 |  | singletons | | LOC_Os06g05350 |  | singletons | | LOC_Os10g42410 |  | singletons | | LOC_Os11g24130 |  | singletons | | LOC_Os08g44620 |  | singletons | | LOC_Os02g44260 |  | singletons | | LOC_Os09g02790 |  | singletons | | LOC_Os03g12440 |  | singletons | | LOC_Os02g09070 |  | singletons | | LOC_Os06g45540 |  | singletons | | LOC_Os02g07650 |  | singletons | | LOC_Os12g12970 |  | singletons | | LOC_Os01g14420 |  | singletons | | LOC_Os04g47640 |  | singletons | | LOC_Os01g37100 |  | singletons | | LOC_Os03g03900 |  | singletons | | LOC_Os09g37710 |  | singletons | | LOC_Os01g13540 |  | singletons | | LOC_Os04g41850 |  | singletons | | LOC_Os11g16290 |  | singletons | | LOC_Os07g32170 |  | singletons | | LOC_Os04g56170 |  | singletons | | LOC_Os11g30370 |  | singletons | | LOC_Os04g46580 |  | singletons | | LOC_Os05g33810 |  | singletons | | LOC_Os08g40260 |  | singletons | | LOC_Os03g61760 |  | singletons | | LOC_Os01g18850 |  | singletons | | LOC_Os08g14450 |  | singletons | | LOC_Os11g26160 |  | singletons | | LOC_Os03g16430 |  | singletons | | LOC_Os05g51150 |  | singletons | | LOC_Os01g14370 |  | singletons | | LOC_Os01g66890 |  | singletons | | LOC_Os03g49880 |  | singletons | | LOC_Os01g69980 |  | singletons | | LOC_Os04g11830 |  | singletons | | LOC_Os12g42190 |  | singletons | | LOC_Os02g51310 |  | singletons | | LOC_Os07g04510 |  | singletons | | LOC_Os10g25250 |  | singletons | | LOC_Os07g05830 |  | singletons | | LOC_Os09g26780 |  | singletons | | LOC_Os03g27900 |  | singletons | | LOC_Os02g49970 |  | singletons | | LOC_Os09g23650 |  | singletons | | LOC_Os04g32480 |  | singletons | | LOC_Os04g55920 |  | singletons | | LOC_Os04g40930 |  | singletons | | LOC_Os01g70230 |  | singletons | | LOC_Os02g01380 |  | singletons | | LOC_Os02g33610 |  | singletons | | LOC_Os09g38570 |  | singletons | | LOC_Os08g37810 |  | singletons | | LOC_Os03g18340 |  | singletons | | LOC_Os01g52090 |  | singletons | | LOC_Os11g06420 |  | singletons | | LOC_Os02g47640 |  | singletons | | LOC_Os02g08310 |  | singletons | | LOC_Os08g17400 |  | singletons | | LOC_Os03g63810 |  | singletons | | LOC_Os12g40570 |  | singletons | | LOC_Os02g26430 |  | singletons | | LOC_Os04g21950 |  | singletons | | LOC_Os01g62510 |  | singletons | | LOC_Os07g39480 |  | singletons | | LOC_Os05g27730 |  | singletons | | LOC_Os04g39570 |  | singletons | | LOC_Os07g40570 |  | singletons | | LOC_Os03g33012 |  | singletons | | LOC_Os12g32250 |  | singletons | | LOC_Os03g55164 |  | singletons | | LOC_Os05g50610 |  | singletons | | LOC_Os01g43650 |  | singletons | | LOC_Os11g29870 |  | singletons | | LOC_Os01g08710 |  | singletons | | LOC_Os09g25060 |  | singletons | | LOC_Os09g25070 |  | singletons | | LOC_Os05g49210 |  | singletons | | LOC_Os01g14440 |  | singletons | | LOC_Os06g05380 |  | singletons | | LOC_Os01g09080 |  | singletons | | LOC_Os01g40430 |  | singletons | | LOC_Os02g53100 |  | singletons | | LOC_Os01g74140 |  | singletons | | LOC_Os01g62514 |  | singletons | | LOC_Os04g04300 |  | singletons | | LOC_Os11g45850 |  | singletons | | LOC_Os05g14370 |  | singletons | | LOC_Os09g09630 |  | singletons | | LOC_Os03g20550 |  | singletons | | LOC_Os01g60490 |  | singletons | | LOC_Os07g27670 |  | singletons | | LOC_Os06g06360 |  | singletons | | LOC_Os05g25770 |  | singletons | | LOC_Os10g18099 |  | singletons | | LOC_Os08g09900 |  | singletons | | LOC_Os05g03900 |  | singletons | | LOC_Os02g47770 |  | singletons | | LOC_Os05g50310 |  | singletons | | LOC_Os11g13930 |  | singletons | | LOC_Os03g50920 |  | singletons | | LOC_Os06g23030 |  | singletons | | LOC_Os02g48370 |  | singletons | | LOC_Os06g41730 |  | singletons | | LOC_Os09g37250 |  | singletons | | LOC_Os02g27060 |  | singletons | | LOC_Os09g35870 |  | singletons | | LOC_Os06g39590 |  | singletons | | LOC_Os06g22870 |  | singletons | | LOC_Os02g57250 |  | singletons | | LOC_Os02g13520 |  | singletons | | LOC_Os11g11410 |  | singletons | | LOC_Os08g01780 |  | singletons | | LOC_Os02g49160 |  | singletons | | LOC_Os01g18360 |  | singletons | | LOC_Os01g50960 |  | singletons | | LOC_Os02g34840 |  | singletons | | LOC_Os07g42750 |  | singletons | | LOC_Os04g35880 |  | singletons | | LOC_Os07g46690 |  | singletons | | LOC_Os11g05130 |  | singletons | | LOC_Os05g40260 |  | singletons | | LOC_Os08g01170 |  | singletons | | LOC_Os04g40840 |  | singletons | | LOC_Os05g44020 |  | singletons | | LOC_Os03g05710 |  | singletons | | LOC_Os10g35680 |  | singletons | | LOC_Os07g16130 |  | singletons | | LOC_Os01g73480 |  | singletons | | LOC_Os02g56219 |  | singletons | | LOC_Os08g16010 |  | singletons | | LOC_Os03g07990 |  | singletons | | LOC_Os04g39140 |  | singletons | | LOC_Os07g39690 |  | singletons | | LOC_Os03g31690 |  | singletons | | LOC_Os03g09860 |  | singletons | | LOC_Os10g28040 |  | singletons | | LOC_Os05g31254 |  | singletons | | LOC_Os11g32280 |  | singletons | | LOC_Os03g10810 |  | singletons | | LOC_Os12g37490 |  | singletons | | LOC_Os03g49230 |  | singletons | | LOC_Os04g54330 |  | singletons | | LOC_Os05g32180 |  | singletons | | LOC_Os02g53260 |  | singletons | | LOC_Os02g46700 |  | singletons | | LOC_Os01g42470 |  | singletons | | LOC_Os09g37910 |  | singletons | | LOC_Os02g15810 |  | singletons | | LOC_Os01g47600 |  | singletons | | LOC_Os08g01100 |  | singletons | | LOC_Os01g05420 |  | singletons | | LOC_Os11g36450 |  | singletons | | LOC_Os01g36630 |  | singletons | | LOC_Os03g05680 |  | singletons | | LOC_Os05g23670 |  | singletons | | LOC_Os05g10770 |  | singletons | | LOC_Os03g27250 |  | singletons | | LOC_Os03g31594 |  | singletons | | LOC_Os09g22540 |  | singletons | | LOC_Os02g01940 |  | singletons | | LOC_Os03g22540 |  | singletons | | LOC_Os02g58210 |  | singletons | | LOC_Os01g42260 |  | singletons | | LOC_Os01g42270 |  | singletons | | LOC_Os01g08190 |  | singletons | | LOC_Os03g64300 |  | singletons | | LOC_Os04g43130 |  | singletons | | LOC_Os02g56880 |  | singletons | | LOC_Os06g39240 |  | singletons | | LOC_Os08g27850 |  | singletons | | LOC_Os06g11370 |  | singletons | | LOC_Os04g56640 |  | singletons | | LOC_Os03g15990 |  | singletons | | LOC_Os12g34330 |  | singletons | | LOC_Os11g12650 |  | singletons | | LOC_Os09g27620 |  | singletons | | LOC_Os05g03430 |  | singletons | | LOC_Os03g50780 |  | singletons | | LOC_Os01g65600 |  | singletons | | LOC_Os08g01420 |  | singletons | | LOC_Os01g66070 |  | singletons | | LOC_Os12g24540 |  | singletons | | LOC_Os02g03030 |  | singletons | | LOC_Os01g73460 |  | singletons | | LOC_Os06g01320 |  | singletons | | LOC_Os01g08820 |  | singletons | | LOC_Os03g53700 |  | singletons | | LOC_Os03g04980 |  | singletons | | LOC_Os07g31450 |  | singletons | | LOC_Os09g04890 |  | singletons | | LOC_Os06g17280 |  | singletons | | LOC_Os09g38440 |  | singletons | | LOC_Os06g51490 |  | singletons | | LOC_Os06g08790 |  | singletons | | LOC_Os07g49290 |  | singletons | | LOC_Os06g51450 |  | singletons | | LOC_Os06g01170 |  | singletons | | LOC_Os04g59510 |  | singletons | | LOC_Os04g35430 |  | singletons | | LOC_Os03g53630 |  | singletons | | LOC_Os07g07690 |  | singletons | | LOC_Os04g34720 |  | singletons | | LOC_Os02g40510 |  | singletons | | LOC_Os09g36220 |  | singletons | | LOC_Os08g42600 |  | singletons | | LOC_Os11g32900 |  | singletons | | LOC_Os04g52340 |  | singletons | | LOC_Os03g57130 |  | singletons | | LOC_Os02g19880 |  | singletons | | LOC_Os02g19420 |  | singletons | | LOC_Os01g65730 |  | singletons | | LOC_Os07g28840 |  | singletons | | LOC_Os08g14660 |  | singletons | | LOC_Os09g24530 |  | singletons | | LOC_Os05g50980 |  | singletons | | LOC_Os12g13460 |  | singletons | | LOC_Os02g50100 |  | singletons | | LOC_Os02g49326 |  | singletons | | LOC_Os08g08210 |  | singletons | | LOC_Os06g03676 |  | singletons | | LOC_Os04g45990 |  | singletons | | LOC_Os01g70220 |  | singletons | | LOC_Os08g45130 |  | singletons | | LOC_Os07g25450 |  | singletons | | LOC_Os11g38900 |  | singletons | | LOC_Os06g16390 |  | singletons | | LOC_Os03g19480 |  | singletons | | LOC_Os12g41900 |  | singletons | | LOC_Os08g34370 |  | singletons | | LOC_Os09g13740 |  | singletons | | LOC_Os01g46700 |  | singletons | | LOC_Os04g34976 |  | singletons | | LOC_Os02g34850 |  | singletons | | LOC_Os02g47900 |  | singletons | | LOC_Os02g40770 |  | singletons | | LOC_Os01g72310 |  | singletons | | LOC_Os10g41620 |  | singletons | | LOC_Os10g31970 |  | singletons | | LOC_Os02g02050 |  | singletons | | LOC_Os04g47830 |  | singletons | | LOC_Os07g46590 |  | singletons | | LOC_Os07g44210 |  | singletons | | LOC_Os02g06592 |  | singletons | | LOC_Os07g49210 |  | singletons | | LOC_Os01g65850 |  | singletons | | LOC_Os09g27060 |  | singletons | | LOC_Os02g02290 |  | singletons | | LOC_Os03g22900 |  | singletons | | LOC_Os03g01200 |  | singletons | | LOC_Os05g05230 |  | singletons | | LOC_Os01g01312 |  | singletons | | LOC_Os05g15890 |  | singletons | | LOC_Os02g52510 |  | singletons | | LOC_Os06g08480 |  | singletons | | LOC_Os01g44990 |  | singletons | | LOC_Os05g32610 |  | singletons | | LOC_Os02g43460 |  | singletons | | LOC_Os08g14610 |  | singletons | | LOC_Os06g14440 |  | singletons | | LOC_Os07g44800 |  | singletons | | LOC_Os04g09800 |  | singletons | | LOC_Os02g32570 |  | singletons | | LOC_Os01g57110 |  | singletons | | LOC_Os07g40730 |  | singletons | | LOC_Os07g07020 |  | singletons | | LOC_Os10g41450 |  | singletons | | LOC_Os04g31320 |  | singletons | | LOC_Os03g55570 |  | singletons | | LOC_Os08g39310 |  | singletons | | LOC_Os04g56980 |  | singletons | | LOC_Os02g03730 |  | singletons | | LOC_Os09g04720 |  | singletons | | LOC_Os12g32280 |  | singletons | | LOC_Os05g34780 |  | singletons | | LOC_Os08g04780 |  | singletons | | LOC_Os10g38850 |  | singletons | | LOC_Os04g47270 |  | singletons | | LOC_Os02g51880 |  | singletons | | LOC_Os06g46240 |  | singletons | | LOC_Os06g21330 |  | singletons | | LOC_Os05g33050 |  | singletons | | LOC_Os09g16870 |  | singletons | | LOC_Os09g16850 |  | singletons | | LOC_Os09g06890 |  | singletons | | LOC_Os03g48120 |  | singletons | | LOC_Os08g41220 |  | singletons | | LOC_Os04g53430 |  | singletons | | LOC_Os04g53390 |  | singletons | | LOC_Os05g44540 |  | singletons | | LOC_Os10g29440 |  | singletons | | LOC_Os05g44530 |  | singletons | | LOC_Os11g24550 |  | singletons | | LOC_Os11g45560 |  | singletons | | LOC_Os06g45770 |  | singletons | | LOC_Os11g41290 |  | singletons | | LOC_Os11g41300 |  | singletons | | LOC_Os11g41260 |  | singletons | | LOC_Os11g40490 |  | singletons | | LOC_Os06g45720 |  | singletons | | LOC_Os06g14060 |  | singletons | | LOC_Os08g13250 |  | singletons | | LOC_Os08g13090 |  | singletons | | LOC_Os08g03530 |  | singletons | | LOC_Os08g03480 |  | singletons | | LOC_Os08g25240 |  | singletons | | LOC_Os11g40220 |  | singletons | | LOC_Os04g56460 |  | singletons | | LOC_Os07g15490 |  | singletons | | LOC_Os08g38700 |  | singletons | | LOC_Os08g01320 |  | singletons | | LOC_Os05g27880 |  | singletons | | LOC_Os03g46440 |  | singletons | | LOC_Os01g72020 |  | singletons | | LOC_Os01g70670 |  | singletons | | LOC_Os10g07060 |  | No Probe | | LOC_Os12g40120 |  | No Probe | | LOC_Os12g40090 |  | No Probe | | LOC_Os05g40280 |  | No Probe | | LOC_Os03g08620 |  | No Probe | | LOC_Os03g60390 |  | No Probe | | LOC_Os06g09760 |  | No Probe | | LOC_Os01g66270 |  | No Probe | | LOC_Os09g35020 |  | No Probe | | LOC_Os11g13840 |  | No Probe | | LOC_Os04g36640 |  | No Probe | | LOC_Os02g35240 |  | No Probe | | LOC_Os02g42585 |  | No Probe | | LOC_Os05g49700 |  | No Probe | | LOC_Os04g57340 |  | No Probe | | LOC_Os02g09650 |  | No Probe | | LOC_Os02g32040 |  | No Probe | | LOC_Os02g55380 |  | No Probe | | LOC_Os04g56150 |  | No Probe | | LOC_Os02g10760 |  | No Probe | | LOC_Os12g41030 |  | No Probe | | LOC_Os10g30840 |  | No Probe | | LOC_Os02g34270 |  | No Probe | | LOC_Os01g64790 |  | No Probe | | LOC_Os12g03290 |  | No Probe | | LOC_Os02g41800 |  | No Probe | | LOC_Os10g02584 |  | No Probe | | LOC_Os01g56690 |  | No Probe | | LOC_Os07g09590 |  | No Probe | | LOC_Os05g04740 |  | No Probe | | LOC_Os05g06520 |  | No Probe | | LOC_Os09g28900 |  | No Probe | | LOC_Os08g37290 |  | No Probe | | LOC_Os09g24490 |  | No Probe | | LOC_Os11g15210 |  | No Probe | | LOC_Os01g39580 |  | No Probe | | LOC_Os01g39480 |  | No Probe | | LOC_Os09g34330 |  | No Probe | | LOC_Os01g33400 |  | No Probe | | LOC_Os11g38870 |  | No Probe | | LOC_Os02g02480 |  | No Probe | | LOC_Os12g05680 |  | No Probe | | LOC_Os04g41820 |  | No Probe | | LOC_Os03g20650 |  | No Probe | | LOC_Os09g36910 |  | No Probe | | LOC_Os06g50480 |  | No Probe | | LOC_Os06g50600 |  | No Probe | | LOC_Os08g43600 |  | No Probe | | LOC_Os03g19375 |  | No Probe | | LOC_Os12g37415 |  | No Probe | | LOC_Os05g03865 |  | No Probe | | LOC_Os09g13575 |  | No Probe | | LOC_Os06g42690 |  | No Probe | | LOC_Os12g43790 |  | No Probe | | LOC_Os03g47200 |  | No Probe | | LOC_Os02g49880 |  | No Probe | | LOC_Os12g39990 |  | No Probe | | LOC_Os06g17410 |  | No Probe | | LOC_Os01g09720 |  | No Probe | | LOC_Os05g49280 |  | No Probe | | LOC_Os01g24070 |  | No Probe | | LOC_Os07g38410 |  | No Probe | | LOC_Os09g03500 |  | No Probe | | LOC_Os08g20580 |  | No Probe | | LOC_Os08g17640 |  | No Probe | | LOC_Os02g08510 |  | No Probe | | LOC_Os09g38610 |  | No Probe | | LOC_Os06g10470 |  | No Probe | | LOC_Os09g10980 |  | No Probe | | LOC_Os01g39110 |  | No Probe | | LOC_Os04g48375 |  | No Probe | | LOC_Os04g58680 |  | No Probe | | LOC_Os08g33100 |  | No Probe | | LOC_Os04g33950 |  | No Probe | | LOC_Os04g02140 |  | No Probe | | LOC_Os09g08460 |  | No Probe | | LOC_Os11g12490 |  | No Probe | | LOC_Os12g24400 |  | No Probe | | LOC_Os09g25510 |  | No Probe | | LOC_Os07g01270 |  | No Probe | | LOC_Os04g44630 |  | No Probe | | LOC_Os08g33270 |  | No Probe | | LOC_Os04g49380 |  | No Probe | | LOC_Os04g30870 |  | No Probe | | LOC_Os05g03090 |  | No Probe | | LOC_Os07g18840 |  | No Probe | | LOC_Os06g13900 |  | No Probe | | LOC_Os09g01780 |  | No Probe | | LOC_Os11g15755 |  | No Probe | | LOC_Os11g11220 |  | No Probe | | LOC_Os10g19270 |  | No Probe | | LOC_Os12g32140 |  | No Probe | | LOC_Os04g22990 |  | No Probe | | LOC_Os04g10860 |  | No Probe | | LOC_Os02g29640 |  | No Probe | | LOC_Os08g25260 |  | No Probe | | LOC_Os09g11870 |  | No Probe | | LOC_Os09g23230 |  | No Probe | | LOC_Os04g25100 |  | No Probe | | LOC_Os12g18910 |  | No Probe | | LOC_Os06g28150 |  | No Probe | | LOC_Os06g32530 |  | No Probe | | LOC_Os05g30720 |  | No Probe | | LOC_Os08g16370 |  | No Probe | | LOC_Os04g04400 |  | No Probe | | LOC_Os06g42520 |  | No Probe | | LOC_Os05g39480 |  | No Probe | | LOC_Os03g12490 |  | No Probe | | LOC_Os06g42420 |  | No Probe | | LOC_Os11g17430 |  | No Probe | | LOC_Os07g32110 |  | No Probe | | LOC_Os05g37540 |  | No Probe | | LOC_Os04g54880 |  | No Probe | | LOC_Os01g63030 |  | No Probe | | LOC_Os04g28580 |  | No Probe | | LOC_Os02g40950 |  | No Probe | | LOC_Os05g46860 |  | No Probe | | LOC_Os09g32750 |  | No Probe | | LOC_Os02g04640 |  | No Probe | | LOC_Os03g25430 |  | No Probe | | LOC_Os03g40080 |  | No Probe | | LOC_Os11g47890 |  | No Probe | | LOC_Os11g47920 |  | No Probe | | LOC_Os11g47910 |  | No Probe | | LOC_Os12g04200 |  | No Probe | | LOC_Os11g04400 |  | No Probe | | LOC_Os01g67670 |  | No Probe | | LOC_Os12g04380 |  | No Probe | | LOC_Os12g04370 |  | No Probe | | LOC_Os05g40710 |  | No Probe | | LOC_Os12g02870 |  | No Probe | | LOC_Os08g32085 |  | No Probe | | LOC_Os10g39720 |  | No Probe | | LOC_Os08g36220 |  | No Probe | | LOC_Os03g56140 |  | No Probe | | LOC_Os05g25600 |  | No Probe | | LOC_Os05g02730 |  | No Probe | | LOC_Os12g01120 |  | No Probe | | LOC_Os05g48990 |  | No Probe | | LOC_Os07g34880 |  | No Probe | | LOC_Os04g53540 |  | No Probe | | LOC_Os06g36680 |  | No Probe | | LOC_Os03g47042 |  | No Probe | | LOC_Os03g47022 |  | No Probe | | LOC_Os01g48170 |  | No Probe | | LOC_Os01g39220 |  | No Probe | | LOC_Os12g01550 |  | No Probe | | LOC_Os03g57670 |  | No Probe | | LOC_Os01g66030 |  | No Probe | | LOC_Os02g01365 |  | No Probe | | LOC_Os02g01355 |  | No Probe | | LOC_Os04g25870 |  | No Probe | | LOC_Os11g12360 |  | No Probe | | LOC_Os09g02830 |  | No Probe | | LOC_Os01g74440 |  | No Probe | | LOC_Os08g40430 |  | No Probe | | LOC_Os02g36780 |  | No Probe | | LOC_Os09g24800 |  | No Probe | | LOC_Os11g07890 |  | No Probe | | LOC_Os04g46384 |  | No Probe | | LOC_Os08g33800 |  | No Probe | | LOC_Os01g12700 |  | No Probe | | LOC_Os06g14710 |  | No Probe | | LOC_Os06g14010 |  | No Probe | | LOC_Os03g25304 |  | No Probe | | LOC_Os03g63890 |  | No Probe | | LOC_Os05g50350 |  | No Probe | | LOC_Os01g44370 |  | No Probe | | LOC_Os01g47370 |  | No Probe | | LOC_Os03g04070 |  | No Probe | | LOC_Os08g02160 |  | No Probe | | LOC_Os08g44820 |  | No Probe | | LOC_Os12g03040 |  | No Probe | | LOC_Os10g25640 |  | No Probe | | LOC_Os10g26240 |  | No Probe | | LOC_Os07g27340 |  | No Probe | | LOC_Os01g48446 |  | No Probe | | LOC_Os03g62470 |  | No Probe | | LOC_Os03g61319 |  | No Probe | | LOC_Os01g54570 |  | No Probe | | LOC_Os05g39950 |  | No Probe | | LOC_Os05g12808 |  | No Probe | | LOC_Os12g06160 |  | No Probe | | LOC_Os12g01080 |  | No Probe | | LOC_Os04g36070 |  | No Probe | | LOC_Os08g26990 |  | No Probe | | LOC_Os08g28950 |  | No Probe | | LOC_Os08g28900 |  | No Probe | | LOC_Os12g04500 |  | No Probe | | LOC_Os02g42060 |  | No Probe | | LOC_Os09g32944 |  | No Probe | | LOC_Os06g49830 |  | No Probe | | LOC_Os08g43410 |  | No Probe | | LOC_Os05g32070 |  | No Probe | | LOC_Os11g07460 |  | No Probe | | LOC_Os12g02090 |  | No Probe | | LOC_Os05g03740 |  | No Probe | | LOC_Os03g18330 |  | No Probe | | LOC_Os06g30860 |  | No Probe | | LOC_Os05g49100 |  | No Probe | | LOC_Os01g18584 |  | No Probe | | LOC_Os12g02450 |  | No Probe | | LOC_Os12g02440 |  | No Probe | | LOC_Os12g02470 |  | No Probe | | LOC_Os12g02400 |  | No Probe | | LOC_Os12g02420 |  | No Probe | | LOC_Os11g45920 |  | No Probe | | LOC_Os03g45450 |  | No Probe | | LOC_Os08g09810 |  | No Probe | | LOC_Os08g09800 |  | No Probe | | LOC_Os08g09840 |  | No Probe | | LOC_Os12g03110 |  | No Probe | | LOC_Os01g44430 |  | No Probe | | LOC_Os12g10630 |  | No Probe | | LOC_Os04g35500 |  | No Probe | | LOC_Os06g24850 |  | No Probe | | LOC_Os02g34830 |  | No Probe | | LOC_Os03g58030 |  | No Probe | | LOC_Os03g58020 |  | No Probe | | LOC_Os12g07200 |  | No Probe | | LOC_Os07g37790 |  | No Probe | | LOC_Os02g39800 |  | No Probe | | LOC_Os03g51230 |  | No Probe | | LOC_Os04g59624 |  | No Probe | | LOC_Os07g25390 |  | No Probe | | LOC_Os03g55310 |  | No Probe | | LOC_Os08g41120 |  | No Probe | | LOC_Os08g41180 |  | No Probe | | LOC_Os10g29320 |  | No Probe | | LOC_Os10g29190 |  | No Probe | | LOC_Os10g29360 |  | No Probe | | LOC_Os10g28975 |  | No Probe | | LOC_Os02g52316 |  | No Probe | | LOC_Os10g30040 |  | No Probe | | LOC_Os10g29920 |  | No Probe | | LOC_Os10g30350 |  | No Probe | | LOC_Os11g02070 |  | No Probe | | LOC_Os03g42370 |  | No Probe | | LOC_Os02g25820 |  | No Probe | | LOC_Os05g07040 |  | No Probe | | LOC_Os07g12910 |  | No Probe | | LOC_Os05g34730 |  | No Probe | | LOC_Os02g45420 |  | No Probe | | LOC_Os08g43200 |  | No Probe | | LOC_Os04g44670 |  | No Probe | | LOC_Os04g32790 |  | No Probe | | LOC_Os06g08340 |  | No Probe | | LOC_Os06g40150 |  | No Probe | | LOC_Os03g05590 |  | No Probe | | LOC_Os05g36100 |  | No Probe | | LOC_Os11g03540 |  | No Probe | | LOC_Os06g44750 |  | No Probe | | LOC_Os10g33940 |  | No Probe | | LOC_Os04g43910 |  | No Probe | | LOC_Os04g57610 |  | No Probe | | LOC_Os04g28130 |  | No Probe | | LOC_Os10g02509 |  | No Probe | | LOC_Os10g02620 |  | No Probe | | LOC_Os03g58330 |  | No Probe | | LOC_Os03g58830 |  | No Probe | | LOC_Os01g57580 |  | No Probe | | LOC_Os08g33590 |  | No Probe | | LOC_Os08g43070 |  | No Probe | | LOC_Os01g09930 |  | No Probe | | LOC_Os01g09990 |  | No Probe | | LOC_Os11g05480 |  | No Probe | | LOC_Os01g59350 |  | No Probe | | LOC_Os07g48820 |  | No Probe | | LOC_Os04g10260 |  | No Probe | | LOC_Os07g48660 |  | No Probe | | LOC_Os01g59760 |  | No Probe | | LOC_Os01g36220 |  | No Probe | | LOC_Os09g13570 |  | No Probe | | LOC_Os02g09830 |  | No Probe | | LOC_Os06g15330 |  | No Probe | | LOC_Os03g42200 |  | No Probe | | LOC_Os02g43150 |  | No Probe | | LOC_Os05g50270 |  | No Probe | | LOC_Os01g47360 |  | No Probe | | LOC_Os05g06340 |  | No Probe | | LOC_Os12g39400 |  | No Probe | | LOC_Os05g38620 |  | No Probe | | LOC_Os05g02390 |  | No Probe | | LOC_Os11g47620 |  | No Probe | | LOC_Os08g44190 |  | No Probe | | LOC_Os04g01480 |  | No Probe | | LOC_Os02g45480 |  | No Probe | | LOC_Os05g48960 |  | No Probe | | LOC_Os06g32720 |  | No Probe | | LOC_Os02g33430 |  | No Probe | | LOC_Os07g17160 |  | No Probe | | LOC_Os10g06860 |  | No Probe | | LOC_Os08g41710 |  | No Probe | | LOC_Os06g49040 |  | No Probe | | LOC_Os02g47190 |  | No Probe | | LOC_Os06g45410 |  | No Probe | | LOC_Os07g44200 |  | No Probe | | LOC_Os11g47900 |  | No Probe | | LOC_Os11g47870 |  | No Probe | | LOC_Os12g38490 |  | No Probe | | LOC_Os01g67650 |  | No Probe | | LOC_Os05g31380 |  | No Probe | | LOC_Os05g31420 |  | No Probe | | LOC_Os11g04590 |  | No Probe | | LOC_Os11g04570 |  | No Probe | | LOC_Os11g03110 |  | No Probe | | LOC_Os01g45570 |  | No Probe | | LOC_Os09g27450 |  | No Probe | | LOC_Os03g56110 |  | No Probe | | LOC_Os07g03770 |  | No Probe | | LOC_Os03g47036 |  | No Probe | | LOC_Os03g47016 |  | No Probe | | LOC_Os11g01130 |  | No Probe | | LOC_Os01g47710 |  | No Probe | | LOC_Os01g57890 |  | No Probe | | LOC_Os08g08820 |  | No Probe | | LOC_Os11g06020 |  | No Probe | | LOC_Os02g13310 |  | No Probe | | LOC_Os01g39180 |  | No Probe | | LOC_Os11g01550 |  | No Probe | | LOC_Os04g31804 |  | No Probe | | LOC_Os05g34940 |  | No Probe | | LOC_Os04g38770 |  | No Probe | | LOC_Os02g36924 |  | No Probe | | LOC_Os08g33940 |  | No Probe | | LOC_Os09g36250 |  | No Probe | | LOC_Os10g30690 |  | No Probe | | LOC_Os06g07650 |  | No Probe | | LOC_Os05g37050 |  | No Probe | | LOC_Os05g50340 |  | No Probe | | LOC_Os01g44390 |  | No Probe | | LOC_Os02g38130 |  | No Probe | | LOC_Os12g03050 |  | No Probe | | LOC_Os02g42970 |  | No Probe | | LOC_Os02g57650 |  | No Probe | | LOC_Os11g31360 |  | No Probe | | LOC_Os10g25620 |  | No Probe | | LOC_Os07g27330 |  | No Probe | | LOC_Os05g25960 |  | No Probe | | LOC_Os03g61249 |  | No Probe | | LOC_Os03g21870 |  | No Probe | | LOC_Os05g44090 |  | No Probe | | LOC_Os11g05780 |  | No Probe | | LOC_Os11g01074 |  | No Probe | | LOC_Os02g35180 |  | No Probe | | LOC_Os11g04720 |  | No Probe | | LOC_Os04g57720 |  | No Probe | | LOC_Os04g44280 |  | No Probe | | LOC_Os04g28160 |  | No Probe | | LOC_Os09g27190 |  | No Probe | | LOC_Os08g41940 |  | No Probe | | LOC_Os09g36160 |  | No Probe | | LOC_Os01g72490 |  | No Probe | | LOC_Os01g11550 |  | No Probe | | LOC_Os12g07480 |  | No Probe | | LOC_Os02g51280 |  | No Probe | | LOC_Os04g51320 |  | No Probe | | LOC_Os10g41460 |  | No Probe | | LOC_Os02g16540 |  | No Probe | | LOC_Os01g47560 |  | No Probe | | LOC_Os05g04640 |  | No Probe | | LOC_Os11g02530 |  | No Probe | | LOC_Os11g02520 |  | No Probe | | LOC_Os11g02540 |  | No Probe | | LOC_Os12g01180 |  | No Probe | | LOC_Os11g03420 |  | No Probe | | LOC_Os09g26390 |  | No Probe | | LOC_Os02g35100 |  | No Probe | | LOC_Os03g58010 |  | No Probe | | LOC_Os04g52020 |  | No Probe | | LOC_Os02g48810 |  | No Probe | | LOC_Os11g18770 |  | No Probe | | LOC_Os03g19190 |  | No Probe | | LOC_Os08g30910 |  | No Probe | | LOC_Os11g03700 |  | No Probe | | LOC_Os01g59620 |  | No Probe | | LOC_Os04g59620 |  | No Probe | | LOC_Os01g66140 |  | No Probe | | LOC_Os06g31100 |  | No Probe | | LOC_Os04g40630 |  | No Probe | | LOC_Os08g41170 |  | No Probe | | LOC_Os04g35370 |  | No Probe | | LOC_Os10g29260 |  | No Probe | | LOC_Os10g29120 |  | No Probe | | LOC_Os10g28840 |  | No Probe | | LOC_Os02g20600 |  | No Probe | | LOC_Os10g29050 |  | No Probe | | LOC_Os12g02030 |  | No Probe | |
|  |
|  |
|  |
|  |
|  |
|  |
|  |
|  |
|  |
|  |
|  |

**Table S3.** Loss-of-function approach based characterized transcription factors and transcription regulators that play role in morphological or physiological function and their closest paralog with Pearson correlation coefficient score.

| **Locus ID_Characterized gene (KO) Locus ID_Paralog gene Pairwise PCC** |
| --- |
| | LOC_Os09g32948 | LOC_Os08g41950 | 0.93 | | --- | --- | --- | | LOC_Os02g45770 | LOC_Os04g49150 | 0.81 | | LOC_Os06g06750 | LOC_Os03g11614 | 0.79 | | LOC_Os12g41860 | LOC_Os03g43930 | 0.75 | | LOC_Os05g11414 | LOC_Os05g11380 | 0.71 | | LOC_Os11g32110 | LOC_Os12g29520 | 0.67 | | LOC_Os02g39710 | LOC_Os04g42020 | 0.63 | | LOC_Os03g11600 | LOC_Os07g06620 | 0.62 | | LOC_Os03g43400 | LOC_Os12g40890 | 0.6 | | LOC_Os07g31450 | LOC_Os06g01320 | 0.6 | | LOC_Os04g47860 | LOC_Os02g45054 | 0.56 | | LOC_Os01g18870 | LOC_Os04g51070 | 0.55 | | LOC_Os01g19694 | LOC_Os05g03884 | 0.55 | | LOC_Os10g42690 | LOC_Os02g46930 | 0.5 | | LOC_Os04g38720 | LOC_Os02g36880 | 0.46 | | LOC_Os07g48560 | LOC_Os08g14400 | 0.42 | | LOC_Os05g49780 | LOC_Os05g38820 | 0.4 | | LOC_Os03g05510 | LOC_Os08g44940 | 0.39 | | LOC_Os07g03250 | LOC_Os03g56050 | 0.32 | | LOC_Os01g59660 | LOC_Os05g41166 | 0.31 | | LOC_Os05g03040 | LOC_Os03g60430 | 0.29 | | LOC_Os09g27620 | LOC_Os03g50780 | 0.27 | | LOC_Os02g45250 | LOC_Os04g48070 | 0.27 | | LOC_Os02g07430 | LOC_Os06g45650 | 0.26 | | LOC_Os09g31310 | LOC_Os02g48650 | 0.25 | | LOC_Os09g23200 | LOC_Os08g33050 | 0.25 | | LOC_Os02g15350 | LOC_Os04g58190 | 0.23 | | LOC_Os04g08740 | LOC_Os02g57530 | 0.18 | | LOC_Os07g39220 | LOC_Os01g08180 | 0.15 | | LOC_Os09g24480 | LOC_Os07g04510 | 0.12 | | LOC_Os07g08420 | LOC_Os03g58250 | 0.12 | | LOC_Os03g02160 | LOC_Os06g43120 | 0.11 | | LOC_Os03g60430 | LOC_Os07g13170 | 0.06 | | LOC_Os05g10770 | LOC_Os05g23670 | 0.06 | | LOC_Os06g10880 | LOC_Os02g52780 | 0.02 | | LOC_Os07g36460 | LOC_Os01g72370 | -0.04 | | LOC_Os06g40780 | LOC_Os02g10360 | -0.05 | | LOC_Os08g01420 | LOC_Os01g66070 | -0.1 | | LOC_Os01g69850 | LOC_Os04g31804 | -0.16 | |
|  |

**Table S4**. Information on transcription factor and transcription regulator genes in six groups with featured anatomical expression patterns.

| **Locus_ID Group Family Annotation** |
| --- |
| | LOC_Os06g15330 | Leaf / Flag leaf / Shoot | C2C2-CO-like | CCT/B-box zinc finger protein, putative, expressed | | --- | --- | --- | --- | | LOC_Os10g41100 | Leaf / Flag leaf / Shoot | Orphans | CCT motif family protein, expressed | | LOC_Os03g03260 | Leaf / Flag leaf / Shoot | HB | homeobox domain containing protein, expressed | | LOC_Os10g40740 | Leaf / Flag leaf / Shoot | bHLH | helix-loop-helix DNA-binding domain containing protein, expressed | | LOC_Os01g44390 | Leaf / Flag leaf / Shoot | MYB-related | MYB family transcription factor, putative, expressed | | LOC_Os03g14669 | Leaf / Flag leaf / Shoot | CCAAT | core histone H2A/H2B/H3/H4, putative, expressed | | LOC_Os06g24070 | Leaf / Flag leaf / Shoot | G2-like | myb-like DNA-binding domain containing protein, expressed | | LOC_Os11g35390 | Leaf / Flag leaf / Shoot | MYB | MYB family transcription factor, putative, expressed | | LOC_Os05g03900 | Leaf / Flag leaf / Shoot | WRKY | WRKY109, expressed | | LOC_Os10g39030 | Leaf / Flag leaf / Shoot | HB | homeobox domain containing protein, expressed | | LOC_Os08g01170 | Leaf / Flag leaf / Shoot | GNAT | acetyltransferase, GNAT family, putative, expressed | | LOC_Os11g03390 | Leaf / Flag leaf / Shoot | FHA | FHA domain containing protein, putative, expressed | | LOC_Os03g29970 | Leaf / Flag leaf / Shoot | CCAAT | histone-like transcription factor and archaeal histone, putative, expressed | | LOC_Os12g03070 | Leaf / Flag leaf / Shoot | FHA | FHA domain containing protein, putative, expressed | | LOC_Os05g34600 | Leaf / Flag leaf / Shoot | NAC | no apical meristem protein, putative, expressed | | LOC_Os01g38610 | Leaf / Flag leaf / Shoot | bHLH | helix-loop-helix DNA-binding domain containing protein, expressed | | LOC_Os01g58760 | Leaf / Flag leaf / Shoot | bZIP | bZIP transcription factor domain containing protein, expressed | | LOC_Os12g40920 | Leaf / Flag leaf / Shoot | bZIP | bZIP transcription factor domain containing protein, expressed | | LOC_Os12g41650 | Leaf / Flag leaf / Shoot | bHLH | helix-loop-helix DNA-binding domain containing protein, expressed | | LOC_Os05g11510 | Leaf / Flag leaf / Shoot | Orphans | B-box zinc finger family protein, putative, expressed | | LOC_Os02g51450 | Leaf / Flag leaf / Shoot | mTERF | mTERF family protein, expressed | | LOC_Os07g47140 | Leaf / Flag leaf / Shoot | C2C2-CO-like | CCT/B-box zinc finger protein, putative, expressed | | LOC_Os04g39140 | Leaf / Flag leaf / Shoot | GNAT | acetyltransferase, GNAT family, putative, expressed | | LOC_Os11g26160 | Leaf / Flag leaf / Shoot | Sigma70-like | RNA polymerase sigma factor, putative, expressed | | LOC_Os05g44020 | Leaf / Flag leaf / Shoot | GNAT | acetyltransferase, GNAT family, putative, expressed | | LOC_Os07g26150 | Leaf / Flag leaf / Shoot | MYB-related | MYB family transcription factor, putative, expressed | | LOC_Os01g43230 | Leaf / Flag leaf / Shoot | MYB-related | expressed protein | | LOC_Os04g31290 | ROOT | bHLH | helix-loop-helix DNA-binding domain containing protein, expressed | | LOC_Os05g14370 | ROOT | WRKY | WRKY82, expressed | | LOC_Os07g39960 | ROOT | C2H2 | ZOS7-07 - C2H2 zinc finger protein, expressed | | LOC_Os01g53260 | ROOT | WRKY | WRKY23, expressed | | LOC_Os02g58350 | ROOT | Orphans | OsRR3 type-A response regulator, expressed | | LOC_Os02g09070 | ROOT | PLATZ | zinc-binding protein, putative, expressed | | LOC_Os04g23910 | ROOT | MADS | OsMADS25 - MADS-box family gene with MIKCc type-box, expressed | | LOC_Os10g18099 | ROOT | WRKY | WRKY18, expressed | | LOC_Os06g02250 | ROOT | MYB | MYB family transcription factor, putative, expressed | | LOC_Os06g44750 | ROOT | AP2-EREBP | AP2 domain containing protein, expressed | | LOC_Os03g22170 | ROOT | AP2-EREBP | AP2 domain containing protein, expressed | | LOC_Os07g04560 | ROOT | NAC | no apical meristem protein, putative, expressed | | LOC_Os03g58420 | ROOT | WRKY | WRKY6, expressed | | LOC_Os12g32400 | ROOT | bHLH | helix-loop-helix DNA-binding domain containing protein, expressed | | LOC_Os02g53100 | ROOT | WRKY | WRKY32, expressed | | LOC_Os01g52410 | ROOT | MYB | myb-like DNA-binding domain containing protein, putative, expressed | | LOC_Os07g39940 | ROOT | bHLH | helix-loop-helix DNA-binding domain containing protein, expressed | | LOC_Os05g40060 | ROOT | WRKY | WRKY48, expressed | | LOC_Os03g05500 | ROOT | LOB | DUF260 domain containing protein, putative, expressed | | LOC_Os01g02110 | ROOT | bHLH | helix-loop-helix DNA-binding domain containing protein, expressed | | LOC_Os07g22770 | ROOT | AP2-EREBP | AP2 domain containing protein, expressed | | LOC_Os05g20930 | ROOT | C2H2 | ZOS5-07 - C2H2 zinc finger protein, expressed | | LOC_Os02g54520 | ROOT | MYB | MYB family transcription factor, putative, expressed | | LOC_Os09g26170 | ROOT | MYB | MYB family transcription factor, putative, expressed | | LOC_Os02g10140 | ROOT | bZIP | bZIP transcription factor domain containing protein, expressed | | LOC_Os02g51799 | ROOT | MYB | MYB family transcription factor, putative, expressed | | LOC_Os02g15340 | ROOT | NAC | no apical meristem protein, putative, expressed | | LOC_Os02g57490 | ROOT | LOB | DUF260 domain containing protein, putative, expressed | | LOC_Os04g50920 | ROOT | WRKY | WRKY37, expressed | | LOC_Os04g52810 | ROOT | NAC | no apical meristem protein, putative, expressed | | LOC_Os06g07040 | ROOT | AUX_IAA | OsIAA20 - Auxin-responsive Aux/IAA gene family member, expressed | | LOC_Os03g20910 | ROOT | HB | homeobox domain containing protein, expressed | | LOC_Os10g25170 | ROOT | AP2-EREBP | AP2 domain containing protein, expressed | | LOC_Os05g40070 | ROOT | WRKY | WRKY84, expressed | | LOC_Os11g02470 | ROOT | WRKY | WRKY52, expressed | | LOC_Os02g40530 | ROOT | MYB | MYB family transcription factor, putative, expressed | | LOC_Os08g44830 | ROOT | C2H2 | ZOS8-14 - C2H2 zinc finger protein, expressed | | LOC_Os04g42950 | ROOT | MYB | MYB family transcription factor, putative, expressed | | LOC_Os03g19370 | ROOT | bZIP | CPuORF4 - conserved peptide uORF-containing transcript, expressed | | LOC_Os11g47630 | ROOT | C2H2 | ZOS11-10 - C2H2 zinc finger protein, expressed | | LOC_Os09g23620 | ROOT | MYB | MYB family transcription factor, putative, expressed | | LOC_Os03g42630 | ROOT | NAC | No apical meristem protein, putative, expressed | | LOC_Os01g07450 | ROOT | MYB | MYB family transcription factor, putative, expressed | | LOC_Os01g53040 | ROOT | WRKY | WRKY14, expressed | | LOC_Os03g43410 | ROOT | AUX_IAA | OsIAA12 - Auxin-responsive Aux/IAA gene family member, expressed | | LOC_Os02g40070 | ROOT | AP2-EREBP | AP2-like ethylene-responsive transcription factor PLETHORA 2, putative, expressed | | LOC_Os09g29820 | ROOT | bZIP | bZIP transcription factor domain containing protein, expressed | | LOC_Os05g02390 | ROOT | C2H2 | ZOS5-02 - C2H2 zinc finger protein, expressed | | LOC_Os04g08290 | ROOT | C2H2 | ZOS4-04 - C2H2 zinc finger protein, expressed | | LOC_Os03g41330 | ROOT | LOB | DUF260 domain containing protein, putative, expressed | | LOC_Os03g56090 | ROOT | MYB | MYB family transcription factor, putative, expressed | | LOC_Os12g07640 | ROOT | MYB | MYB family transcription factor, putative, expressed | | LOC_Os04g43560 | ROOT | NAC | no apical meristem protein, putative, expressed | | LOC_Os01g01430 | ROOT | NAC | No apical meristem protein, putative, expressed | | LOC_Os06g44410 | ROOT | Orphans | histidine kinase, putative, expressed | | LOC_Os08g13840 | ROOT | WRKY | WRKY25, expressed | | LOC_Os01g09080 | ROOT | WRKY | WRKY107, expressed | | LOC_Os12g39330 | ROOT | AP2-EREBP | AP2 domain containing protein, expressed | | LOC_Os07g48870 | ROOT | MYB | MYB family transcription factor, putative, expressed | | LOC_Os09g33580 | SAM/PANICLE | bHLH | BEE 1, putative, expressed | | LOC_Os06g45540 | SAM/PANICLE | PLATZ | zinc-binding protein, putative, expressed | | LOC_Os04g35430 | SAM/PANICLE | PHD | PHD-finger domain containing protein, expressed | | LOC_Os03g51690 | SAM/PANICLE | HB | Homeobox domain containing protein, expressed | | LOC_Os08g42470 | SAM/PANICLE | bHLH | BEE 1, putative, expressed | | LOC_Os04g45330 | SAM/PANICLE | C2C2-YABBY | YABBY domain containing protein, putative, expressed | | LOC_Os03g21160 | SAM/PANICLE | C3H | RNA-binding zinc finger protein, putative, expressed | | LOC_Os04g44440 | SAM/PANICLE | TCP | TCP family transcription factor, putative, expressed | | LOC_Os02g42950 | SAM/PANICLE | C2C2-YABBY | YABBY domain containing protein, putative, expressed | | LOC_Os03g47140 | SAM/PANICLE | GRF | growth regulating factor protein, putative, expressed | | LOC_Os08g41940 | SAM/PANICLE | SBP | OsSPL16 - SBP-box gene family member, expressed | | LOC_Os10g42490 | SAM/PANICLE | HB | homeobox and START domains containing protein, putative, expressed | | LOC_Os02g13310 | SAM/PANICLE | HB | homeobox domain containing protein, expressed | | LOC_Os01g08190 | SAM/PANICLE | LUG | transcriptional corepressor LEUNIG, putative, expressed | | LOC_Os01g45570 | SAM/PANICLE | HB | homeobox associated leucine zipper, putative, expressed | | LOC_Os10g36420 | SAM/PANICLE | C2C2-YABBY | YABBY domain containing protein, putative, expressed | | LOC_Os02g07650 | SAM/PANICLE | PLATZ | zinc-binding protein, putative, expressed | | LOC_Os09g32948 | SAM/PANICLE | MADS | OsMADS8 - MADS-box family gene with MIKCc type-box, expressed | | LOC_Os03g11370 | SAM/PANICLE | ABI3VP1 | B3 DNA binding domain containing protein, expressed | | LOC_Os03g54170 | SAM/PANICLE | MADS | OsMADS34 - MADS-box family gene with MIKCc type-box, expressed | | LOC_Os02g45770 | SAM/PANICLE | MADS | OsMADS6 - MADS-box family gene with MIKCc type-box, expressed | | LOC_Os01g10504 | SAM/PANICLE | MADS | OsMADS3 - MADS-box family gene with MIKCc type-box, expressed | | LOC_Os08g41950 | SAM/PANICLE | MADS | OsMADS7 - MADS-box family gene with MIKCc type-box, expressed | | LOC_Os06g06750 | SAM/PANICLE | MADS | OsMADS5 - MADS-box family gene with MIKCc type-box, expressed | | LOC_Os05g11414 | SAM/PANICLE | MADS | OsMADS58 - MADS-box family gene with MIKCc type-box, expressed | | LOC_Os04g49150 | SAM/PANICLE | MADS | OsMADS17 - MADS-box family gene with MIKCc type-box, expressed | | LOC_Os01g11350 | SAM/PANICLE | bZIP | bZIP transcription factor domain containing protein, expressed | | LOC_Os02g47280 | SAM/PANICLE | GRF | growth-regulating factor, putative, expressed | | LOC_Os04g51000 | SAM/PANICLE | LFY | transcription factor FL, putative, expressed | | LOC_Os04g56850 | SAM/PANICLE | ARF | auxin response factor, putative, expressed | | LOC_Os03g11600 | SAM/PANICLE | C2C2-YABBY | YABBY domain containing protein, putative, expressed | | LOC_Os06g15480 | SAM/PANICLE | bZIP | transcription factor, putative, expressed | | LOC_Os03g12950 | SAM/PANICLE | AP2-EREBP | AP2-like ethylene-responsive transcription factor AINTEGUMENTA, putative, expressed | | LOC_Os08g39890 | SAM/PANICLE | SBP | OsSPL14 - SBP-box gene family member, expressed | | LOC_Os01g52680 | SAM/PANICLE | MADS | OsMADS32 - MADS-box family gene with MIKCc type-box, expressed | | LOC_Os03g06920 | SAM/PANICLE | AP2-EREBP | DRD1, putative, expressed | | LOC_Os02g52510 | SAM/PANICLE | SNF2 | SNF2 family N-terminal domain containing protein, expressed | | LOC_Os01g18870 | SAM/PANICLE | bHLH | helix-loop-helix DNA-binding domain containing protein, expressed | | LOC_Os03g49880 | SAM/PANICLE | TCP | TCP family transcription factor, putative, expressed | | LOC_Os02g03030 | SAM/PANICLE | PHD | ATXR, putative, expressed | | LOC_Os04g50090 | SAM/PANICLE | bHLH | helix-loop-helix DNA-binding protein, putative, expressed | | LOC_Os04g47640 | SAM/PANICLE | RWP-RK | RWP-RK domain-containing protein, putative, expressed | | LOC_Os03g10150 | SAM/PANICLE | OFP | DUF623 domain containing protein, expressed | | LOC_Os03g42420 | SAM/PANICLE | ABI3VP1 | B3 DNA binding domain containing protein, expressed | | LOC_Os04g48510 | SAM/PANICLE | GRF | growth regulating factor protein, putative, expressed | | LOC_Os04g33870 | SAM/PANICLE | OFP | DUF623 domain containing protein, expressed | | LOC_Os03g50780 | SAM/PANICLE | PHD | PHD-finger domain containing protein, putative, expressed | | LOC_Os01g61480 | SAM/PANICLE | bHLH | helix-loop-helix DNA-binding domain containing protein, expressed | | LOC_Os10g32900 | ANTHER / POLLEN | Orphans | CCT motif family protein, expressed | | LOC_Os01g19050 | ANTHER / POLLEN | FAR1 | transposon protein, putative, unclassified, expressed | | LOC_Os10g35930 | ANTHER / POLLEN | LIM | OsPLIM2c - LIM domain protein, putative actin-binding protein and transcription factor, expressed | | LOC_Os02g36510 | ANTHER / POLLEN | EIL | ethylene-insensitive 3, putative, expressed | | LOC_Os06g11970 | ANTHER / POLLEN | MADS | OsMADS63 - MADS-box family gene with MIKC* type-box, expressed | | LOC_Os04g46670 | ANTHER / POLLEN | C2H2 | ZOS4-09 - C2H2 zinc finger protein, expressed | | LOC_Os02g01990 | ANTHER / POLLEN | Orphans | CCT motif family protein, expressed | | LOC_Os03g31240 | ANTHER / POLLEN | C2H2 | ZOS3-10 - C2H2 zinc finger protein, expressed | | LOC_Os02g57790 | ANTHER / POLLEN | C2H2 | ZOS2-19 - C2H2 zinc finger protein, expressed | | LOC_Os07g37920 | ANTHER / POLLEN | NAC | no apical meristem protein, putative, expressed | | LOC_Os03g18340 | ANTHER / POLLEN | Trihelix | transcription factor like protein, putative, expressed | | LOC_Os02g42820 | ANTHER / POLLEN | LIM | OsPLIM2a - LIM domain protein, putative actin-binding protein and transcription factor, expressed | | LOC_Os04g45010 | ANTHER / POLLEN | LIM | OsPLIM2b - LIM domain protein, putative actin-binding protein and transcription factor, expressed | | LOC_Os06g46560 | ANTHER / POLLEN | MYB | myb-like DNA-binding domain containing protein, expressed | | LOC_Os08g31080 | ANTHER / POLLEN | LOB | DUF260 domain containing protein, putative, expressed | | LOC_Os11g43740 | ANTHER / POLLEN | MADS | OsMADS68 - MADS-box family gene with MIKC* type-box, expressed | | LOC_Os01g51610 | ANTHER / POLLEN | ABI3VP1 | B3 DNA binding domain containing protein, expressed | | LOC_Os12g09250 | ANTHER / POLLEN | bZIP | bZIP transcription factor domain containing protein, expressed | | LOC_Os02g44120 | ANTHER / POLLEN | C2H2 | ZOS2-13 - C2H2 zinc finger protein, expressed | | LOC_Os03g27900 | ANTHER / POLLEN | Tify | ZIM motif family protein, expressed | | LOC_Os09g19950 | ANTHER / POLLEN | LOB | DUF260 domain containing protein, putative, expressed | | LOC_Os04g58000 | ANTHER / POLLEN | ABI3VP1 | B3 DNA binding domain containing protein, expressed | | LOC_Os03g41600 | ANTHER / POLLEN | LOB | DUF260 domain containing protein, putative, expressed | | LOC_Os08g38590 | ANTHER / POLLEN | MADS | OsMADS62 - MADS-box family gene with MIKC* type-box, expressed | | LOC_Os04g39570 | ANTHER / POLLEN | WRKY | WRKY35, expressed | | LOC_Os02g44130 | ANTHER / POLLEN | C2H2 | ZOS2-14 - C2H2 zinc finger protein, expressed | | LOC_Os02g40770 | ANTHER / POLLEN | SET | SET domain containing protein, expressed | | LOC_Os04g46680 | ANTHER / POLLEN | C2H2 | ZOS4-10 - C2H2 zinc finger protein, expressed | | LOC_Os03g04620 | ANTHER / POLLEN | Orphans | CCT motif family protein, expressed | | LOC_Os01g01470 | SEED / EMBRYO / ENDOSPERM | NAC | no apical meristem protein, putative, expressed | | LOC_Os04g28120 | SEED / EMBRYO / ENDOSPERM | Orphans | response regulator receiver domain containing protein, expressed | | LOC_Os01g07930 | SEED / EMBRYO / ENDOSPERM | C3H | zinc finger C-x8-C-x5-C-x3-H type family protein, expressed | | LOC_Os11g31360 | SEED / EMBRYO / ENDOSPERM | NAC | no apical meristem protein, putative, expressed | | LOC_Os11g31380 | SEED / EMBRYO / ENDOSPERM | NAC | no apical meristem protein, putative, expressed | | LOC_Os02g15350 | SEED / EMBRYO / ENDOSPERM | C2C2-Dof | dof zinc finger domain containing protein, putative, expressed | | LOC_Os07g15540 | SEED / EMBRYO / ENDOSPERM | Orphans | ethylene receptor, putative, expressed | | LOC_Os03g53100 | SEED / EMBRYO / ENDOSPERM | Orphans | response regulator receiver domain containing protein, expressed | | LOC_Os01g24460 | SEED / EMBRYO / ENDOSPERM | CCAAT | histone-like transcription factor and archaeal histone, putative, expressed | | LOC_Os08g45110 | SEED / EMBRYO / ENDOSPERM | AP2-EREBP | AP2 domain containing protein, expressed | | LOC_Os01g39850 | SEED / EMBRYO / ENDOSPERM | CCAAT | histone-like transcription factor and archaeal histone, putative, expressed | | LOC_Os10g11580 | SEED / EMBRYO / ENDOSPERM | CCAAT | histone-like transcription factor and archaeal histone, putative, expressed | | LOC_Os09g34880 | SEED / EMBRYO / ENDOSPERM | bZIP | basic region leucine zipper domain containing protein, expressed | | LOC_Os10g25850 | SEED / EMBRYO / ENDOSPERM | CCAAT | nuclear transcription factor Y subunit, putative, expressed | | LOC_Os01g29840 | SEED / EMBRYO / ENDOSPERM | NAC | no apical meristem protein, putative, expressed | | LOC_Os01g74590 | SEED / EMBRYO / ENDOSPERM | MYB | MYB family transcription factor, putative, expressed | | LOC_Os02g19420 | SEED / EMBRYO / ENDOSPERM | Rcd1-like | expressed protein | | LOC_Os02g12310 | SEED / EMBRYO / ENDOSPERM | NAC | no apical meristem protein, putative, expressed | | LOC_Os02g07430 | SEED / EMBRYO / ENDOSPERM | MADS | OsMADS29 - MADS-box family gene with MIKCc type-box, expressed | | LOC_Os03g29614 | SEED / EMBRYO / ENDOSPERM | MYB | myb-like DNA-binding domain containing protein, putative, expressed | | LOC_Os01g50110 | SEED / EMBRYO / ENDOSPERM | MYB | MYB family transcription factor, putative, expressed | | LOC_Os01g63680 | SEED / EMBRYO / ENDOSPERM | MYB | MYB family transcription factor, putative, expressed | | LOC_Os07g08420 | SEED / EMBRYO / ENDOSPERM | bZIP | bZIP transcription factor domain containing protein, expressed | | LOC_Os02g09910 | SEED / EMBRYO / ENDOSPERM | PHD | PHD-finger domain containing protein, putative, expressed | | LOC_Os07g31470 | SEED / EMBRYO / ENDOSPERM | MYB | MYB family transcription factor, putative, expressed | | LOC_Os05g34310 | SEED / EMBRYO / ENDOSPERM | NAC | no apical meristem protein, putative, expressed | | LOC_Os05g23910 | SEED / EMBRYO / ENDOSPERM | CCAAT | histone-like transcription factor and archaeal histone, putative, expressed | | LOC_Os01g01290 | SEED / EMBRYO / ENDOSPERM | CCAAT | histone-like transcription factor and archaeal histone, putative, expressed | | LOC_Os01g66290 | SEED / EMBRYO / ENDOSPERM | MADS | OsMADS21 - MADS-box family gene with MIKCc type-box, expressed | | LOC_Os08g41030 | SEED / EMBRYO / ENDOSPERM | AP2-EREBP | AP2 domain containing protein, expressed | | LOC_Os02g28580 | SEED / EMBRYO / ENDOSPERM | Orphans | expressed protein | | LOC_Os01g62310 | SEED / EMBRYO / ENDOSPERM | HB | homeobox domain containing protein, expressed | | LOC_Os05g49930 | SEED / EMBRYO / ENDOSPERM | GRAS | GRAS family transcription factor domain containing protein, expressed | | LOC_Os05g28320 | SEED / EMBRYO / ENDOSPERM | MYB | myb-like DNA-binding domain containing protein, putative, expressed | | LOC_Os11g31340 | SEED / EMBRYO / ENDOSPERM | NAC | no apical meristem protein, putative, expressed | | LOC_Os03g19900 | SEED / EMBRYO / ENDOSPERM | AP2-EREBP | AP2 domain containing protein, expressed | | LOC_Os01g33350 | SEED / EMBRYO / ENDOSPERM | PLATZ | zinc-binding protein, putative, expressed | | LOC_Os01g68370 | SEED / EMBRYO / ENDOSPERM | ABI3VP1 | B3 DNA binding domain containing protein, expressed | | LOC_Os01g64000 | SEED / EMBRYO / ENDOSPERM | bZIP | bZIP transcription factor, putative, expressed | | LOC_Os11g31330 | SEED / EMBRYO / ENDOSPERM | NAC | no apical meristem protein, putative, expressed | | LOC_Os02g49410 | SEED / EMBRYO / ENDOSPERM | CCAAT | histone-like transcription factor and archaeal histone, putative, expressed | | LOC_Os06g17480 | SEED / EMBRYO / ENDOSPERM | CCAAT | histone-like transcription factor and archaeal histone, putative, expressed | | LOC_Os05g36100 | SEED / EMBRYO / ENDOSPERM | AP2-EREBP | AP2 domain containing protein, expressed | | LOC_Os04g51070 | SEED / EMBRYO / ENDOSPERM | bHLH | helix-loop-helix DNA-binding domain containing protein, expressed | | LOC_Os01g53650 | SEED / EMBRYO / ENDOSPERM | C3H | zinc finger CCCH type family protein, putative, expressed | | LOC_Os12g10540 | SEED / EMBRYO / ENDOSPERM | MADS | OsMADS13 - MADS-box family gene with MIKCc type-box, expressed | | LOC_Os05g48960 | UBIQUITOUS | C3H | splicing factor U2AF, putative, expressed | | LOC_Os06g51220 | UBIQUITOUS | HMG | HMG1/2, putative, expressed | | LOC_Os03g53960 | UBIQUITOUS | MYB-related | transcriptional adaptor, putative, expressed | | LOC_Os06g31100 | UBIQUITOUS | TRAF | E1-BTB2 - Bric-a-Brac, Tramtrack, and Broad Complex domain with E1 subfamily conserved sequence, expressed | | LOC_Os01g48444 | UBIQUITOUS | AUX_IAA | OsIAA5 - Auxin-responsive Aux/IAA gene family member, expressed | | LOC_Os07g36140 | UBIQUITOUS | CCAAT | core histone H2A/H2B/H3/H4, putative, expressed | | LOC_Os03g21240 | UBIQUITOUS | G2-like | myb-like DNA-binding domain containing protein, expressed | | LOC_Os02g45480 | UBIQUITOUS | C3H | nucleic acid binding protein, putative, expressed | | LOC_Os01g51154 | UBIQUITOUS | MYB-related | single myb histone, putative, expressed | | LOC_Os08g27850 | UBIQUITOUS | MBF1 | endothelial differentiation-related factor 1, putative, expressed | | LOC_Os02g04490 | UBIQUITOUS | PHD | histone acetyltransferase HAC1, putative, expressed | | LOC_Os04g55920 | UBIQUITOUS | Tify | zinc-finger protein, putative, expressed | | LOC_Os02g49700 | UBIQUITOUS | HB | homeobox associated leucine zipper, putative, expressed | | LOC_Os09g26390 | UBIQUITOUS | ARID | AT-rich interaction region, putative, expressed | | LOC_Os05g14180 | UBIQUITOUS | AUX_IAA | OsIAA17 - Auxin-responsive Aux/IAA gene family member, expressed | | LOC_Os12g13170 | UBIQUITOUS | bZIP | transcription factor, putative, expressed | | LOC_Os01g16670 | UBIQUITOUS | BSD | BSD domain-containing protein, putative, expressed | | LOC_Os07g28430 | UBIQUITOUS | GRF | growth regulating factor protein, putative, expressed | | LOC_Os04g54330 | UBIQUITOUS | GNAT | acetyltransferase, GNAT family, putative, expressed | | LOC_Os07g25710 | UBIQUITOUS | G2-like | myb-like DNA-binding domain containing protein, expressed | | LOC_Os11g08080 | UBIQUITOUS | MYB-related | SWIRM domain containing protein, expressed | | LOC_Os02g57650 | UBIQUITOUS | NAC | No apical meristem protein, putative, expressed | | LOC_Os05g01020 | UBIQUITOUS | Orphans | transcriptional repressor, putative, expressed | | LOC_Os02g39520 | UBIQUITOUS | FAR1 | transposon protein, putative, unclassified, expressed | | LOC_Os08g39310 | UBIQUITOUS | SWI_SNF-BAF60b | SWIB/MDM2 domain containing protein, expressed | | LOC_Os06g09390 | UBIQUITOUS | AP2-EREBP | AP2 domain containing protein, expressed | | LOC_Os06g51490 | UBIQUITOUS | PHD | PHD-finger domain containing protein, putative, expressed | | LOC_Os07g49030 | UBIQUITOUS | PHD | PHD-finger family protein, expressed | | LOC_Os01g48370 | UBIQUITOUS | TUB | OsFBT1 - F-box and tubby domain containing protein, expressed | | LOC_Os05g48820 | UBIQUITOUS | HB | DDT, putative, expressed | | LOC_Os02g08544 | UBIQUITOUS | HB | homeobox protein knotted-1, putative, expressed | | LOC_Os08g29500 | UBIQUITOUS | CCAAT | histone-like transcription factor and archaeal histone, putative, expressed | | LOC_Os05g03550 | UBIQUITOUS | MYB | MYB family transcription factor, putative, expressed | | LOC_Os03g22900 | UBIQUITOUS | SNF2 | SNF2 family N-terminal domain containing protein, expressed | | LOC_Os06g41730 | UBIQUITOUS | ARID | ARID/BRIGHT DNA-binding domain-containing protein, putative, expressed | | LOC_Os09g30310 | UBIQUITOUS | CCAAT | core histone H2A/H2B/H3/H4, putative, expressed | | LOC_Os04g47690 | UBIQUITOUS | HMG | HMG1/2, putative, expressed | | LOC_Os01g46700 | UBIQUITOUS | SET | SET domain containing protein, expressed | | LOC_Os03g53700 | UBIQUITOUS | PHD | PHD-finger domain containing protein, putative, expressed | | LOC_Os04g40420 | UBIQUITOUS | MYB-related | SWIRM domain containing protein, expressed | | LOC_Os03g13790 | UBIQUITOUS | MYB-related | myb/SANT domain protein, putative, expressed | | LOC_Os02g51300 | UBIQUITOUS | AP2-EREBP | AP2 domain containing protein, expressed | | LOC_Os12g37410 | UBIQUITOUS | bZIP | CPuORF5 - conserved peptide uORF-containing transcript, expressed | | LOC_Os01g17260 | UBIQUITOUS | bZIP | transcription factor, putative, expressed | | LOC_Os11g34200 | UBIQUITOUS | CCAAT | histone-like transcription factor and archaeal histone, putative, expressed | | LOC_Os01g14370 | UBIQUITOUS | TAZ | histone acetyltransferase HAC5, putative, expressed | | LOC_Os06g10690 | UBIQUITOUS | PHD | PHD-finger domain containing protein, putative, expressed | | LOC_Os09g26420 | UBIQUITOUS | AP2-EREBP | AP2 domain containing protein, expressed | | LOC_Os03g50110 | UBIQUITOUS | GeBP | transcription regulator, putative, expressed | | LOC_Os09g13740 | UBIQUITOUS | SET | SET domain containing protein, expressed | | LOC_Os06g11370 | UBIQUITOUS | MED6 | mediator of RNA polymerase II transcription subunit 6, putative, expressed | | LOC_Os05g41240 | UBIQUITOUS | G2-like | Myb-like DNA-binding domain containing protein, putative, expressed | | LOC_Os02g02290 | UBIQUITOUS | SNF2 | SNF2 family N-terminal domain containing protein, expressed | | LOC_Os01g04020 | UBIQUITOUS | AP2-EREBP | AP2 domain containing protein, expressed | | LOC_Os07g31450 | UBIQUITOUS | PHD | CHR4/MI-2-LIKE, putative, expressed | | LOC_Os05g33810 | UBIQUITOUS | SBP | OsSPL9 - SBP-box gene family member, expressed | | LOC_Os08g29660 | UBIQUITOUS | WRKY | WRKY69, expressed | | LOC_Os09g36090 | UBIQUITOUS | C3H | plus-3 domain containing protein, expressed | | LOC_Os03g20790 | UBIQUITOUS | EIL | ethylene-insensitive 3, putative, expressed | | LOC_Os11g11100 | UBIQUITOUS | bZIP | bZIP transcription factor domain containing protein, expressed | | LOC_Os07g39480 | UBIQUITOUS | WRKY | WRKY87, expressed | | LOC_Os03g09280 | UBIQUITOUS | GRAS | gibberellin response modulator protein, putative, expressed | | LOC_Os07g35870 | UBIQUITOUS | bHLH | basic helix-loop-helix DND-binding domain containing protein, expressed | | LOC_Os02g54160 | UBIQUITOUS | AP2-EREBP | AP2 domain containing protein, expressed | |
|  |

**Table S5**. Information on monocot and rice-divergent transcription factor and transcription regulators genes with their meta-expression group. Orthologs were identified with InParanoid v4.1 and the OMA browser (Altenhoff et al. 2015; Sonnhammer and Östlund 2015) and these datasets were used to define monocot- and rice-divergent genes.

| **Locus_ID Specificity Featured expression Family** |
| --- |
| | LOC_Os10g41100 | Monocot Divergent | Leaf/Flag leaf/Shoot (Group A), | Orphans | | --- | --- | --- | --- | | LOC_Os03g22170 | Rice divergent | Root (Group B),Root_IAA_3 hrs,Root_JA_1 hr | AP2-EREBP | | LOC_Os10g25170 | Rice divergent | Root (Group B) | AP2-EREBP | | LOC_Os04g08290 | Rice divergent | Root (Group B) | C2H2 | | LOC_Os10g18099 | Rice divergent | Root (Group B) | WRKY | | LOC_Os02g10140 | Monocot Divergent | Root (Group B) | bZIP | | LOC_Os03g19370 | Monocot Divergent | Root (Group B),XOO | bZIP | | LOC_Os07g39960 | Monocot Divergent | Root (Group B),Drought ,Root_ABA_3 hrs,Shoot_ABA_6 and 12 hrs | C2H2 | | LOC_Os11g47630 | Monocot Divergent | Root (Group B),Low temperature,M. grisea | C2H2 | | LOC_Os03g41330 | Monocot Divergent | Root (Group B) | LOB | | LOC_Os09g26170 | Monocot Divergent | Root (Group B) | MYB | | LOC_Os01g07450 | Monocot Divergent | Root (Group B) | MYB | | LOC_Os01g01430 | Monocot Divergent | Root (Group B),Root_JA_6 hrs | NAC | | LOC_Os03g58420 | Monocot Divergent | Root (Group B) | WRKY | | LOC_Os01g18870 | Monocot Divergent | Sam/Panicle (Group C) | bHLH | | LOC_Os01g61480 | Monocot Divergent | Sam/Panicle (Group C) | bHLH | | LOC_Os03g47140 | Monocot Divergent | Sam/Panicle (Group C) | GRF | | LOC_Os01g52680 | Monocot Divergent | Sam/Panicle (Group C) | MADS | | LOC_Os02g01990 | Rice divergent | Anther/Pollen (Group D) | Orphans | | LOC_Os03g27900 | Rice divergent | Anther/Pollen (Group D) | Tify | | LOC_Os01g19050 | Monocot Divergent | Anther/Pollen (Group D) | FAR1 | | LOC_Os08g38590 | Monocot Divergent | Anther/Pollen (Group D) | MADS | | LOC_Os06g46560 | Monocot Divergent | Anther/Pollen (Group D) | MYB | | LOC_Os03g18340 | Monocot Divergent | Anther/Pollen (Group D) | Trihelix | | LOC_Os04g39570 | Monocot Divergent | Anther/Pollen (Group D) | WRKY | | LOC_Os09g34880 | Rice divergent | Seed/Embryo/Endopserm (Group E) | bZIP | | LOC_Os01g53650 | Rice divergent | Seed/Embryo/Endopserm (Group E),Drought ,Root_ABA_3 hrs | C3H | | LOC_Os10g25850 | Rice divergent | Seed/Embryo/Endopserm (Group E) | CCAAT | | LOC_Os05g34310 | Rice divergent | Seed/Embryo/Endopserm (Group E) | NAC | | LOC_Os02g12310 | Rice divergent | Seed/Embryo/Endopserm (Group E),BPH | NAC | | LOC_Os04g28120 | Rice divergent | Seed/Embryo/Endopserm (Group E),Submergance | Orphans | | LOC_Os03g19900 | Monocot Divergent | Seed/Embryo/Endopserm (Group E),Submergance,Root_ABA_3 hrs,Root_JA_1 hr,Root_JA_3 hrs,Root_JA_6 hrs | AP2-EREBP | | LOC_Os08g41030 | Monocot Divergent | Seed/Embryo/Endopserm (Group E),XOO,Root_ABA_3 hrs | AP2-EREBP | | LOC_Os02g15350 | Monocot Divergent | Seed/Embryo/Endopserm (Group E),Submergance | C2C2-Dof | | LOC_Os01g07930 | Monocot Divergent | Seed/Embryo/Endopserm (Group E) | C3H | | LOC_Os02g49410 | Monocot Divergent | Seed/Embryo/Endopserm (Group E) | CCAAT | | LOC_Os06g17480 | Monocot Divergent | Seed/Embryo/Endopserm (Group E),Root_ABA_3 hrs | CCAAT | | LOC_Os10g11580 | Monocot Divergent | Seed/Embryo/Endopserm (Group E) | CCAAT | | LOC_Os05g23910 | Monocot Divergent | Seed/Embryo/Endopserm (Group E) | CCAAT | | LOC_Os01g24460 | Monocot Divergent | Seed/Embryo/Endopserm (Group E) | CCAAT | | LOC_Os01g39850 | Monocot Divergent | Seed/Embryo/Endopserm (Group E),Root_ABA_3 hrs,Root_tZ_6 hrs | CCAAT | | LOC_Os01g01290 | Monocot Divergent | Seed/Embryo/Endopserm (Group E) | CCAAT | | LOC_Os01g01470 | Monocot Divergent | Seed/Embryo/Endopserm (Group E) | NAC | | LOC_Os11g31330 | Monocot Divergent | Seed/Embryo/Endopserm (Group E) | NAC | | LOC_Os11g31380 | Monocot Divergent | Seed/Embryo/Endopserm (Group E) | NAC | | LOC_Os11g31360 | Monocot Divergent | Seed/Embryo/Endopserm (Group E) | NAC | | LOC_Os11g31340 | Monocot Divergent | Seed/Embryo/Endopserm (Group E) | NAC | | LOC_Os07g15540 | Monocot Divergent | Seed/Embryo/Endopserm (Group E),Submergance | Orphans | | LOC_Os02g09910 | Monocot Divergent | Seed/Embryo/Endopserm (Group E) | PHD | | LOC_Os02g51300 | Monocot Divergent | Ubiquitous (Group F),Root_ABA_3 hrs,Root_JA_30 mins,Root_JA_1 hr | AP2-EREBP | | LOC_Os11g34200 | Monocot Divergent | Ubiquitous (Group F) | CCAAT | | LOC_Os07g28430 | Monocot Divergent | Ubiquitous (Group F) | GRF | | LOC_Os02g08544 | Monocot Divergent | Ubiquitous (Group F) | HB | | LOC_Os01g14370 | Monocot Divergent | Ubiquitous (Group F) | TAZ | | LOC_Os04g47690 | Monocot Divergent | Ubiquitous (Group F) | HMG | | LOC_Os01g46700 | Monocot Divergent | Ubiquitous (Group F) | SET | | LOC_Os04g28000 | Rice divergent |  | ABI3VP1 | | LOC_Os10g17630 | Rice divergent |  | ABI3VP1 | | LOC_Os07g12820 | Rice divergent |  | ABI3VP1 | | LOC_Os10g07060 | Rice divergent |  | ABI3VP1 | | LOC_Os07g08600 | Rice divergent |  | ABI3VP1 | | LOC_Os07g08540 | Rice divergent |  | ABI3VP1 | | LOC_Os07g08530 | Rice divergent |  | ABI3VP1 | | LOC_Os04g36730 | Rice divergent |  | Alfin-like | | LOC_Os08g43210 | Rice divergent | Submergance,Root_ABA_1 hr,Root_ABA_3 hrs,Root_JA_1 hr,Root_JA_3 hrs | AP2-EREBP | | LOC_Os06g06970 | Rice divergent |  | AP2-EREBP | | LOC_Os06g42990 | Rice divergent |  | AP2-EREBP | | LOC_Os09g11480 | Rice divergent |  | AP2-EREBP | | LOC_Os09g11460 | Rice divergent |  | AP2-EREBP | | LOC_Os12g41060 | Rice divergent |  | AP2-EREBP | | LOC_Os02g34270 | Rice divergent |  | AP2-EREBP | | LOC_Os02g34260 | Rice divergent | Submergance | AP2-EREBP | | LOC_Os07g08520 | Rice divergent |  | ARF | | LOC_Os12g40630 | Rice divergent |  | bHLH | | LOC_Os11g39000 | Rice divergent |  | bHLH | | LOC_Os04g10260 | Rice divergent |  | bZIP | | LOC_Os08g43600 | Rice divergent |  | bZIP | | LOC_Os03g47200 | Rice divergent |  | bZIP | | LOC_Os06g01340 | Rice divergent |  | C2C2-CO-like | | LOC_Os12g38940 | Rice divergent |  | C2H2 | | LOC_Os03g40710 | Rice divergent |  | C2H2 | | LOC_Os09g03500 | Rice divergent |  | C2H2 | | LOC_Os07g39970 | Rice divergent |  | C2H2 | | LOC_Os04g41060 | Rice divergent |  | C3H | | LOC_Os09g13530 | Rice divergent |  | C3H | | LOC_Os07g04580 | Rice divergent |  | C3H | | LOC_Os12g03554 | Rice divergent |  | C3H | | LOC_Os08g38370 | Rice divergent |  | C3H | | LOC_Os07g04650 | Rice divergent |  | C3H | | LOC_Os04g48375 | Rice divergent | Root_JA_6 hrs | C3H | | LOC_Os06g32720 | Rice divergent |  | C3H | | LOC_Os06g32860 | Rice divergent |  | C3H | | LOC_Os03g02160 | Rice divergent |  | C3H | | LOC_Os08g09690 | Rice divergent |  | CCAAT | | LOC_Os04g09560 | Rice divergent |  | CPP | | LOC_Os07g17160 | Rice divergent |  | EIL | | LOC_Os07g12210 | Rice divergent |  | EIL | | LOC_Os09g08460 | Rice divergent |  | FAR1 | | LOC_Os11g12490 | Rice divergent |  | FAR1 | | LOC_Os12g24400 | Rice divergent |  | FAR1 | | LOC_Os06g36970 | Rice divergent |  | FAR1 | | LOC_Os09g25510 | Rice divergent |  | FAR1 | | LOC_Os09g11390 | Rice divergent |  | FAR1 | | LOC_Os05g40680 | Rice divergent |  | FAR1 | | LOC_Os03g40160 | Rice divergent |  | FAR1 | | LOC_Os04g49380 | Rice divergent |  | FAR1 | | LOC_Os04g30870 | Rice divergent |  | FAR1 | | LOC_Os02g01950 | Rice divergent |  | FAR1 | | LOC_Os05g03090 | Rice divergent |  | FAR1 | | LOC_Os07g27800 | Rice divergent | BPH | FAR1 | | LOC_Os07g27770 | Rice divergent |  | FAR1 | | LOC_Os08g32440 | Rice divergent |  | FAR1 | | LOC_Os02g01860 | Rice divergent | Low temperature | FAR1 | | LOC_Os11g15755 | Rice divergent |  | FAR1 | | LOC_Os09g14040 | Rice divergent |  | FAR1 | | LOC_Os05g39790 | Rice divergent |  | FAR1 | | LOC_Os08g15510 | Rice divergent |  | FAR1 | | LOC_Os08g14880 | Rice divergent |  | FAR1 | | LOC_Os02g28180 | Rice divergent |  | FAR1 | | LOC_Os11g02964 | Rice divergent |  | FAR1 | | LOC_Os10g19270 | Rice divergent |  | FAR1 | | LOC_Os04g22990 | Rice divergent |  | FAR1 | | LOC_Os10g06860 | Rice divergent |  | FAR1 | | LOC_Os08g16370 | Rice divergent |  | FAR1 | | LOC_Os04g54880 | Rice divergent |  | FAR1 | | LOC_Os06g49550 | Rice divergent |  | FAR1 | | LOC_Os09g32750 | Rice divergent |  | FHA | | LOC_Os10g39550 | Rice divergent |  | G2-like | | LOC_Os02g07170 | Rice divergent | Drought ,Root_JA_6 hrs | G2-like | | LOC_Os12g06540 | Rice divergent |  | GRAS | | LOC_Os11g11600 | Rice divergent |  | GRAS | | LOC_Os11g47910 | Rice divergent |  | GRAS | | LOC_Os12g04200 | Rice divergent |  | GRAS | | LOC_Os11g04400 | Rice divergent |  | GRAS | | LOC_Os01g67670 | Rice divergent |  | GRAS | | LOC_Os05g31380 | Rice divergent |  | GRAS | | LOC_Os05g31420 | Rice divergent |  | GRAS | | LOC_Os07g24350 | Rice divergent |  | HB | | LOC_Os05g25600 | Rice divergent |  | HB | | LOC_Os07g34880 | Rice divergent |  | HB | | LOC_Os03g55990 | Rice divergent |  | HB | | LOC_Os08g19590 | Rice divergent |  | HB | | LOC_Os01g39180 | Rice divergent |  | LOB | | LOC_Os01g39040 | Rice divergent |  | LOB | | LOC_Os11g01550 | Rice divergent | BPH,Root_ABA_30 mins,Root_ABA_1 hr,Root_ABA_3 hrs | LOB | | LOC_Os02g48270 | Rice divergent |  | LOB | | LOC_Os04g31804 | Rice divergent |  | MADS | | LOC_Os01g67890 | Rice divergent |  | MADS | | LOC_Os03g14850 | Rice divergent | Low temperature,BPH | MADS | | LOC_Os05g41166 | Rice divergent |  | MYB | | LOC_Os06g40330 | Rice divergent |  | MYB | | LOC_Os02g49250 | Rice divergent | RSV | MYB | | LOC_Os01g12700 | Rice divergent |  | MYB-related | | LOC_Os10g20990 | Rice divergent |  | MYB-related | | LOC_Os03g63890 | Rice divergent |  | MYB-related | | LOC_Os03g62100 | Rice divergent |  | MYB-related | | LOC_Os02g30700 | Rice divergent |  | MYB-related | | LOC_Os04g27410 | Rice divergent |  | MYB-related | | LOC_Os03g59730 | Rice divergent |  | NAC | | LOC_Os03g01870 | Rice divergent |  | NAC | | LOC_Os04g59470 | Rice divergent |  | NAC | | LOC_Os10g25640 | Rice divergent |  | NAC | | LOC_Os10g26240 | Rice divergent |  | NAC | | LOC_Os03g62470 | Rice divergent |  | NAC | | LOC_Os02g28660 | Rice divergent |  | Orphans | | LOC_Os03g12440 | Rice divergent |  | PLATZ | | LOC_Os09g27190 | Rice divergent |  | RWP-RK | | LOC_Os10g25250 | Rice divergent |  | Tify | | LOC_Os02g49970 | Rice divergent |  | Tify | | LOC_Os04g04300 | Rice divergent |  | WRKY | | LOC_Os11g45850 | Rice divergent |  | WRKY | | LOC_Os11g45920 | Rice divergent |  | WRKY | | LOC_Os08g09840 | Rice divergent |  | WRKY | | LOC_Os06g23030 | Rice divergent |  | zf-HD | | LOC_Os04g35880 | Rice divergent |  | DDT | | LOC_Os12g07200 | Rice divergent |  | Jumonji | | LOC_Os01g42270 | Rice divergent |  | LUG | | LOC_Os08g08210 | Rice divergent |  | SET | | LOC_Os01g44990 | Rice divergent |  | SNF2 | | LOC_Os03g48120 | Rice divergent |  | TRAF | | LOC_Os08g41230 | Rice divergent |  | TRAF | | LOC_Os04g53430 | Rice divergent |  | TRAF | | LOC_Os05g44540 | Rice divergent |  | TRAF | | LOC_Os10g29260 | Rice divergent |  | TRAF | | LOC_Os10g29120 | Rice divergent |  | TRAF | | LOC_Os10g29190 | Rice divergent |  | TRAF | | LOC_Os10g29440 | Rice divergent |  | TRAF | | LOC_Os05g44530 | Rice divergent |  | TRAF | | LOC_Os11g24550 | Rice divergent |  | TRAF | | LOC_Os11g45560 | Rice divergent |  | TRAF | | LOC_Os06g45770 | Rice divergent |  | TRAF | | LOC_Os11g41290 | Rice divergent |  | TRAF | | LOC_Os10g28840 | Rice divergent |  | TRAF | | LOC_Os11g41300 | Rice divergent |  | TRAF | | LOC_Os10g28975 | Rice divergent |  | TRAF | | LOC_Os10g28870 | Rice divergent |  | TRAF | | LOC_Os11g40490 | Rice divergent |  | TRAF | | LOC_Os02g20600 | Rice divergent |  | TRAF | | LOC_Os10g30040 | Rice divergent |  | TRAF | | LOC_Os08g03510 | Rice divergent |  | TRAF | | LOC_Os08g03500 | Rice divergent |  | TRAF | | LOC_Os08g03480 | Rice divergent |  | TRAF | | LOC_Os08g25240 | Rice divergent |  | TRAF | | LOC_Os11g40220 | Rice divergent |  | TRAF | | LOC_Os08g40460 | Rice divergent |  | TRAF | | LOC_Os06g42630 | Monocot Divergent |  | ABI3VP1 | | LOC_Os04g27960 | Monocot Divergent |  | ABI3VP1 | | LOC_Os01g52540 | Monocot Divergent |  | ABI3VP1 | | LOC_Os07g17230 | Monocot Divergent |  | ABI3VP1 | | LOC_Os08g06120 | Monocot Divergent | XOO | ABI3VP1 | | LOC_Os03g06850 | Monocot Divergent | BPH,Root_JA_3 hrs | ABI3VP1 | | LOC_Os03g08620 | Monocot Divergent |  | ABI3VP1 | | LOC_Os10g38000 | Monocot Divergent |  | AP2-EREBP | | LOC_Os09g35010 | Monocot Divergent | Low temperature,RSV,Root_JA_30 mins,Root_JA_1 hr | AP2-EREBP | | LOC_Os09g35030 | Monocot Divergent | Low temperature,Root_ABA_1 hr,Root_IAA_3 hrs,Root_JA_30 mins,Root_JA_1 hr | AP2-EREBP | | LOC_Os08g43200 | Monocot Divergent | Submergance | AP2-EREBP | | LOC_Os09g35020 | Monocot Divergent |  | AP2-EREBP | | LOC_Os05g39590 | Monocot Divergent |  | AP2-EREBP | | LOC_Os10g22600 | Monocot Divergent | Root_ABA_30 mins | AP2-EREBP | | LOC_Os09g20350 | Monocot Divergent | Root_ABA_30 mins | AP2-EREBP | | LOC_Os08g31580 | Monocot Divergent | Root_ABA_15 mins,Root_ABA_30 mins,Root_ABA_1 hr,Root_ABA_3 hrs,Shoot_ABA_1 and 3 hrs,Shoot_ABA_6 and 12 hrs,Root_tZ_6 hrs | AP2-EREBP | | LOC_Os04g52090 | Monocot Divergent | Low temperature,Root_ABA_30 mins,Root_ABA_1 hr,Root_ABA_3 hrs,Root_JA_30 mins,Root_JA_6 hrs | AP2-EREBP | | LOC_Os07g12510 | Monocot Divergent | Root_ABA_3 hrs,Root_JA_1 hr | AP2-EREBP | | LOC_Os03g08490 | Monocot Divergent | Root_JA_1 hr,Shoot_JA_6 hrs and 12 hrs | AP2-EREBP | | LOC_Os03g08500 | Monocot Divergent |  | AP2-EREBP | | LOC_Os07g47790 | Monocot Divergent | Low temperature | AP2-EREBP | | LOC_Os03g08460 | Monocot Divergent |  | AP2-EREBP | | LOC_Os03g08470 | Monocot Divergent |  | AP2-EREBP | | LOC_Os07g42510 | Monocot Divergent | Root_JA_1 hr,Root_JA_6 hrs | AP2-EREBP | | LOC_Os12g41030 | Monocot Divergent |  | AP2-EREBP | | LOC_Os08g27220 | Monocot Divergent |  | AP2-EREBP | | LOC_Os05g37640 | Monocot Divergent |  | AP2-EREBP | | LOC_Os06g43220 | Monocot Divergent |  | AP2-EREBP | | LOC_Os01g59780 | Monocot Divergent |  | AP2-EREBP | | LOC_Os11g19060 | Monocot Divergent | Submergance | AP2-EREBP | | LOC_Os07g03250 | Monocot Divergent |  | AP2-EREBP | | LOC_Os08g07440 | Monocot Divergent |  | AP2-EREBP | | LOC_Os10g26590 | Monocot Divergent |  | AP2-EREBP | | LOC_Os01g04800 | Monocot Divergent | Root_JA_15 mins,Root_JA_30 mins,Root_JA_1 hr,Root_JA_3 hrs,Root_JA_6 hrs | AP2-EREBP | | LOC_Os01g04750 | Monocot Divergent |  | AP2-EREBP | | LOC_Os01g13520 | Monocot Divergent |  | ARF | | LOC_Os04g59430 | Monocot Divergent |  | ARF | | LOC_Os06g43910 | Monocot Divergent |  | ARR-B | | LOC_Os01g56690 | Monocot Divergent |  | bHLH | | LOC_Os02g49480 | Monocot Divergent | Root_ABA_30 mins | bHLH | | LOC_Os06g08500 | Monocot Divergent |  | bHLH | | LOC_Os04g28280 | Monocot Divergent |  | bHLH | | LOC_Os06g30090 | Monocot Divergent |  | bHLH | | LOC_Os06g10820 | Monocot Divergent |  | bHLH | | LOC_Os02g39140 | Monocot Divergent |  | bHLH | | LOC_Os04g41229 | Monocot Divergent |  | bHLH | | LOC_Os12g40730 | Monocot Divergent |  | bHLH | | LOC_Os12g40710 | Monocot Divergent |  | bHLH | | LOC_Os05g06520 | Monocot Divergent |  | bHLH | | LOC_Os09g24490 | Monocot Divergent |  | bHLH | | LOC_Os08g33590 | Monocot Divergent |  | bHLH | | LOC_Os03g03000 | Monocot Divergent |  | bHLH | | LOC_Os02g52190 | Monocot Divergent | Root_tZ_1 hr,Root_tZ_3 hrs,Root_tZ_6 hrs | bHLH | | LOC_Os01g39330 | Monocot Divergent | Low temperature,M. oryzae,Root_IAA_3 hrs,Root_JA_6 hrs | bHLH | | LOC_Os02g13670 | Monocot Divergent |  | bHLH | | LOC_Os03g12760 | Monocot Divergent |  | bHLH | | LOC_Os12g40590 | Monocot Divergent |  | bHLH | | LOC_Os01g70310 | Monocot Divergent | Root_ABA_3 hrs | bHLH | | LOC_Os05g38140 | Monocot Divergent |  | bHLH | | LOC_Os02g02480 | Monocot Divergent |  | bHLH | | LOC_Os06g07820 | Monocot Divergent | BPH | BSD | | LOC_Os09g10840 | Monocot Divergent |  | bZIP | | LOC_Os06g41100 | Monocot Divergent |  | bZIP | | LOC_Os08g07970 | Monocot Divergent |  | bZIP | | LOC_Os04g41820 | Monocot Divergent |  | bZIP | | LOC_Os10g38820 | Monocot Divergent | Drought ,XOO,Shoot_ABA_1 and 3 hrs,Shoot_ABA_6 and 12 hrs | bZIP | | LOC_Os03g03550 | Monocot Divergent |  | bZIP | | LOC_Os03g20650 | Monocot Divergent |  | bZIP | | LOC_Os02g58670 | Monocot Divergent |  | bZIP | | LOC_Os06g50480 | Monocot Divergent |  | bZIP | | LOC_Os06g50830 | Monocot Divergent |  | bZIP | | LOC_Os06g50600 | Monocot Divergent |  | bZIP | | LOC_Os03g19375 | Monocot Divergent |  | bZIP | | LOC_Os01g36220 | Monocot Divergent |  | bZIP | | LOC_Os02g09830 | Monocot Divergent | Salt,Root_JA_1 hr | bZIP | | LOC_Os06g42690 | Monocot Divergent |  | bZIP | | LOC_Os07g03220 | Monocot Divergent |  | bZIP | | LOC_Os03g56010 | Monocot Divergent | Root_ABA_3 hrs | bZIP | | LOC_Os03g13614 | Monocot Divergent |  | bZIP | | LOC_Os02g03580 | Monocot Divergent |  | bZIP | | LOC_Os03g59460 | Monocot Divergent | Root_ABA_30 mins,Root_ABA_3 hrs,Shoot_ABA_6 and 12 hrs | bZIP | | LOC_Os07g10890 | Monocot Divergent |  | bZIP | | LOC_Os08g15050 | Monocot Divergent | Root_ABA_15 mins,Root_ABA_30 mins,Root_ABA_1 hr,Root_ABA_3 hrs | C2C2-CO-like | | LOC_Os06g44450 | Monocot Divergent | Root_ABA_30 mins,Root_ABA_1 hr,Root_ABA_3 hrs | C2C2-CO-like | | LOC_Os02g08150 | Monocot Divergent | Root_ABA_3 hrs,Root_tZ_3 hrs,Root_tZ_6 hrs | C2C2-CO-like | | LOC_Os09g06464 | Monocot Divergent | Root_ABA_3 hrs | C2C2-CO-like | | LOC_Os12g39990 | Monocot Divergent |  | C2C2-Dof | | LOC_Os03g42200 | Monocot Divergent |  | C2C2-Dof | | LOC_Os12g38200 | Monocot Divergent |  | C2C2-Dof | | LOC_Os09g29960 | Monocot Divergent |  | C2C2-Dof | | LOC_Os08g38220 | Monocot Divergent |  | C2C2-Dof | | LOC_Os10g35300 | Monocot Divergent | XOO,Shoot_JA_6 hrs and 12 hrs | C2C2-Dof | | LOC_Os10g32070 | Monocot Divergent |  | C2C2-GATA | | LOC_Os11g08410 | Monocot Divergent |  | C2C2-GATA | | LOC_Os03g03850 | Monocot Divergent |  | C2C2-GATA | | LOC_Os05g06340 | Monocot Divergent |  | C2C2-GATA | | LOC_Os01g24070 | Monocot Divergent | Root_JA_1 hr | C2C2-GATA | | LOC_Os12g07120 | Monocot Divergent |  | C2C2-GATA | | LOC_Os03g05480 | Monocot Divergent |  | C2H2 | | LOC_Os03g05690 | Monocot Divergent |  | C2H2 | | LOC_Os07g40080 | Monocot Divergent | Root_JA_1 hr,Root_JA_3 hrs,Root_JA_6 hrs | C2H2 | | LOC_Os03g32230 | Monocot Divergent | Low temperature,M. oryzae,Root_JA_15 mins,Root_JA_30 mins,Root_JA_1 hr,Root_JA_3 hrs,Root_JA_6 hrs | C2H2 | | LOC_Os11g47620 | Monocot Divergent |  | C2H2 | | LOC_Os03g60570 | Monocot Divergent | Drought ,Root_ABA_1 hr,Root_ABA_3 hrs,Root_JA_15 mins,Root_JA_30 mins,Root_JA_1 hr,Root_JA_3 hrs,Root_JA_6 hrs | C2H2 | | LOC_Os03g60560 | Monocot Divergent | Drought ,Root_ABA_30 mins,Root_ABA_1 hr,Root_ABA_3 hrs,Root_JA_15 mins,Root_JA_30 mins,Root_JA_1 hr,Root_JA_3 hrs,Root_JA_6 hrs | C2H2 | | LOC_Os06g20020 | Monocot Divergent |  | C2H2 | | LOC_Os12g38960 | Monocot Divergent |  | C2H2 | | LOC_Os06g51140 | Monocot Divergent |  | C2H2 | | LOC_Os03g49132 | Monocot Divergent |  | C2H2 | | LOC_Os11g25610 | Monocot Divergent |  | C2H2 | | LOC_Os07g40950 | Monocot Divergent | Salt | C2H2 | | LOC_Os01g66570 | Monocot Divergent |  | C2H2 | | LOC_Os12g18150 | Monocot Divergent |  | C2H2 | | LOC_Os09g10980 | Monocot Divergent |  | C2H2 | | LOC_Os10g28330 | Monocot Divergent |  | C2H2 | | LOC_Os01g70870 | Monocot Divergent | RSV | C2H2 | | LOC_Os07g38090 | Monocot Divergent | Root_JA_1 hr | C3H | | LOC_Os12g33090 | Monocot Divergent |  | C3H | | LOC_Os04g35800 | Monocot Divergent |  | C3H | | LOC_Os01g39100 | Monocot Divergent |  | C3H | | LOC_Os07g39440 | Monocot Divergent |  | C3H | | LOC_Os03g18950 | Monocot Divergent |  | C3H | | LOC_Os01g69910 | Monocot Divergent |  | CAMTA | | LOC_Os01g70880 | Monocot Divergent |  | CCAAT | | LOC_Os08g07740 | Monocot Divergent | Root_JA_30 mins,Root_JA_3 hrs,Root_JA_6 hrs | CCAAT | | LOC_Os05g49780 | Monocot Divergent |  | CCAAT | | LOC_Os08g38780 | Monocot Divergent |  | CCAAT | | LOC_Os08g10560 | Monocot Divergent | Root_ABA_3 hrs,Root_JA_1 hr | CCAAT | | LOC_Os06g22670 | Monocot Divergent |  | CPP | | LOC_Os02g17460 | Monocot Divergent |  | CPP | | LOC_Os03g43730 | Monocot Divergent |  | CPP | | LOC_Os04g02140 | Monocot Divergent |  | E2F-DP | | LOC_Os01g48700 | Monocot Divergent |  | E2F-DP | | LOC_Os08g27740 | Monocot Divergent |  | FAR1 | | LOC_Os07g01270 | Monocot Divergent |  | FAR1 | | LOC_Os04g44630 | Monocot Divergent |  | FAR1 | | LOC_Os01g71850 | Monocot Divergent | M. oryzae | FAR1 | | LOC_Os10g34884 | Monocot Divergent |  | FAR1 | | LOC_Os08g20486 | Monocot Divergent |  | FAR1 | | LOC_Os08g33270 | Monocot Divergent |  | FAR1 | | LOC_Os07g18840 | Monocot Divergent |  | FAR1 | | LOC_Os01g16740 | Monocot Divergent |  | FAR1 | | LOC_Os06g13740 | Monocot Divergent |  | FAR1 | | LOC_Os06g13900 | Monocot Divergent |  | FAR1 | | LOC_Os09g01780 | Monocot Divergent |  | FAR1 | | LOC_Os05g25320 | Monocot Divergent |  | FAR1 | | LOC_Os02g18370 | Monocot Divergent |  | FAR1 | | LOC_Os11g11220 | Monocot Divergent |  | FAR1 | | LOC_Os01g06852 | Monocot Divergent |  | FAR1 | | LOC_Os01g16660 | Monocot Divergent |  | FAR1 | | LOC_Os03g37920 | Monocot Divergent |  | FAR1 | | LOC_Os03g21660 | Monocot Divergent | Low temperature | FAR1 | | LOC_Os03g15040 | Monocot Divergent |  | FAR1 | | LOC_Os12g18910 | Monocot Divergent |  | FAR1 | | LOC_Os06g28150 | Monocot Divergent |  | FAR1 | | LOC_Os03g41800 | Monocot Divergent |  | FAR1 | | LOC_Os06g32530 | Monocot Divergent |  | FAR1 | | LOC_Os11g19030 | Monocot Divergent |  | FAR1 | | LOC_Os02g31850 | Monocot Divergent |  | FAR1 | | LOC_Os11g17430 | Monocot Divergent |  | FAR1 | | LOC_Os07g32110 | Monocot Divergent |  | FAR1 | | LOC_Os05g37540 | Monocot Divergent |  | FAR1 | | LOC_Os01g63030 | Monocot Divergent |  | FAR1 | | LOC_Os04g28580 | Monocot Divergent |  | FAR1 | | LOC_Os02g40950 | Monocot Divergent |  | FAR1 | | LOC_Os03g50900 | Monocot Divergent |  | FAR1 | | LOC_Os06g39680 | Monocot Divergent |  | FAR1 | | LOC_Os11g27530 | Monocot Divergent |  | FHA | | LOC_Os06g35140 | Monocot Divergent |  | G2-like | | LOC_Os02g14490 | Monocot Divergent |  | G2-like | | LOC_Os03g03760 | Monocot Divergent |  | G2-like | | LOC_Os08g33750 | Monocot Divergent |  | G2-like | | LOC_Os01g62660 | Monocot Divergent | Submergance | G2-like | | LOC_Os03g40080 | Monocot Divergent |  | GRAS | | LOC_Os06g01620 | Monocot Divergent |  | GRAS | | LOC_Os04g50060 | Monocot Divergent |  | GRAS | | LOC_Os11g47890 | Monocot Divergent |  | GRAS | | LOC_Os11g47870 | Monocot Divergent |  | GRAS | | LOC_Os11g47920 | Monocot Divergent |  | GRAS | | LOC_Os12g04380 | Monocot Divergent |  | GRAS | | LOC_Os11g04590 | Monocot Divergent |  | GRAS | | LOC_Os12g04370 | Monocot Divergent |  | GRAS | | LOC_Os11g04570 | Monocot Divergent |  | GRAS | | LOC_Os05g40710 | Monocot Divergent |  | GRAS | | LOC_Os11g35030 | Monocot Divergent |  | GRF | | LOC_Os02g05640 | Monocot Divergent |  | HB | | LOC_Os04g46350 | Monocot Divergent | ,Root_IAA_15 mins,Root_IAA_30 mins,Root_IAA_1 hr | HB | | LOC_Os06g04870 | Monocot Divergent | ,Root_IAA_15 mins,Root_IAA_30 mins,Root_IAA_1 hr,Root_IAA_3 hrs | HB | | LOC_Os06g04850 | Monocot Divergent |  | HB | | LOC_Os02g35770 | Monocot Divergent | Drought ,Root_ABA_3 hrs,Shoot_ABA_6 and 12 hrs,Root_JA_6 hrs | HB | | LOC_Os06g43860 | Monocot Divergent |  | HB | | LOC_Os03g56110 | Monocot Divergent | Root_IAA_6 hrs | HB | | LOC_Os03g56140 | Monocot Divergent |  | HB | | LOC_Os03g51710 | Monocot Divergent |  | HB | | LOC_Os01g57890 | Monocot Divergent |  | HB | | LOC_Os08g04190 | Monocot Divergent |  | HB | | LOC_Os03g53340 | Monocot Divergent | Drought ,RSV,Root_ABA_1 hr,Root_ABA_3 hrs | HSF | | LOC_Os01g39020 | Monocot Divergent | Drought ,Root_ABA_30 mins,Root_ABA_1 hr,Root_ABA_3 hrs,Shoot_ABA_6 and 12 hrs | HSF | | LOC_Os03g12370 | Monocot Divergent | Low temperature,Root_JA_1 hr | HSF | | LOC_Os01g53220 | Monocot Divergent | Drought ,BPH,Root_ABA_15 mins,Root_ABA_30 mins,Root_ABA_1 hr,Root_ABA_3 hrs,Root_tZ_6 hrs | HSF | | LOC_Os06g13030 | Monocot Divergent |  | LIM | | LOC_Os12g01550 | Monocot Divergent | Root_ABA_30 mins,Root_ABA_1 hr,Root_ABA_3 hrs,Root_IAA_15 mins,Root_IAA_30 mins,Root_IAA_1 hr,Root_IAA_3 hrs | LOB | | LOC_Os03g57670 | Monocot Divergent |  | LOB | | LOC_Os05g03160 | Monocot Divergent | Root_JA_6 hrs | LOB | | LOC_Os07g04170 | Monocot Divergent |  | MADS | | LOC_Os01g11510 | Monocot Divergent |  | MADS | | LOC_Os06g23950 | Monocot Divergent |  | MADS | | LOC_Os08g41960 | Monocot Divergent |  | MADS | | LOC_Os01g69850 | Monocot Divergent |  | MADS | | LOC_Os06g01890 | Monocot Divergent |  | MADS | | LOC_Os01g68560 | Monocot Divergent |  | MADS | | LOC_Os11g12360 | Monocot Divergent |  | MADS | | LOC_Os05g23780 | Monocot Divergent |  | MADS | | LOC_Os05g34160 | Monocot Divergent |  | mTERF | | LOC_Os02g36780 | Monocot Divergent |  | mTERF | | LOC_Os01g74410 | Monocot Divergent |  | MYB | | LOC_Os01g45090 | Monocot Divergent | Root_JA_30 mins,Root_JA_1 hr,Root_JA_3 hrs | MYB | | LOC_Os10g35660 | Monocot Divergent | BPH | MYB | | LOC_Os03g26130 | Monocot Divergent |  | MYB | | LOC_Os07g44090 | Monocot Divergent |  | MYB | | LOC_Os03g25550 | Monocot Divergent |  | MYB | | LOC_Os06g14670 | Monocot Divergent | Drought ,RSV,Root_ABA_30 mins,Root_ABA_1 hr,Root_ABA_3 hrs,Shoot_ABA_1 and 3 hrs,Shoot_ABA_6 and 12 hrs | MYB | | LOC_Os02g49986 | Monocot Divergent | Root_ABA_30 mins,Root_ABA_1 hr,Root_ABA_3 hrs,Shoot_ABA_1 and 3 hrs,Shoot_ABA_6 and 12 hrs | MYB | | LOC_Os04g50680 | Monocot Divergent |  | MYB | | LOC_Os11g07890 | Monocot Divergent | Root_JA_30 mins,Root_JA_1 hr | MYB | | LOC_Os06g10350 | Monocot Divergent |  | MYB | | LOC_Os08g34960 | Monocot Divergent |  | MYB | | LOC_Os03g13310 | Monocot Divergent |  | MYB | | LOC_Os06g43090 | Monocot Divergent | Low temperature,Shoot_ABA_6 and 12 hrs | MYB | | LOC_Os02g09480 | Monocot Divergent | Low temperature,Shoot_ABA_1 and 3 hrs,Root_IAA_15 mins,Root_JA_1 hr | MYB | | LOC_Os02g17190 | Monocot Divergent |  | MYB-related | | LOC_Os10g30690 | Monocot Divergent |  | MYB-related | | LOC_Os05g02420 | Monocot Divergent | Shoot_JA_6 hrs and 12 hrs | MYB-related | | LOC_Os01g07430 | Monocot Divergent |  | MYB-related | | LOC_Os06g14700 | Monocot Divergent |  | MYB-related | | LOC_Os06g14710 | Monocot Divergent |  | MYB-related | | LOC_Os03g19630 | Monocot Divergent |  | MYB-related | | LOC_Os06g14010 | Monocot Divergent |  | MYB-related | | LOC_Os03g25304 | Monocot Divergent |  | MYB-related | | LOC_Os01g11200 | Monocot Divergent |  | MYB-related | | LOC_Os07g25370 | Monocot Divergent |  | MYB-related | | LOC_Os01g09760 | Monocot Divergent |  | MYB-related | | LOC_Os01g06320 | Monocot Divergent |  | MYB-related | | LOC_Os05g07010 | Monocot Divergent | Low temperature,RSV,Root_JA_15 mins,Root_JA_30 mins,Root_JA_1 hr | MYB-related | | LOC_Os10g41260 | Monocot Divergent |  | MYB-related | | LOC_Os08g04840 | Monocot Divergent |  | MYB-related | | LOC_Os05g37050 | Monocot Divergent |  | MYB-related | | LOC_Os01g44370 | Monocot Divergent |  | MYB-related | | LOC_Os02g57270 | Monocot Divergent |  | MYB-related | | LOC_Os06g36480 | Monocot Divergent |  | NAC | | LOC_Os05g10620 | Monocot Divergent | Root_IAA_3 hrs,Root_JA_1 hr | NAC | | LOC_Os08g02160 | Monocot Divergent |  | NAC | | LOC_Os02g41450 | Monocot Divergent | BPH,Root_tZ_30 mins,Root_tZ_1 hr,Root_tZ_3 hrs,Root_tZ_6 hrs,Root_JA_30 mins | NAC | | LOC_Os06g01480 | Monocot Divergent |  | NAC | | LOC_Os01g15640 | Monocot Divergent |  | NAC | | LOC_Os09g24560 | Monocot Divergent |  | NAC | | LOC_Os08g33670 | Monocot Divergent |  | NAC | | LOC_Os01g70110 | Monocot Divergent | Root_JA_1 hr | NAC | | LOC_Os09g33490 | Monocot Divergent | Root_IAA_30 mins,Root_IAA_1 hr,Root_IAA_3 hrs,Root_tZ_6 hrs,Root_JA_15 mins,Root_JA_30 mins,Root_JA_6 hrs | NAC | | LOC_Os08g23880 | Monocot Divergent |  | NAC | | LOC_Os01g59640 | Monocot Divergent |  | NAC | | LOC_Os10g26270 | Monocot Divergent |  | NAC | | LOC_Os10g25620 | Monocot Divergent |  | NAC | | LOC_Os10g27360 | Monocot Divergent |  | NAC | | LOC_Os10g27390 | Monocot Divergent |  | NAC | | LOC_Os12g07790 | Monocot Divergent |  | NAC | | LOC_Os12g22940 | Monocot Divergent |  | NAC | | LOC_Os12g23090 | Monocot Divergent |  | NAC | | LOC_Os12g22630 | Monocot Divergent |  | NAC | | LOC_Os11g04960 | Monocot Divergent |  | NAC | | LOC_Os10g09820 | Monocot Divergent |  | NAC | | LOC_Os09g12380 | Monocot Divergent |  | NAC | | LOC_Os03g61319 | Monocot Divergent |  | NAC | | LOC_Os03g61249 | Monocot Divergent |  | NAC | | LOC_Os03g21870 | Monocot Divergent | RSV | OFP | | LOC_Os07g48150 | Monocot Divergent | Root_tZ_6 hrs | OFP | | LOC_Os10g29610 | Monocot Divergent |  | OFP | | LOC_Os04g37510 | Monocot Divergent |  | OFP | | LOC_Os07g15770 | Monocot Divergent |  | Orphans | | LOC_Os01g34610 | Monocot Divergent | Submergance | Orphans | | LOC_Os11g01074 | Monocot Divergent |  | Orphans | | LOC_Os12g10660 | Monocot Divergent |  | Orphans | | LOC_Os12g40490 | Monocot Divergent |  | Orphans | | LOC_Os03g42820 | Monocot Divergent |  | Orphans | | LOC_Os04g36070 | Monocot Divergent | Root_tZ_30 mins,Root_tZ_1 hr,Root_tZ_3 hrs,Root_tZ_6 hrs,Shoot_tZ_3 and 6 hr | Orphans | | LOC_Os02g35180 | Monocot Divergent | Root_tZ_30 mins,Root_tZ_1 hr,Root_tZ_3 hrs,Root_tZ_6 hrs | Orphans | | LOC_Os08g26990 | Monocot Divergent |  | Orphans | | LOC_Os08g28950 | Monocot Divergent |  | Orphans | | LOC_Os08g28900 | Monocot Divergent |  | Orphans | | LOC_Os07g26720 | Monocot Divergent |  | Orphans | | LOC_Os04g57720 | Monocot Divergent | Root_tZ_15 mins,Root_tZ_30 mins,Root_tZ_1 hr,Root_tZ_3 hrs,Root_tZ_6 hrs,Root_JA_1 hr | Orphans | | LOC_Os04g44280 | Monocot Divergent |  | Orphans | | LOC_Os07g37140 | Monocot Divergent |  | Orphans | | LOC_Os10g30880 | Monocot Divergent |  | Orphans | | LOC_Os10g42410 | Monocot Divergent | Root_JA_15 mins,Root_JA_30 mins,Root_JA_1 hr,Root_JA_3 hrs,Root_JA_6 hrs,Shoot_JA_6 hrs and 12 hrs | PLATZ | | LOC_Os02g10000 | Monocot Divergent |  | PLATZ | | LOC_Os02g44260 | Monocot Divergent |  | PLATZ | | LOC_Os09g02790 | Monocot Divergent |  | PLATZ | | LOC_Os01g37100 | Monocot Divergent |  | RWP-RK | | LOC_Os04g56170 | Monocot Divergent |  | SBP | | LOC_Os11g30370 | Monocot Divergent |  | SBP | | LOC_Os06g45310 | Monocot Divergent | Root_tZ_1 hr,Root_tZ_3 hrs,Root_tZ_6 hrs | SBP | | LOC_Os02g07780 | Monocot Divergent |  | SBP | | LOC_Os01g18850 | Monocot Divergent | Root_JA_6 hrs | SBP | | LOC_Os09g24480 | Monocot Divergent |  | TCP | | LOC_Os08g33530 | Monocot Divergent |  | TCP | | LOC_Os12g42190 | Monocot Divergent |  | TCP | | LOC_Os10g25230 | Monocot Divergent | Low temperature,RSV,Root_IAA_1 hr,Root_tZ_3 hrs,Root_JA_15 mins,Root_JA_30 mins,Root_JA_1 hr,Root_JA_3 hrs,Root_JA_6 hrs,Shoot_JA_3 and 6 hrs,Shoot_JA_6 hrs and 12 hrs | Tify | | LOC_Os03g08330 | Monocot Divergent | Low temperature,RSV,Root_ABA_3 hrs,Root_IAA_1 hr,Root_IAA_3 hrs,Root_tZ_3 hrs,Root_tZ_6 hrs,Root_JA_15 mins,Root_JA_30 mins,Root_JA_1 hr,Root_JA_3 hrs,Root_JA_6 hrs,Shoot_JA_3 and 6 hrs,Shoot_JA_6 hrs and 12 hrs | Tify | | LOC_Os03g08310 | Monocot Divergent | Low temperature,RSV,Root_IAA_1 hr,Root_IAA_3 hrs,Root_IAA_6 hrs,Root_BL_3 hrs,Root_JA_15 mins,Root_JA_30 mins,Root_JA_1 hr,Root_JA_3 hrs,Root_JA_6 hrs,Shoot_JA_3 and 6 hrs,Shoot_JA_6 hrs and 12 hrs | Tify | | LOC_Os09g26780 | Monocot Divergent | Root_JA_15 mins,Root_JA_30 mins,Root_JA_1 hr,Root_JA_3 hrs,Root_JA_6 hrs,Shoot_JA_6 hrs and 12 hrs | Tify | | LOC_Os10g25290 | Monocot Divergent | Low temperature,Root_ABA_3 hrs,Root_IAA_1 hr,Root_IAA_3 hrs,Root_tZ_3 hrs,Root_tZ_6 hrs,Root_JA_15 mins,Root_JA_30 mins,Root_JA_1 hr,Root_JA_3 hrs,Root_JA_6 hrs,Shoot_JA_3 and 6 hrs,Shoot_JA_6 hrs and 12 hrs | Tify | | LOC_Os03g08320 | Monocot Divergent | Low temperature,M. oryzae,Root_ABA_3 hrs,Root_IAA_1 hr,Root_IAA_3 hrs,Root_IAA_6 hrs,Root_tZ_6 hrs,Root_JA_15 mins,Root_JA_30 mins,Root_JA_1 hr,Root_JA_3 hrs,Root_JA_6 hrs,Shoot_JA_6 hrs and 12 hrs | Tify | | LOC_Os01g70230 | Monocot Divergent | Salt | Trihelix | | LOC_Os02g43300 | Monocot Divergent | Root_ABA_3 hrs,Root_tZ_3 hrs,Root_tZ_6 hrs | Trihelix | | LOC_Os04g45750 | Monocot Divergent |  | Trihelix | | LOC_Os03g18330 | Monocot Divergent |  | Trihelix | | LOC_Os05g45230 | Monocot Divergent |  | WRKY | | LOC_Os05g50700 | Monocot Divergent | Low temperature | WRKY | | LOC_Os01g43550 | Monocot Divergent | Root_ABA_1 hr,Root_ABA_3 hrs | WRKY | | LOC_Os03g63810 | Monocot Divergent |  | WRKY | | LOC_Os02g26430 | Monocot Divergent | Low temperature,Root_ABA_30 mins,Root_ABA_1 hr,Root_JA_1 hr,Root_JA_6 hrs | WRKY | | LOC_Os07g40570 | Monocot Divergent |  | WRKY | | LOC_Os02g43560 | Monocot Divergent |  | WRKY | | LOC_Os09g25070 | Monocot Divergent | RSV,Root_JA_30 mins,Root_JA_1 hr | WRKY | | LOC_Os01g40430 | Monocot Divergent |  | WRKY | | LOC_Os01g62514 | Monocot Divergent |  | WRKY | | LOC_Os12g02470 | Monocot Divergent |  | WRKY | | LOC_Os11g02540 | Monocot Divergent |  | WRKY | | LOC_Os12g02420 | Monocot Divergent |  | WRKY | | LOC_Os11g02480 | Monocot Divergent | Shoot_ABA_6 and 12 hrs | WRKY | | LOC_Os03g20550 | Monocot Divergent | Root_JA_30 mins,Root_JA_1 hr | WRKY | | LOC_Os07g27670 | Monocot Divergent | M. oryzae | WRKY | | LOC_Os01g60600 | Monocot Divergent | Low temperature | WRKY | | LOC_Os01g60520 | Monocot Divergent |  | WRKY | | LOC_Os08g09810 | Monocot Divergent |  | WRKY | | LOC_Os08g09800 | Monocot Divergent |  | WRKY | | LOC_Os08g09900 | Monocot Divergent | BPH | WRKY | | LOC_Os01g60540 | Monocot Divergent |  | WRKY | | LOC_Os02g47770 | Monocot Divergent |  | zf-HD | | LOC_Os04g35500 | Monocot Divergent |  | zf-HD | | LOC_Os03g53150 | Monocot Divergent |  | AUX_IAA | | LOC_Os09g35870 | Monocot Divergent | Root_IAA_15 mins,Root_IAA_30 mins,Root_IAA_1 hr,Root_IAA_3 hrs,Root_IAA_6 hrs | AUX_IAA | | LOC_Os07g08460 | Monocot Divergent | Root_IAA_15 mins,Root_IAA_30 mins,Root_IAA_1 hr,Root_IAA_3 hrs | AUX_IAA | | LOC_Os01g09450 | Monocot Divergent | Root_JA_3 hrs,Root_JA_6 hrs | AUX_IAA | | LOC_Os05g09480 | Monocot Divergent | Root_tZ_1 hr,Root_JA_15 mins,Root_JA_30 mins,Root_JA_1 hr,Root_JA_3 hrs,Root_JA_6 hrs,Shoot_JA_6 hrs and 12 hrs | AUX_IAA | | LOC_Os11g11410 | Monocot Divergent |  | AUX_IAA | | LOC_Os06g24850 | Monocot Divergent |  | AUX_IAA | | LOC_Os08g01780 | Monocot Divergent | BPH | AUX_IAA | | LOC_Os03g10810 | Monocot Divergent | BPH | GNAT | | LOC_Os02g44930 | Monocot Divergent |  | HMG | | LOC_Os02g46930 | Monocot Divergent | Root_JA_3 hrs,Root_JA_6 hrs | Jumonji | | LOC_Os09g22540 | Monocot Divergent |  | Jumonji | | LOC_Os02g01940 | Monocot Divergent |  | Jumonji | | LOC_Os03g64300 | Monocot Divergent |  | LUG | | LOC_Os02g48810 | Monocot Divergent |  | PHD | | LOC_Os03g15990 | Monocot Divergent |  | PHD | | LOC_Os11g12650 | Monocot Divergent | Low temperature | PHD | | LOC_Os01g66070 | Monocot Divergent |  | PHD | | LOC_Os01g11960 | Monocot Divergent | Root_ABA_3 hrs | PHD | | LOC_Os11g32900 | Monocot Divergent |  | RB | | LOC_Os10g41620 | Monocot Divergent |  | SNF2 | | LOC_Os01g65850 | Monocot Divergent |  | SNF2 | | LOC_Os10g36810 | Monocot Divergent |  | SWI_SNF-BAF60b | | LOC_Os04g40630 | Monocot Divergent | Shoot_IAA_12 hrs,Root_JA_3 hrs,Root_JA_6 hrs,Shoot_JA_6 hrs and 12 hrs | TRAF | | LOC_Os09g16870 | Monocot Divergent |  | TRAF | | LOC_Os09g16850 | Monocot Divergent |  | TRAF | | LOC_Os08g41240 | Monocot Divergent |  | TRAF | | LOC_Os08g41120 | Monocot Divergent |  | TRAF | | LOC_Os09g06890 | Monocot Divergent |  | TRAF | | LOC_Os08g41220 | Monocot Divergent |  | TRAF | | LOC_Os08g41170 | Monocot Divergent |  | TRAF | | LOC_Os08g41180 | Monocot Divergent |  | TRAF | | LOC_Os08g41150 | Monocot Divergent |  | TRAF | | LOC_Os08g40490 | Monocot Divergent |  | TRAF | | LOC_Os04g53390 | Monocot Divergent |  | TRAF | | LOC_Os04g35370 | Monocot Divergent |  | TRAF | | LOC_Os10g29320 | Monocot Divergent |  | TRAF | | LOC_Os10g29360 | Monocot Divergent |  | TRAF | | LOC_Os11g41260 | Monocot Divergent |  | TRAF | | LOC_Os10g28970 | Monocot Divergent | Root_tZ_15 mins,Root_tZ_1 hr | TRAF | | LOC_Os02g52316 | Monocot Divergent |  | TRAF | | LOC_Os10g29920 | Monocot Divergent |  | TRAF | | LOC_Os10g30350 | Monocot Divergent |  | TRAF | | LOC_Os10g29050 | Monocot Divergent |  | TRAF | | LOC_Os06g45720 | Monocot Divergent |  | TRAF | | LOC_Os06g14060 | Monocot Divergent |  | TRAF | | LOC_Os08g13250 | Monocot Divergent |  | TRAF | | LOC_Os08g13090 | Monocot Divergent |  | TRAF | | LOC_Os08g13070 | Monocot Divergent |  | TRAF | | LOC_Os08g13060 | Monocot Divergent |  | TRAF | | LOC_Os08g03530 | Monocot Divergent |  | TRAF | | LOC_Os12g04410 | Monocot Divergent |  | TRAF | | LOC_Os11g04600 | Monocot Divergent |  | TRAF | |
|  |

**Table S6.** List and information of abiotic stress-induced TF genes.

| **Locus_ID Abiotic stress type Fold change p -value Biotic stress Family RGAP 7 Annotation Previous reported function**  **response** |
| --- |
| | LOC_Os02g43330 | Drought | 5.728 | 2.5400E-18 | XOO | HB | homeobox associated leucine zipper, putative, expressed | panicle development | | --- | --- | --- | --- | --- | --- | --- | --- | | LOC_Os06g35960 | Drought | 5.076 | 2.6600E-15 |  | HSF | HSF-type DNA-binding domain containing protein, expressed |  | | LOC_Os08g36920 | Drought | 5.022 | 2.0400E-10 | RSV | AP2-EREBP | AP2 domain containing protein, expressed |  | | LOC_Os01g39020 | Drought | 3.852 | 2.2300E-18 |  | HSF | HSF-type DNA-binding domain containing protein, expressed | Drought and salinity tolerance. | | LOC_Os03g60560 | Drought | 3.825 | 8.0100E-11 |  | C2H2 | ZOS3-21 - C2H2 zinc finger protein, expressed | Salinity tolerance. | | LOC_Os04g45810 | Drought | 3.562 | 5.1500E-14 |  | HB | homeobox associated leucine zipper, putative, expressed | Drought and salinity tolerance. | | LOC_Os01g64730 | Drought | 3.502 | 2.1400E-17 |  | bZIP | bZIP transcription factor domain containing protein, expressed | Drought and salinity tolerance. | | LOC_Os02g34970 | Drought | 3.47 | 3.9100E-07 |  | NAC | no apical meristem protein, putative, expressed |  | | LOC_Os02g52670 | Drought | 3.361 | 3.7800E-09 |  | AP2-EREBP | AP2 domain containing protein, expressed |  | | LOC_Os06g03670 | Drought | 3.32 | 1.6600E-08 | RSV | AP2-EREBP | dehydration-responsive element-binding protein, putative, expressed | Cold, drought and salinity tolerance. | | LOC_Os02g13800 | Drought | 3.244 | 5.3300E-12 |  | HSF | HSF-type DNA-binding domain containing protein, expressed |  | | LOC_Os01g63980 | Drought | 3.132 | 1.5100E-09 |  | C2H2 | ZOS1-17 - C2H2 zinc finger protein, expressed |  | | LOC_Os07g39960 | Drought | 3.131 | 1.9400E-07 |  | C2H2 | ZOS7-07 - C2H2 zinc finger protein, expressed |  | | LOC_Os11g03300 | Drought | 3.057 | 8.5600E-08 |  | NAC | NAC domain transcription factor, putative, expressed | Grain yield under drought conditions. | | LOC_Os11g45740 | Drought | 2.975 | 3.2000E-10 | XOO | MYB | MYB family transcription factor, putative, expressed | Resistance to brown spot pathogen Cochliobolus miyabeanus. | | LOC_Os02g07170 | Drought | 2.965 | 1.6300E-06 |  | G2-like | MYB family transcription factor, putative, expressed |  | | LOC_Os04g42950 | Drought | 2.913 | 2.4800E-11 |  | MYB | MYB family transcription factor, putative, expressed |  | | LOC_Os05g34830 | Drought | 2.771 | 7.6100E-14 | XOO | NAC | No apical meristem protein, putative, expressed |  | | LOC_Os01g64360 | Drought | 2.733 | 2.4600E-12 |  | MYB | MYB family transcription factor, putative, expressed |  | | LOC_Os02g43820 | Drought | 2.729 | 3.5800E-07 | XOO | AP2-EREBP | AP2 domain containing protein, expressed |  | | LOC_Os03g53340 | Drought | 2.701 | 6.8000E-06 | RSV | HSF | HSF-type DNA-binding domain containing protein, expressed |  | | LOC_Os02g35770 | Drought | 2.697 | 1.6900E-08 |  | HB | homeobox associated leucine zipper, putative, expressed |  | | LOC_Os01g53220 | Drought | 2.674 | 1.8400E-11 | BPH | HSF | HSF-type DNA-binding domain containing protein, expressed |  | | LOC_Os01g53650 | Drought | 2.602 | 3.2100E-05 |  | C3H | zinc finger CCCH type family protein, putative, expressed |  | | LOC_Os09g21180 | Drought | 2.501 | 2.5100E-05 | BPH | HB | homeobox associated leucine zipper, putative, expressed |  | | LOC_Os05g10670 | Drought | 2.468 | 7.1100E-11 |  | C3H | zinc finger CCCH type family protein, putative, expressed | Drought and salinity tolerance. | | LOC_Os07g48450 | Drought | 2.453 | 4.9183E-03 |  | NAC | no apical meristem protein, putative, expressed |  | | LOC_Os04g49450 | Drought | 2.439 | 3.2400E-05 | BPH | MYB-related | MYB family transcription factor, putative, expressed |  | | LOC_Os10g38820 | Drought | 2.397 | 1.7110E-04 | XOO | bZIP | bZIP family transcription factor, putative, expressed |  | | LOC_Os06g07030 | Drought | 2.363 | 1.7400E-08 |  | AP2-EREBP | AP2 domain containing protein, expressed |  | | LOC_Os07g37210 | Drought | 2.265 | 7.8000E-06 | M. grisea | MYB | MYB family transcription factor, putative, expressed |  | | LOC_Os01g46970 | Drought | 2.262 | 2.1500E-11 |  | bZIP | transcription factor, putative, expressed |  | | LOC_Os05g08970 | Drought | 2.226 | 6.9700E-10 |  | HMG | SSRP1-like FACT complex subunit, putative, expressed |  | | LOC_Os06g14670 | Drought | 2.205 | 3.3155E-04 | RSV | MYB | ODORANT1, putative, expressed |  | | LOC_Os03g10210 | Drought | 2.204 | 8.0679E-04 | BPH | HB | homeobox domain containing protein, expressed |  | | LOC_Os05g49420 | Drought | 2.153 | 2.3500E-08 |  | bZIP | transcription factor, putative, expressed |  | | LOC_Os02g46030 | Drought | 2.113 | 2.9900E-05 | M. grisea | MYB-related | MYB family transcription factor, putative, expressed |  | | LOC_Os12g39400 | Drought | 2.11 | 1.9900E-06 | BPH | C2H2 | ZOS12-09 - C2H2 zinc finger protein, expressed | Drought and salinity tolerance. | | LOC_Os03g60570 | Drought | 2.075 | 1.5067E-03 |  | C2H2 | ZOS3-22 - C2H2 zinc finger protein, expressed |  | | LOC_Os02g56120 | Drought | 2.039 | 3.4562E-04 | BPH | AUX_IAA | OsIAA9 - Auxin-responsive Aux/IAA gene family member, expressed |  | | LOC_Os05g45020 | Drought | 2.03 | 1.4647E-04 |  | C3H | zinc finger/CCCH transcription factor, putative, expressed |  | | LOC_Os02g43330 | Salt | 4.422 | 1.6200E-05 | XOO | HB | homeobox associated leucine zipper, putative, expressed | panicle development | | LOC_Os04g42950 | Salt | 3.474 | 4.5800E-05 |  | MYB | MYB family transcription factor, putative, expressed |  | | LOC_Os06g35960 | Salt | 3.368 | 1.3900E-07 |  | HSF | HSF-type DNA-binding domain containing protein, expressed |  | | LOC_Os08g36920 | Salt | 3.287 | 8.5000E-06 | RSV | AP2-EREBP | AP2 domain containing protein, expressed |  | | LOC_Os02g09830 | Salt | 2.568 | 1.3782E-03 |  | bZIP | bZIP transcription factor domain containing protein, expressed |  | | LOC_Os01g39020 | Salt | 2.561 | 3.7300E-05 |  | HSF | HSF-type DNA-binding domain containing protein, expressed | Drought and salinity tolerance. | | LOC_Os03g60560 | Salt | 2.459 | 1.9325E-02 |  | C2H2 | ZOS3-21 - C2H2 zinc finger protein, expressed | Salinity tolerance. | | LOC_Os05g04210 | Salt | 2.403 | 6.0803E-03 |  | MYB | MYB family transcription factor, putative, expressed |  | | LOC_Os06g03670 | Salt | 2.394 | 1.5276E-02 | RSV | AP2-EREBP | dehydration-responsive element-binding protein, putative, expressed | Cold, drought and salinity tolerance. | | LOC_Os04g45810 | Salt | 2.383 | 3.5120E-04 |  | HB | homeobox associated leucine zipper, putative, expressed | Drought and salinity tolerance. | | LOC_Os10g33810 | Salt | 2.253 | 1.6304E-03 |  | MYB | myb-related protein Myb4, putative, expressed |  | | LOC_Os09g21180 | Salt | 2.192 | 8.3741E-03 | BPH | HB | homeobox associated leucine zipper, putative, expressed |  | | LOC_Os07g40950 | Salt | 2.183 | 1.0375E-02 |  | C2H2 | ZOS7-12 - C2H2 zinc finger protein, expressed |  | | LOC_Os01g63980 | Salt | 2.175 | 1.0481E-02 |  | C2H2 | ZOS1-17 - C2H2 zinc finger protein, expressed |  | | LOC_Os02g07170 | Salt | 2.153 | 3.0168E-03 |  | G2-like | MYB family transcription factor, putative, expressed |  | | LOC_Os11g03300 | Salt | 2.122 | 1.1239E-03 |  | NAC | NAC domain transcription factor, putative, expressed | Grain yield under drought conditions. | | LOC_Os05g34830 | Salt | 2.108 | 4.1474E-03 | XOO | NAC | No apical meristem protein, putative, expressed |  | | LOC_Os01g70230 | Salt | 2.104 | 4.7879E-02 |  | Trihelix | transcription factor, putative, expressed |  | | LOC_Os05g07120 | Salt | 2.058 | 3.4087E-02 |  | bHLH | basic helix-loop-helix, putative, expressed |  | | LOC_Os01g64360 | Salt | 2.011 | 1.2759E-03 |  | MYB | MYB family transcription factor, putative, expressed |  | | LOC_Os10g41330 | Low temperature | 6.569 | 3.9400E-26 | BPH | AP2-EREBP | AP2 domain containing protein, expressed |  | | LOC_Os09g28440 | Low temperature | 6.36 | 3.4500E-33 | RSV | AP2-EREBP | AP2 domain containing protein, expressed | Internode elongation. Panicle branching. Tillering. Salinity tolerance. | | LOC_Os08g36920 | Low temperature | 6.007 | 3.5900E-29 | RSV | AP2-EREBP | AP2 domain containing protein, expressed |  | | LOC_Os04g49450 | Low temperature | 5.926 | 9.3700E-37 | BPH | MYB-related | MYB family transcription factor, putative, expressed |  | | LOC_Os06g03670 | Low temperature | 5.881 | 3.6800E-37 | RSV | AP2-EREBP | dehydration-responsive element-binding protein, putative, expressed | Cold, drought and salinity tolerance. | | LOC_Os03g60570 | Low temperature | 5.866 | 4.3000E-39 |  | C2H2 | ZOS3-22 - C2H2 zinc finger protein, expressed |  | | LOC_Os02g52670 | Low temperature | 5.789 | 3.5500E-27 |  | AP2-EREBP | AP2 domain containing protein, expressed |  | | LOC_Os06g39590 | Low temperature | 5.659 | 2.2800E-18 |  | AUX_IAA | OsIAA23 - Auxin-responsive Aux/IAA gene family member, expressed | Root development. Quiescent center identity. Auxin sensitivity. | | LOC_Os02g45450 | Low temperature | 5.179 | 2.3700E-26 | RSV | AP2-EREBP | dehydration-responsive element-binding protein, putative, expressed | | | LOC_Os03g60560 | Low temperature | 4.999 | 1.1800E-28 |  | C2H2 | ZOS3-21 - C2H2 zinc finger protein, expressed | Salinity tolerance. | | LOC_Os01g12690 | Low temperature | 4.858 | 1.9300E-14 |  | OFP | plant-specific domain TIGR01568 family protein, expressed |  | | LOC_Os09g35010 | Low temperature | 4.79 | 1.4200E-36 | RSV | AP2-EREBP | dehydration-responsive element-binding protein, putative, expressed | Cold, drought and salinity tolerance. | | LOC_Os05g25770 | Low temperature | 4.744 | 6.3200E-22 |  | WRKY | WRKY45, expressed | Cold, drought and salinity tolerance. ABA sensitivity. | | LOC_Os04g43680 | Low temperature | 4.607 | 2.5400E-28 | M. oryzae | MYB | MYB family transcription factor, putative, expressed |  | | LOC_Os03g20090 | Low temperature | 4.596 | 9.4400E-38 |  | MYB | MYB family transcription factor, putative, expressed | Cold, drought and salinity tolerance. ABA sensitivity. | | LOC_Os02g41510 | Low temperature | 4.578 | 2.2100E-26 | M. oryzae | MYB | MYB family transcription factor, putative, expressed |  | | LOC_Os02g08440 | Low temperature | 4.535 | 4.2300E-32 | RSV | WRKY | WRKY71, expressed | Resistance to Xanthomonas oryzae pv. oryzae. | | LOC_Os03g08310 | Low temperature | 4.418 | 7.5300E-22 | RSV | Tify | ZIM domain containing protein, putative, expressed | Drought and salinity tolerance. | | LOC_Os10g25230 | Low temperature | 4.346 | 3.1700E-23 | RSV | Tify | ZIM domain containing protein, putative, expressed |  | | LOC_Os01g21120 | Low temperature | 4.018 | 3.4800E-22 |  | AP2-EREBP | AP2 domain containing protein, expressed |  | | LOC_Os01g73770 | Low temperature | 3.925 | 5.7800E-10 | RSV | AP2-EREBP | dehydration-responsive element-binding protein, putative, expressed | Cold, drought and salinity tolerance. | | LOC_Os02g32590 | Low temperature | 3.87 | 7.4100E-20 |  | HSF | HSF-type DNA-binding domain containing protein, expressed |  | | LOC_Os07g47790 | Low temperature | 3.787 | 5.2300E-23 |  | AP2-EREBP | AP2 domain containing protein, expressed |  | | LOC_Os03g08330 | Low temperature | 3.759 | 1.0400E-26 | RSV | Tify | ZIM domain containing protein, putative, expressed | Grain size. Plant height | | LOC_Os02g09480 | Low temperature | 3.752 | 1.2400E-14 |  | MYB | myb-like DNA-binding domain containing protein, putative, expressed | | | LOC_Os01g64310 | Low temperature | 3.688 | 7.2000E-18 |  | NAC | no apical meristem protein, putative, expressed |  | | LOC_Os02g46030 | Low temperature | 3.612 | 5.5500E-21 | M. grisea | MYB-related | MYB family transcription factor, putative, expressed |  | | LOC_Os09g35030 | Low temperature | 3.604 | 3.5300E-22 |  | AP2-EREBP | dehydration-responsive element-binding protein, putative, expressed | Cold, drought and salinity tolerance. | | LOC_Os03g15660 | Low temperature | 3.573 | 5.8900E-15 |  | AP2-EREBP | AP2 domain containing protein, expressed |  | | LOC_Os03g53020 | Low temperature | 3.513 | 7.8300E-18 | RSV | bHLH | helix-loop-helix DNA-binding domain containing protein, expressed | Drought tolerance. | | LOC_Os01g61080 | Low temperature | 3.513 | 9.1200E-22 | M. oryzae | WRKY | WRKY24, expressed |  | | LOC_Os01g64360 | Low temperature | 3.408 | 8.8700E-22 |  | MYB | MYB family transcription factor, putative, expressed |  | | LOC_Os05g07010 | Low temperature | 3.408 | 2.0200E-15 | RSV | MYB-related | myb-like DNA-binding domain containing protein, expressed |  | | LOC_Os01g51690 | Low temperature | 3.315 | 6.3400E-12 | M. oryzae | WRKY | WRKY26, expressed |  | | LOC_Os07g39220 | Low temperature | 3.258 | 1.0300E-22 |  | BES1 | BES1/BZR1 homolog protein, putative, expressed | Dwarfism. Leaf angle. Brassinosteroid sensitivity. | | LOC_Os01g60600 | Low temperature | 3.206 | 5.0100E-17 |  | WRKY | WRKY108, expressed |  | | LOC_Os01g53220 | Low temperature | 3.198 | 2.1000E-13 | BPH | HSF | HSF-type DNA-binding domain containing protein, expressed |  | | LOC_Os02g22020 | Low temperature | 3.192 | 1.3900E-16 |  | G2-like | MYB family transcription factor, putative, expressed |  | | LOC_Os05g37190 | Low temperature | 3.184 | 4.2400E-18 | BPH | C2H2 | ZOS5-08 - C2H2 zinc finger protein, expressed |  | | LOC_Os10g25290 | Low temperature | 3.159 | 1.7900E-19 |  | Tify | ZIM domain containing protein, putative, expressed |  | | LOC_Os02g40530 | Low temperature | 3.155 | 1.0500E-09 |  | MYB | MYB family transcription factor, putative, expressed | Grain size. Total biomass. | | LOC_Os03g32230 | Low temperature | 3.142 | 1.5400E-19 | M. oryzae | C2H2 | ZOS3-12 - C2H2 zinc finger protein, expressed |  | | LOC_Os07g22730 | Low temperature | 3.134 | 4.3700E-15 |  | AP2-EREBP | AP2 domain containing protein, expressed |  | | LOC_Os05g46020 | Low temperature | 3.132 | 4.1600E-30 | M. oryzae | WRKY | WRKY7, expressed |  | | LOC_Os01g50940 | Low temperature | 3.122 | 8.1700E-26 | RSV | bHLH | helix-loop-helix DNA-binding domain containing protein, expressed |  | | LOC_Os03g08320 | Low temperature | 3.09 | 2.1600E-17 | M. oryzae | Tify | ZIM domain containing protein, putative, expressed |  | | LOC_Os01g19330 | Low temperature | 3.082 | 9.9700E-13 |  | MYB | MYB family transcription factor, putative, expressed |  | | LOC_Os07g12340 | Low temperature | 3.05 | 1.8600E-33 |  | NAC | NAC domain-containing protein 67, putative, expressed |  | | LOC_Os02g45420 | Low temperature | 3.026 | 4.2000E-13 |  | AP2-EREBP | AP2 domain containing protein, expressed |  | | LOC_Os10g33810 | Low temperature | 3.019 | 2.1600E-12 |  | MYB | myb-related protein Myb4, putative, expressed |  | | LOC_Os05g39720 | Low temperature | 3.016 | 2.6500E-13 | M. oryzae | WRKY | WRKY70, expressed |  | | LOC_Os09g31438 | Low temperature | 3.013 | 1.3600E-10 |  | SBP | OsSPL17 - SBP-box gene family member, expressed |  | | LOC_Os08g06659 | Low temperature | 2.951 | 2.5800E-08 |  | LOB | expressed protein |  | | LOC_Os05g50700 | Low temperature | 2.95 | 4.7200E-11 |  | WRKY | WRKY111, expressed |  | | LOC_Os01g74020 | Low temperature | 2.914 | 3.7500E-05 | BPH | G2-like | MYB family transcription factor, putative, expressed |  | | LOC_Os03g09170 | Low temperature | 2.872 | 1.9000E-10 |  | AP2-EREBP | ethylene-responsive transcription factor, putative, expressed |  | | LOC_Os03g58350 | Low temperature | 2.821 | 3.2500E-05 |  | AUX_IAA | OsIAA14 - Auxin-responsive Aux/IAA gene family member, expressed | | | LOC_Os01g15900 | Low temperature | 2.809 | 2.0700E-17 | XOO | C2C2-Dof | expressed protein | Grain length and width. 1000-grain weight. Flowering time. | | LOC_Os06g43090 | Low temperature | 2.803 | 1.7200E-26 |  | MYB | MYB transcription factor TaMYB1, putative, expressed |  | | LOC_Os02g43170 | Low temperature | 2.787 | 9.6800E-20 |  | Orphans | B-box zinc finger family protein, putative, expressed |  | | LOC_Os02g53690 | Low temperature | 2.779 | 7.0900E-08 |  | GRF | growth regulating factor protein, putative, expressed |  | | LOC_Os06g04090 | Low temperature | 2.774 | 2.0400E-09 |  | NAC | no apical meristem protein, putative, expressed |  | | LOC_Os01g58420 | Low temperature | 2.771 | 4.8400E-37 |  | AP2-EREBP | AP2 domain containing protein, expressed | Drought tolerance through controlling the ethylene biosynthesis. | | LOC_Os05g35500 | Low temperature | 2.769 | 7.7100E-21 |  | MYB | MYB family transcription factor, putative, expressed |  | | LOC_Os01g40260 | Low temperature | 2.764 | 3.0300E-11 | M. grisea | WRKY | WRKY77, expressed |  | | LOC_Os03g12370 | Low temperature | 2.746 | 8.5800E-15 |  | HSF | HSF-type DNA-binding domain containing protein, expressed |  | | LOC_Os02g56250 | Low temperature | 2.745 | 1.0500E-12 |  | C2C2-GATA | GATA zinc finger domain containing protein, expressed |  | | LOC_Os09g25060 | Low temperature | 2.725 | 5.0000E-10 | RSV | WRKY | WRKY76, expressed | Resistance to Xanthomonas oryzae pv. oryzae. | | LOC_Os01g14870 | Low temperature | 2.704 | 2.1400E-14 |  | C3H | zinc finger matrin-type protein 5, putative, expressed |  | | LOC_Os01g12440 | Low temperature | 2.67 | 3.0300E-08 |  | AP2-EREBP | AP2 domain containing protein, expressed |  | | LOC_Os01g60640 | Low temperature | 2.663 | 9.2300E-20 | RSV | WRKY | WRKY21, expressed |  | | LOC_Os01g60020 | Low temperature | 2.659 | 9.0200E-14 | M. oryzae | NAC | NAC domain transcription factor, putative, expressed | Bacterial blight resistance. HR cell death. | | LOC_Os05g46370 | Low temperature | 2.659 | 4.3400E-06 | BPH | bHLH | bHelix-loop-helix transcription factor, putative, expressed |  | | LOC_Os02g43790 | Low temperature | 2.649 | 4.8100E-18 | M. oryzae | AP2-EREBP | ethylene-responsive transcription factor, putative, expressed |  | | LOC_Os11g12650 | Low temperature | 2.587 | 1.6000E-05 |  | PHD | PHD-finger domain containing protein, putative, expressed |  | | LOC_Os05g41760 | Low temperature | 2.578 | 9.3900E-17 |  | AP2-EREBP | AP2 domain containing protein, expressed | Spikelet determinacy. Floral organ development. | | LOC_Os02g26430 | Low temperature | 2.527 | 4.4600E-09 |  | WRKY | WRKY42, expressed | promotion of leaf senescence through ROS accumulation, plant death | | LOC_Os01g50110 | Low temperature | 2.526 | 4.6100E-06 | M. grisea | MYB | MYB family transcription factor, putative, expressed |  | | LOC_Os06g06900 | Low temperature | 2.515 | 1.7900E-11 |  | bHLH | helix-loop-helix DNA-binding domain containing protein, expressed |  | | LOC_Os04g23550 | Low temperature | 2.458 | 3.0500E-08 | M. oryzae | bHLH | basic helix-loop-helix family protein, putative, expressed | Dwarfism. JA sensitivity during seedling stage. | | LOC_Os03g21030 | Low temperature | 2.444 | 8.1700E-18 |  | NAC | no apical meristem protein, putative, expressed |  | | LOC_Os03g21660 | Low temperature | 2.413 | 5.8800E-05 |  | FAR1 | transposon protein, putative, unclassified, expressed |  | | LOC_Os04g08740 | Low temperature | 2.401 | 3.0200E-07 |  | Orphans | ethylene receptor, putative, expressed | Flowering time. Ethylene sensitivity. Stem starch content. | | LOC_Os02g55560 | Low temperature | 2.357 | 4.6700E-10 |  | DBP | protein phosphatase 2C, putative, expressed |  | | LOC_Os03g21710 | Low temperature | 2.313 | 3.9900E-08 | BPH | WRKY | WRKY79, expressed |  | | LOC_Os02g43970 | Low temperature | 2.266 | 1.5000E-06 |  | AP2-EREBP | AP2 domain containing protein, expressed | Drought tolerance during seedling stage. ABA sensitivity during germination. | | LOC_Os07g07974 | Low temperature | 2.263 | 1.1900E-14 |  | CPP | tesmin/TSO1-like CXC domain containing protein, expressed |  | | LOC_Os03g56630 | Low temperature | 2.248 | 5.3700E-17 |  | FAR1 | transposon protein, putative, unclassified, expressed |  | | LOC_Os01g65370 | Low temperature | 2.24 | 2.1400E-12 |  | MYB | MYB family transcription factor, putative, expressed |  | | LOC_Os09g28210 | Low temperature | 2.236 | 1.6900E-10 |  | bHLH | bHelix-loop-helix transcription factor, putative, expressed |  | | LOC_Os01g14440 | Low temperature | 2.229 | 1.1300E-13 |  | WRKY | WRKY1, expressed |  | | LOC_Os05g41780 | Low temperature | 2.214 | 3.5200E-27 |  | AP2-EREBP | AP2 domain containing protein, expressed |  | | LOC_Os04g52090 | Low temperature | 2.211 | 1.0400E-25 |  | AP2-EREBP | AP2 domain containing protein, expressed | Dwarfism. Fertility. Drought tolerance. | | LOC_Os10g38820 | Low temperature | 2.195 | 4.5200E-08 | XOO | bZIP | bZIP family transcription factor, putative, expressed |  | | LOC_Os06g45140 | Low temperature | 2.171 | 1.2900E-10 |  | bZIP | bZIP transcription factor domain containing protein, expressed | Cold and drought tolerance. | | LOC_Os04g51190 | Low temperature | 2.167 | 5.1800E-06 |  | GRF | growth-regulating factor, putative, expressed |  | | LOC_Os02g51280 | Low temperature | 2.16 | 2.8800E-06 |  | TCP | TCP-domain protein, putative, expressed |  | | LOC_Os01g19130 | Low temperature | 2.149 | 2.8000E-15 |  | DBP | protein phosphatase 2C, putative, expressed |  | | LOC_Os02g40510 | Low temperature | 2.128 | 9.2800E-08 |  | Pseudo_ARR-B | response regulator receiver domain containing protein, expressed |  | | LOC_Os01g63980 | Low temperature | 2.115 | 1.2000E-05 |  | C2H2 | ZOS1-17 - C2H2 zinc finger protein, expressed |  | | LOC_Os01g39330 | Low temperature | 2.083 | 6.2100E-10 | M. oryzae | bHLH | helix-loop-helix DNA-binding domain containing protein, expressed |  | | LOC_Os07g36170 | Low temperature | 2.077 | 7.0000E-09 |  | GRAS | chitin-inducible gibberellin-responsive protein, putative, expressed |  | | LOC_Os02g01860 | Low temperature | 2.064 | 2.9500E-06 |  | FAR1 | transposon protein, putative, unclassified |  | | LOC_Os03g19020 | Low temperature | 2.062 | 1.6200E-13 |  | PHD | PHD-finger family protein, expressed |  | | LOC_Os11g47630 | Low temperature | 2.045 | 1.0655E-04 | M. grisea | C2H2 | ZOS11-10 - C2H2 zinc finger protein, expressed |  | | LOC_Os04g48350 | Low temperature | 2.043 | 2.7000E-10 | RSV | AP2-EREBP | dehydration-responsive element-binding protein, putative, expressed | Drought tolerance. | | LOC_Os10g39540 | Low temperature | 2.042 | 1.2700E-15 |  | DBP | protein phosphatase 2C, putative, expressed |  | | LOC_Os05g50610 | Low temperature | 2.023 | 2.8500E-05 |  | WRKY | WRKY8, expressed |  | | LOC_Os03g14850 | Low temperature | 2.018 | 4.2500E-09 | BPH | MADS | OsMADS72 - MADS-box family gene with M-alpha type-box, expressed | | | LOC_Os04g43910 | Low temperature | 2.014 | 9.7100E-07 |  | ARF | auxin response factor, putative, expressed |  | | LOC_Os05g27730 | Low temperature | 2.01 | 8.3100E-12 |  | WRKY | WRKY53, expressed | Resistance to Magnaporthe grisea. | | LOC_Os05g34730 | Low temperature | 2.001 | 2.0663E-03 |  | AP2-EREBP | ethylene-responsive transcription factor ERF020, putative, expressed | Salinity tolerance. | | LOC_Os01g68370 | Submergance | 5.1 | 5.1800E-14 |  | ABI3VP1 | B3 DNA binding domain containing protein, expressed |  | | LOC_Os04g28120 | Submergance | 4.787 | 1.2600E-09 |  | Orphans | response regulator receiver domain containing protein, expressed |  | | LOC_Os07g47790 | Submergance | 4.038 | 1.7300E-07 |  | AP2-EREBP | AP2 domain containing protein, expressed |  | | LOC_Os06g44750 | Submergance | 3.83 | 2.1100E-10 |  | AP2-EREBP | AP2 domain containing protein, expressed |  | | LOC_Os07g15540 | Submergance | 3.827 | 1.0097E-03 |  | Orphans | ethylene receptor, putative, expressed |  | | LOC_Os04g55970 | Submergance | 3.523 | 3.3000E-10 |  | AP2-EREBP | AP2-like ethylene-responsive transcription factor AINTEGUMENTA, putative, expressed | | | LOC_Os03g20910 | Submergance | 3.499 | 1.7500E-10 | XOO | HB | homeobox domain containing protein, expressed |  | | LOC_Os11g05640 | Submergance | 3.417 | 5.7800E-12 |  | bZIP | bZIP transcription factor domain containing protein, expressed |  | | LOC_Os03g50920 | Submergance | 3.338 | 3.3600E-09 |  | zf-HD | ZF-HD protein dimerisation region containing protein, expressed |  | | LOC_Os03g07940 | Submergance | 3.029 | 9.0400E-09 |  | AP2-EREBP | AP2 domain containing protein, expressed |  | | LOC_Os05g29810 | Submergance | 3.013 | 2.5600E-07 |  | AP2-EREBP | AP2 domain containing protein, expressed |  | | LOC_Os03g51690 | Submergance | 2.992 | 2.8600E-15 |  | HB | Homeobox domain containing protein, expressed | Knotted leaf. | | LOC_Os05g28350 | Submergance | 2.991 | 6.1600E-12 |  | AP2-EREBP | AP2 domain containing protein, expressed |  | | LOC_Os07g03770 | Submergance | 2.944 | 8.2500E-07 |  | HB | Homeobox domain containing protein, expressed | Shoot organization. Leaf morphology. | | LOC_Os03g19900 | Submergance | 2.913 | 2.9000E-08 |  | AP2-EREBP | AP2 domain containing protein, expressed |  | | LOC_Os01g46870 | Submergance | 2.884 | 3.8300E-15 |  | AP2-EREBP | AP2 domain containing protein, expressed |  | | LOC_Os02g42950 | Submergance | 2.88 | 3.1400E-12 |  | C2C2-YABBY | YABBY domain containing protein, putative, expressed |  | | LOC_Os01g64000 | Submergance | 2.865 | 1.9036E-04 |  | bZIP | bZIP transcription factor, putative, expressed | Salinity tolerance. Fertility. | | LOC_Os02g48060 | Submergance | 2.852 | 3.7700E-05 |  | bHLH | helix-loop-helix DNA-binding domain containing protein, expressed |  | | LOC_Os02g40070 | Submergance | 2.672 | 2.7800E-05 |  | AP2-EREBP | AP2-like ethylene-responsive transcription factor PLETHORA 2, putative, expressed | | | LOC_Os04g52770 | Submergance | 2.659 | 3.6800E-09 |  | bHLH | helix-loop-helix DNA-binding domain containing protein, expressed |  | | LOC_Os03g51970 | Submergance | 2.578 | 1.0800E-10 |  | GRF | growth-regulating factor, putative, expressed | open husk, long sterile lemma | | LOC_Os08g43210 | Submergance | 2.576 | 1.4504E-03 |  | AP2-EREBP | AP2 domain containing protein, expressed |  | | LOC_Os01g34610 | Submergance | 2.514 | 1.3200E-10 |  | Orphans | expressed protein |  | | LOC_Os04g45330 | Submergance | 2.489 | 1.0782E-04 |  | C2C2-YABBY | YABBY domain containing protein, putative, expressed | Ligule and auricle formation. Leaf morphology. | | LOC_Os08g43200 | Submergance | 2.455 | 8.1629E-03 |  | AP2-EREBP | dehydration-responsive element-binding protein, putative, expressed | Drought tolerance. | | LOC_Os03g21160 | Submergance | 2.396 | 3.7514E-03 |  | C3H | RNA-binding zinc finger protein, putative, expressed |  | | LOC_Os06g39240 | Submergance | 2.378 | 8.7800E-05 | RSV | MBF1 | endothelial differentiation-related factor 1, putative, expressed |  | | LOC_Os07g48870 | Submergance | 2.377 | 7.8600E-06 |  | MYB | MYB family transcription factor, putative, expressed |  | | LOC_Os01g09620 | Submergance | 2.33 | 1.9600E-08 |  | C3H | zinc finger/CCCH transcription factor, putative, expressed | Leaf senescence. JA sensitivity. | | LOC_Os11g19060 | Submergance | 2.309 | 1.7200E-06 |  | AP2-EREBP | BABY BOOM 1, putative, expressed |  | | LOC_Os06g44410 | Submergance | 2.28 | 1.7100E-05 |  | Orphans | histidine kinase, putative, expressed |  | | LOC_Os02g46030 | Submergance | 2.218 | 1.4882E-02 | M. grisea | MYB-related | MYB family transcription factor, putative, expressed |  | | LOC_Os01g47710 | Submergance | 2.198 | 4.6900E-12 |  | HB | homeobox domain containing protein, expressed | internode elongation, cell division and elongation, tiller growth | | LOC_Os02g34260 | Submergance | 2.192 | 1.1634E-02 |  | AP2-EREBP | AP2 domain containing protein, expressed |  | | LOC_Os01g19694 | Submergance | 2.183 | 1.0100E-07 |  | HB | Homeobox domain containing protein, expressed | Bract differentiation especially at the basal nodes of panicles. | | LOC_Os08g01700 | Submergance | 2.177 | 1.4000E-09 | BPH | bHLH | basic helix-loop-helix family protein, putative, expressed |  | | LOC_Os01g32770 | Submergance | 2.167 | 8.4072E-04 |  | LOB | DUF260 domain containing protein, putative, expressed |  | | LOC_Os04g46250 | Submergance | 2.166 | 6.6795E-03 |  | AP2-EREBP | AP2 domain containing protein, expressed |  | | LOC_Os02g53690 | Submergance | 2.134 | 3.5264E-04 |  | GRF | growth regulating factor protein, putative, expressed |  | | LOC_Os08g14400 | Submergance | 2.127 | 6.6498E-04 |  | HB | homeobox domain containing protein, expressed |  | | LOC_Os01g21120 | Submergance | 2.109 | 8.0727E-04 |  | AP2-EREBP | AP2 domain containing protein, expressed |  | | LOC_Os02g15350 | Submergance | 2.108 | 1.2600E-05 |  | C2C2-Dof | dof zinc finger domain containing protein, putative, expressed | Seed storage protein and starch content. | | LOC_Os03g50780 | Submergance | 2.106 | 2.0241E-03 |  | PHD | PHD-finger domain containing protein, putative, expressed |  | | LOC_Os02g43170 | Submergance | 2.102 | 5.9100E-05 |  | Orphans | B-box zinc finger family protein, putative, expressed |  | | LOC_Os01g02110 | Submergance | 2.099 | 1.3043E-03 |  | bHLH | helix-loop-helix DNA-binding domain containing protein, expressed |  | | LOC_Os08g42600 | Submergance | 2.083 | 1.9700E-08 |  | RB | retinoblastoma-related protein-like, putative, expressed |  | | LOC_Os06g06900 | Submergance | 2.041 | 1.6800E-06 |  | bHLH | helix-loop-helix DNA-binding domain containing protein, expressed |  | | LOC_Os01g62660 | Submergance | 2.001 | 1.2888E-04 |  | G2-like | MYB family transcription factor, putative, expressed |  | |

**Table S7**. Information on biotic stress-induced transcription factor and transcription regulator genes.

| **Locus_ID Pathogen stress Fold change p-value Abiotic stress response Family RGAP V7 Annotation** |
| --- |
| | LOC_Os11g47630 | M. grisea | 2.766 | | 0.0003 | Low temperature | C2H2 | ZOS11-10 - C2H2 zinc finger protein, expressed | | --- | --- | --- | --- | --- | --- | --- | --- | | LOC_Os01g50110 | M. grisea | | 2.403 | 0.0006 | Low temperature | MYB | MYB family transcription factor, putative, expressed | | LOC_Os01g18360 | M. grisea | | 2.365 | 0.0106 |  | AUX_IAA | OsIAA4 - Auxin-responsive Aux/IAA gene family member, expressed | | LOC_Os02g46030 | M. grisea | | 2.295 | 0.0206 | Drought | MYB-related | MYB family transcription factor, putative, expressed | | LOC_Os04g58190 | M. grisea | | 2.171 | 0.0004 |  | C2C2-Dof | dof zinc finger domain containing protein, putative, expressed | | LOC_Os01g40260 | M. grisea | | 1.981 | 0.0381 | Low temperature | WRKY | WRKY77, expressed | | LOC_Os01g09550 | M. grisea | | 1.977 | 0.024 |  | NAC | no apical meristem protein, putative, expressed | | LOC_Os01g09990 | M. grisea | | 1.753 | 0.0445 |  | bHLH | helix-loop-helix DNA-binding domain containing protein, expressed | | LOC_Os07g37210 | M. grisea | | 1.752 | 0.0145 | Drought | MYB | MYB family transcription factor, putative, expressed | | LOC_Os02g57790 | M. grisea | | 1.652 | 0.0414 |  | C2H2 | ZOS2-19 - C2H2 zinc finger protein, expressed | | LOC_Os01g11910 | M. grisea | | 1.557 | 0.0215 |  | bHLH | basic helix-loop-helix, putative, expressed | | LOC_Os11g05740 | M. grisea | | 1.448 | 0.0482 |  | ABI3VP1 | B3 DNA binding domain containing protein, expressed | | LOC_Os12g01490 | M. grisea | | 1.384 | 0.0104 |  | G2-like | MYB family transcription factor, putative, expressed | | LOC_Os11g01480 | M. grisea | | 1.382 | 0.0188 |  | G2-like | MYB family transcription factor, putative, expressed | | LOC_Os06g44010 | M. oryzae | | 2.91 | 0 |  | WRKY | WRKY28, expressed | | LOC_Os01g39330 | M. oryzae | | 2.795 | 0.0001 | Low temperature | bHLH | helix-loop-helix DNA-binding domain containing protein, expressed | | LOC_Os04g58190 | M. oryzae | | 2.819 | 0.0122 |  | C2C2-Dof | dof zinc finger domain containing protein, putative, expressed | | LOC_Os02g41510 | M. oryzae | | 2.712 | 0.0003 | Low temperature | MYB | MYB family transcription factor, putative, expressed | | LOC_Os02g12820 | M. oryzae | | 2.707 | 0.0164 |  | bHLH | helix-loop-helix DNA-binding domain containing protein, expressed | | LOC_Os07g22770 | M. oryzae | | 2.606 | 0.0132 |  | AP2-EREBP | AP2 domain containing protein, expressed | | LOC_Os04g32480 | M. oryzae | | 2.542 | 0.0068 |  | Tify | zinc-finger protein, putative, expressed | | LOC_Os09g23620 | M. oryzae | | 2.506 | 0.0078 |  | MYB | MYB family transcription factor, putative, expressed | | LOC_Os01g71850 | M. oryzae | | 2.386 | 0.0336 |  | FAR1 | transposon protein, putative, unclassified, expressed | | LOC_Os01g40260 | M. oryzae | | 2.256 | 0.0269 | Low temperature | WRKY | WRKY77, expressed | | LOC_Os03g32230 | M. oryzae | | 2.251 | 0.0031 | Low temperature | C2H2 | ZOS3-12 - C2H2 zinc finger protein, expressed | | LOC_Os05g46020 | M. oryzae | | 2.095 | 0.0002 | Low temperature | WRKY | WRKY7, expressed | | LOC_Os01g61080 | M. oryzae | | 1.964 | 0.0002 | Low temperature | WRKY | WRKY24, expressed | | LOC_Os04g23550 | M. oryzae | | 1.873 | 0.034 | Low temperature | bHLH | basic helix-loop-helix family protein, putative, expressed | | LOC_Os05g39720 | M. oryzae | | 1.843 | 0.0077 | Low temperature | WRKY | WRKY70, expressed | | LOC_Os07g27670 | M. oryzae | | 1.75 | 0.0203 |  | WRKY | WRKY115, expressed | | LOC_Os11g01480 | M. oryzae | | 1.738 | 0.0076 |  | G2-like | MYB family transcription factor, putative, expressed | | LOC_Os01g51690 | M. oryzae | | 1.72 | 0.0086 | Low temperature | WRKY | WRKY26, expressed | | LOC_Os04g52810 | M. oryzae | | 1.632 | 0.024 |  | NAC | no apical meristem protein, putative, expressed | | LOC_Os04g43680 | M. oryzae | | 1.596 | 0.0032 | Low temperature | MYB | MYB family transcription factor, putative, expressed | | LOC_Os01g60020 | M. oryzae | | 1.557 | 0.0104 | Low temperature | NAC | NAC domain transcription factor, putative, expressed | | LOC_Os06g06750 | M. oryzae | | 1.556 | 0.0299 |  | MADS | OsMADS5 - MADS-box family gene with MIKCc type-box, expressed | | LOC_Os01g46800 | M. oryzae | | 1.537 | 0.0399 |  | WRKY | WRKY15, expressed | | LOC_Os03g08320 | M. oryzae | | 1.512 | 0.0358 | Low temperature | Tify | ZIM domain containing protein, putative, expressed | | LOC_Os12g01490 | M. oryzae | | 1.511 | 0.0101 |  | G2-like | MYB family transcription factor, putative, expressed | | LOC_Os03g32220 | M. oryzae | | 1.467 | 0.0495 |  | C2H2 | ZOS3-11 - C2H2 zinc finger protein, expressed | | LOC_Os07g48260 | M. oryzae | | 1.371 | 0.0104 |  | WRKY | WRKY47, expressed | | LOC_Os02g43790 | M. oryzae | | 1.268 | 0.0289 | Low temperature | AP2-EREBP | ethylene-responsive transcription factor, putative, expressed | | LOC_Os09g16510 | M. oryzae | | 1.163 | 0.0465 |  | WRKY | WRKY74, expressed | | LOC_Os03g08330 | RSV | | 2.59 | 0.0001 | Low temperature | Tify | ZIM domain containing protein, putative, expressed | | LOC_Os09g28440 | RSV | | 4.511 | 0.0023 | Low temperature | AP2-EREBP | AP2 domain containing protein, expressed | | LOC_Os02g54050 | RSV | | 3.555 | 0.0005 |  | AP2-EREBP | ethylene-responsive transcription factor, putative, expressed | | LOC_Os05g36970 | RSV | | 3.494 | 0.0172 |  | OFP | DUF623 domain containing protein, expressed | | LOC_Os09g35010 | RSV | | 3.419 | 0.0145 | Low temperature | AP2-EREBP | dehydration-responsive element-binding protein, putative, expressed | | LOC_Os08g43334 | RSV | | 3.37 | 0.0038 |  | HSF | HSF-type DNA-binding domain containing protein, expressed | | LOC_Os03g21870 | RSV | | 3.299 | 0.0067 |  | OFP | DUF623 domain containing protein, expressed | | LOC_Os01g53260 | RSV | | 3.091 | 0.039 |  | WRKY | WRKY23, expressed | | LOC_Os05g07010 | RSV | | 3.006 | 0.0045 | Low temperature | MYB-related | myb-like DNA-binding domain containing protein, expressed | | LOC_Os12g05990 | RSV | | 2.956 | 0.0175 |  | NAC | No apical meristem protein, putative, expressed | | LOC_Os03g08310 | RSV | | 2.935 | 0.0001 | Low temperature | Tify | ZIM domain containing protein, putative, expressed | | LOC_Os09g25060 | RSV | | 2.787 | 0.016 | Low temperature | WRKY | WRKY76, expressed | | LOC_Os06g39240 | RSV | | 2.76 | 0.0092 | Submergance | MBF1 | endothelial differentiation-related factor 1, putative, expressed | | LOC_Os06g03670 | RSV | | 2.7 | 0.0066 | Drought | AP2-EREBP | dehydration-responsive element-binding protein, putative, expressed | | LOC_Os01g73770 | RSV | | 2.691 | 0.0064 | Low temperature | AP2-EREBP | dehydration-responsive element-binding protein, putative, expressed | | LOC_Os02g08440 | RSV | | 2.655 | 0.0302 | Low temperature | WRKY | WRKY71, expressed | | LOC_Os08g36920 | RSV | | 2.552 | 0.0009 | Drought | AP2-EREBP | AP2 domain containing protein, expressed | | LOC_Os10g25230 | RSV | | 2.457 | 0.0003 | Low temperature | Tify | ZIM domain containing protein, putative, expressed | | LOC_Os07g32170 | RSV | | 2.443 | 0.0487 |  | SBP | OsSPL13 - SBP-box gene family member, expressed | | LOC_Os02g45450 | RSV | | 2.382 | 0.0184 | Low temperature | AP2-EREBP | dehydration-responsive element-binding protein, putative, expressed | | LOC_Os01g09990 | RSV | | 2.38 | 0.0342 |  | bHLH | helix-loop-helix DNA-binding domain containing protein, expressed | | LOC_Os07g36460 | RSV | | 2.271 | 0.0026 |  | bHLH | helix-loop-helix DNA-binding domain containing protein, expressed | | LOC_Os01g60640 | RSV | | 2.264 | 0.0065 | Low temperature | WRKY | WRKY21, expressed | | LOC_Os04g48030 | RSV | | 2.253 | 0.0001 |  | HSF | heat stress transcription factor B-1, putative, expressed | | LOC_Os11g47630 | RSV | | 2.217 | 0.0099 | Low temperature | C2H2 | ZOS11-10 - C2H2 zinc finger protein, expressed | | LOC_Os03g31880 | RSV | | 2.206 | 0.0016 |  | GRAS | SHORT-ROOT, putative, expressed | | LOC_Os08g29660 | RSV | | 2.192 | 0.0356 |  | WRKY | WRKY69, expressed | | LOC_Os09g25070 | RSV | | 2.027 | 0.0442 |  | WRKY | WRKY62, expressed | | LOC_Os05g37080 | RSV | | 1.993 | 0.0003 |  | NAC | No apical meristem protein, putative, expressed | | LOC_Os11g05614 | RSV | | 1.952 | 0.0161 |  | NAC | no apical meristem protein, putative, expressed | | LOC_Os01g50940 | RSV | | 1.798 | 0.0365 | Low temperature | bHLH | helix-loop-helix DNA-binding domain containing protein, expressed | | LOC_Os04g48350 | RSV | | 1.793 | 0.0435 | Low temperature | AP2-EREBP | dehydration-responsive element-binding protein, putative, expressed | | LOC_Os05g49620 | RSV | | 1.791 | 0.0244 |  | WRKY | WRKY19, expressed | | LOC_Os01g70870 | RSV | | 1.741 | 0.0355 |  | C2H2 | ZOS1-23 - C2H2 zinc finger protein, expressed | | LOC_Os02g06330 | RSV | | 1.696 | 0.0328 |  | AP2-EREBP | AP2 domain containing protein, expressed | | LOC_Os06g14670 | RSV | | 1.691 | 0.023 | Drought | MYB | ODORANT1, putative, expressed | | LOC_Os09g35790 | RSV | | 1.645 | 0.0393 |  | HSF | HSF-type DNA-binding domain containing protein, expressed | | LOC_Os02g49250 | RSV | | 1.639 | 0.0466 |  | MYB | myb-like DNA-binding domain containing protein, expressed | | LOC_Os03g53020 | RSV | | 1.612 | 0.0195 | Low temperature | bHLH | helix-loop-helix DNA-binding domain containing protein, expressed | | LOC_Os03g53340 | RSV | | 1.609 | 0.0329 | Drought | HSF | HSF-type DNA-binding domain containing protein, expressed | | LOC_Os05g50930 | RSV | | 1.247 | 0.0103 |  | Orphans | RNA polymerase sigma factor, putative, expressed | | LOC_Os02g43330 | XOO | | 3.465 | 0.0002 | Drought | HB | homeobox associated leucine zipper, putative, expressed | | LOC_Os09g29820 | XOO | | 3.35 | 0.0024 |  | bZIP | bZIP transcription factor domain containing protein, expressed | | LOC_Os05g38620 | XOO | | 3.272 | 0.0003 |  | C2H2 | ZOS5-10 - C2H2 zinc finger protein, expressed | | LOC_Os11g45740 | XOO | | 2.851 | 0 | Drought | MYB | MYB family transcription factor, putative, expressed | | LOC_Os01g15900 | XOO | | 2.536 | 0.0007 | Low temperature | C2C2-Dof | expressed protein | | LOC_Os08g41030 | XOO | | 2.377 | 0.0223 |  | AP2-EREBP | AP2 domain containing protein, expressed | | LOC_Os10g38820 | XOO | | 2.343 | 0.0014 | Drought | bZIP | bZIP family transcription factor, putative, expressed | | LOC_Os04g58190 | XOO | | 2.312 | 0 |  | C2C2-Dof | dof zinc finger domain containing protein, putative, expressed | | LOC_Os11g47630 | XOO | | 2.095 | 0.0122 | Low temperature | C2H2 | ZOS11-10 - C2H2 zinc finger protein, expressed | | LOC_Os01g50940 | XOO | | 2.091 | 0.0003 | Low temperature | bHLH | helix-loop-helix DNA-binding domain containing protein, expressed | | LOC_Os03g20910 | XOO | | 2.089 | 0.008 | Submergance | HB | homeobox domain containing protein, expressed | | LOC_Os04g32480 | XOO | | 2.016 | 0.0059 |  | Tify | zinc-finger protein, putative, expressed | | LOC_Os01g09990 | XOO | | 1.884 | 0.029 |  | bHLH | helix-loop-helix DNA-binding domain containing protein, expressed | | LOC_Os04g43680 | XOO | | 1.832 | 0 | Low temperature | MYB | MYB family transcription factor, putative, expressed | | LOC_Os12g05990 | XOO | | 1.782 | 0.0002 |  | NAC | No apical meristem protein, putative, expressed | | LOC_Os03g17810 | XOO | | 1.748 | 0.0173 |  | LOB | DUF260 domain containing protein, putative, expressed | | LOC_Os03g19370 | XOO | | 1.659 | 0.0019 |  | bZIP | CPuORF4 - conserved peptide uORF-containing transcript, expressed | | LOC_Os05g49620 | XOO | | 1.643 | 0.0485 |  | WRKY | WRKY19, expressed | | LOC_Os10g35300 | XOO | | 1.632 | 0.0282 |  | C2C2-Dof | dof zinc finger domain containing protein, putative, expressed | | LOC_Os11g03540 | XOO | | 1.569 | 0.0355 |  | AP2-EREBP | AP2 domain containing protein, expressed | | LOC_Os02g47660 | XOO | | 1.533 | 0.0086 |  | bHLH | basic helix-loop-helix, putative, expressed | | LOC_Os02g43820 | XOO | | 1.533 | 0.0417 | Drought | AP2-EREBP | AP2 domain containing protein, expressed | | LOC_Os03g53050 | XOO | | 1.512 | 0.0431 |  | WRKY | WRKY121, expressed | | LOC_Os05g34830 | XOO | | 1.471 | 0.0155 | Drought | NAC | No apical meristem protein, putative, expressed | | LOC_Os08g06120 | XOO | | 1.449 | 0.0077 |  | ABI3VP1 | B3 DNA binding domain containing protein, expressed | | LOC_Os07g43530 | XOO | | 1.435 | 0.0231 |  | bHLH | helix-loop-helix DNA-binding domain containing protein, expressed | | LOC_Os03g60080 | XOO | | 1.397 | 0.0303 |  | NAC | NAC domain-containing protein 67, putative, expressed | | LOC_Os11g06770 | XOO | | 1.334 | 0.022 |  | AP2-EREBP | ethylene-responsive transcription factor ERF110, putative, expressed | | LOC_Os04g32620 | XOO | | 1.307 | 0.0364 |  | AP2-EREBP | ethylene-responsive transcription factor ERF114, putative, expressed | | LOC_Os10g42690 | XOO | | 1.29 | 0.008 |  | Jumonji | jmjC domain containing protein, expressed | | LOC_Os09g28354 | XOO | | 1.272 | 0.0485 |  | HSF | CPuORF39 - conserved peptide uORF-containing transcript, expressed | | LOC_Os01g74020 | BPH | | 5.622 | 0.004 | Low temperature | G2-like | MYB family transcription factor, putative, expressed | | LOC_Os01g53220 | BPH | | 3.387 | 0.0018 | Drought | HSF | HSF-type DNA-binding domain containing protein, expressed | | LOC_Os12g31748 | BPH | | 3.223 | 0.0001 |  | MADS | OsMADS20 - MADS-box family gene with MIKCc type-box, expressed | | LOC_Os01g61080 | BPH | | 2.832 | 0.0021 | Low temperature | WRKY | WRKY24, expressed | | LOC_Os03g01890 | BPH | | 2.812 | 0.0001 |  | HB | START domain containing protein, expressed | | LOC_Os03g10810 | BPH | | 2.774 | 0.0001 |  | GNAT | HLS, putative, expressed | | LOC_Os03g06850 | BPH | | 2.612 | 0.009 |  | ABI3VP1 | B3 DNA binding domain containing protein, expressed | | LOC_Os06g19444 | BPH | | 2.555 | 0.024 |  | C2C2-CO-like | CCT/B-box zinc finger protein, putative, expressed | | LOC_Os03g64260 | BPH | | 2.366 | 0.0241 |  | AP2-EREBP | AP2 domain containing protein, expressed | | LOC_Os08g01780 | BPH | | 2.349 | 0.0001 |  | AUX_IAA | OsIAA25 - Auxin-responsive Aux/IAA gene family member, expressed | | LOC_Os02g12310 | BPH | | 2.223 | 0.042 |  | NAC | no apical meristem protein, putative, expressed | | LOC_Os03g10210 | BPH | | 2.169 | 0.0065 | Drought | HB | homeobox domain containing protein, expressed | | LOC_Os09g33550 | BPH | | 2.155 | 0.0016 |  | C2C2-CO-like | CCT/B-box zinc finger protein, putative, expressed | | LOC_Os06g44010 | BPH | | 2.125 | 0.0056 |  | WRKY | WRKY28, expressed | | LOC_Os02g49230 | BPH | | 2.12 | 0.0041 |  | C2C2-CO-like | CCT/B-box zinc finger protein, putative, expressed | | LOC_Os09g04890 | BPH | | 2.056 | 0.0068 |  | PHD | histone-lysine N-methyltransferase, H3 lysine-4 specific ATX1, putative, expressed | | LOC_Os03g21710 | BPH | | 2.055 | 0.0226 | Low temperature | WRKY | WRKY79, expressed | | LOC_Os07g22770 | BPH | | 2.04 | 0.0102 |  | AP2-EREBP | AP2 domain containing protein, expressed | | LOC_Os02g43790 | BPH | | 2.036 | 0.0008 | Low temperature | AP2-EREBP | ethylene-responsive transcription factor, putative, expressed | | LOC_Os12g39400 | BPH | | 2.012 | 0.0029 | Drought | C2H2 | ZOS12-09 - C2H2 zinc finger protein, expressed | | LOC_Os01g06640 | BPH | | 1.99 | 0.0055 |  | bHLH | basic helix-loop-helix, putative, expressed | | LOC_Os08g13840 | BPH | | 1.972 | 0.0051 |  | WRKY | WRKY25, expressed | | LOC_Os08g09900 | BPH | | 1.965 | 0 |  | WRKY | WRKY118, expressed | | LOC_Os02g56120 | BPH | | 1.964 | 0.0021 | Drought | AUX_IAA | OsIAA9 - Auxin-responsive Aux/IAA gene family member, expressed | | LOC_Os08g42470 | BPH | | 1.952 | 0.0032 |  | bHLH | BEE 1, putative, expressed | | LOC_Os10g41330 | BPH | | 1.939 | 0.0114 | Low temperature | AP2-EREBP | AP2 domain containing protein, expressed | | LOC_Os03g21800 | BPH | | 1.933 | 0.0089 |  | bZIP | bZIP transcription factor family protein, putative, expressed | | LOC_Os03g14850 | BPH | | 1.902 | 0.0036 | Low temperature | MADS | OsMADS72 - MADS-box family gene with M-alpha type-box, expressed | | LOC_Os05g37190 | BPH | | 1.9 | 0.0243 | Low temperature | C2H2 | ZOS5-08 - C2H2 zinc finger protein, expressed | | LOC_Os07g48560 | BPH | | 1.865 | 0.013 |  | HB | homeobox domain containing protein, expressed | | LOC_Os08g01700 | BPH | | 1.848 | 0.0074 | Submergance | bHLH | basic helix-loop-helix family protein, putative, expressed | | LOC_Os04g33870 | BPH | | 1.82 | 0.0166 |  | OFP | DUF623 domain containing protein, expressed | | LOC_Os06g16370 | BPH | | 1.82 | 0.0136 |  | C2C2-CO-like | CCT/B-box zinc finger protein, putative, expressed | | LOC_Os09g28440 | BPH | | 1.809 | 0.0461 | Low temperature | AP2-EREBP | AP2 domain containing protein, expressed | | LOC_Os09g21180 | BPH | | 1.785 | 0.0169 | Drought | HB | homeobox associated leucine zipper, putative, expressed | | LOC_Os10g35660 | BPH | | 1.751 | 0.0284 |  | MYB | MYB family transcription factor, putative, expressed | | LOC_Os05g46370 | BPH | | 1.721 | 0.0252 | Low temperature | bHLH | bHelix-loop-helix transcription factor, putative, expressed | | LOC_Os01g59350 | BPH | | 1.702 | 0.0006 |  | bZIP | transcription factor, putative, expressed | | LOC_Os07g27800 | BPH | | 1.692 | 0.002 |  | FAR1 | transposon protein, putative, unclassified, expressed | | LOC_Os03g08320 | BPH | | 1.671 | 0.039 | Low temperature | Tify | ZIM domain containing protein, putative, expressed | | LOC_Os03g13600 | BPH | | 1.646 | 0.0018 |  | C2H2 | ZOS3-07 - C2H2 zinc finger protein, expressed | | LOC_Os10g39750 | BPH | | 1.643 | 0.0165 |  | bHLH | inducer of CBF expression 2, putative, expressed | | LOC_Os08g33940 | BPH | | 1.624 | 0.007 |  | MYB | MYB family transcription factor, putative, expressed | | LOC_Os05g34830 | BPH | | 1.624 | 0.0165 | Drought | NAC | No apical meristem protein, putative, expressed | | LOC_Os08g42440 | BPH | | 1.62 | 0.0102 |  | C2C2-CO-like | CCT/B-box zinc finger protein, putative, expressed | | LOC_Os02g07450 | BPH | | 1.614 | 0.0015 |  | CCAAT | core histone H2A/H2B/H3/H4, putative, expressed | | LOC_Os06g07820 | BPH | | 1.607 | 0 |  | BSD | BSD domain containing protein, expressed | | LOC_Os05g37170 | BPH | | 1.595 | 0.0023 |  | bZIP | transcription factor, putative, expressed | | LOC_Os03g46790 | BPH | | 1.571 | 0.0132 |  | bHLH | helix-loop-helix DNA-binding domain containing protein, expressed | | LOC_Os11g01550 | BPH | | 1.565 | 0.0496 |  | LOB | DUF260 domain containing protein, putative, expressed | | LOC_Os02g41450 | BPH | | 1.537 | 0.0194 |  | NAC | no apical meristem protein, putative, expressed | | LOC_Os12g41650 | BPH | | 1.533 | 0.01 |  | bHLH | helix-loop-helix DNA-binding domain containing protein, expressed | | LOC_Os04g46220 | BPH | | 1.475 | 0.0161 |  | AP2-EREBP | ethylene-responsive transcription factor, putative, expressed | | LOC_Os03g58250 | BPH | | 1.458 | 0.0201 |  | bZIP | bZIP transcription factor domain containing protein, expressed | | LOC_Os08g37920 | BPH | | 1.455 | 0.003 |  | C2H2 | ZOS8-09 - C2H2 zinc finger protein, expressed | | LOC_Os06g11860 | BPH | | 1.451 | 0.0262 |  | AP2-EREBP | ethylene-responsive transcription factor, putative, expressed | | LOC_Os01g53880 | BPH | | 1.434 | 0.0272 |  | AUX_IAA | OsIAA6 - Auxin-responsive Aux/IAA gene family member, expressed | | LOC_Os04g49450 | BPH | | 1.42 | 0.0421 | Drought | MYB-related | MYB family transcription factor, putative, expressed | | LOC_Os07g18050 | BPH | | 1.386 | 0.0275 |  | C3H | RNA-binding motif protein, putative, expressed | | LOC_Os08g28214 | BPH | | 1.316 | 0.0405 |  | CPP | tesmin/TSO1-like CXC domain containing protein, expressed | | LOC_Os02g46610 | BPH | | 1.267 | 0.0212 |  | PLATZ | zinc-binding protein, putative, expressed | | LOC_Os08g43550 | BPH | | 1.231 | 0.0424 |  | MYB | MYB family transcription factor, putative, expressed | | LOC_Os02g55320 | BPH | | 1.219 | 0.0491 |  | ARR-B | two-component response regulator, putative, expressed | | LOC_Os04g40930 | BPH | | 1.179 | 0.0362 |  | Trihelix | MYB family transcription factor, putative, expressed | |

**Table S8.** Information on hormone treatment-induced transcription factor and transcription regulator genes.

| **Locus_ID Hormone treatment Fold change p-value Abiotic/Biotic Family RGAP Annotation**  **stress response** |
| --- |
[truncated: 205,336 more chars]
